# Spike Pattern Structure Influences Synaptic Efficacy Variability under STDP and Synaptic Homeostasis. I: Spike Generating Models on Converging Motifs

**DOI:** 10.3389/fncom.2016.00014

**Published:** 2016-02-23

**Authors:** Zedong Bi, Changsong Zhou

**Affiliations:** ^1^State Key Laboratory of Theoretical Physics, Institute of Theoretical Physics, Chinese Academy of Sciences Beijing, China; ^2^Department of Physics, Hong Kong Baptist University Kowloon Tong, Hong Kong; ^3^Centre for Nonlinear Studies, Beijing-Hong Kong-Singapore Joint Centre for Nonlinear and Complex Systems, Institute of Computational and Theoretical Studies, Hong Kong Baptist University Kowloon Tong, Hong Kong; ^4^Beijing Computational Science Research Center Beijing, China; ^5^Research Centre, HKBU Institute of Research and Continuing Education Shenzhen, China

**Keywords:** spike pattern structure, synaptic plasticity, efficacy variability, STDP, synaptic homeostasis, spike generating models

## Abstract

In neural systems, synaptic plasticity is usually driven by spike trains. Due to the inherent noises of neurons and synapses as well as the randomness of connection details, spike trains typically exhibit variability such as spatial randomness and temporal stochasticity, resulting in variability of synaptic changes under plasticity, which we call *efficacy variability*. How the variability of spike trains influences the efficacy variability of synapses remains unclear. In this paper, we try to understand this influence under pair-wise additive spike-timing dependent plasticity (STDP) when the mean strength of plastic synapses into a neuron is bounded (synaptic homeostasis). Specifically, we systematically study, analytically and numerically, how four aspects of statistical features, i.e., synchronous firing, burstiness/regularity, heterogeneity of rates and heterogeneity of cross-correlations, as well as their interactions influence the efficacy variability in converging motifs (simple networks in which one neuron receives from many other neurons). Neurons (including the post-synaptic neuron) in a converging motif generate spikes according to statistical models with tunable parameters. In this way, we can explicitly control the statistics of the spike patterns, and investigate their influence onto the efficacy variability, without worrying about the feedback from synaptic changes onto the dynamics of the post-synaptic neuron. We separate efficacy variability into two parts: the drift part (DriftV) induced by the heterogeneity of change rates of different synapses, and the diffusion part (DiffV) induced by weight diffusion caused by stochasticity of spike trains. Our main findings are: (1) synchronous firing and burstiness tend to increase DiffV, (2) heterogeneity of rates induces DriftV when potentiation and depression in STDP are not balanced, and (3) heterogeneity of cross-correlations induces DriftV together with heterogeneity of rates. We anticipate our work important for understanding functional processes of neuronal networks (such as memory) and neural development.

## 1. Introduction

Neuronal spike trains typically exhibit spatial randomness and temporal stochasticity. For example, firing rates are long-tailed distributed in many brain areas (Shafi et al., 2007; O'Connor et al., 2010; Buzsáki and Mizuseki, 2014), spatio-temporal correlations within neuronal population often exhibit rich structures (Funahashi and Inoue, 2000; Kohn and Smith, 2005; Dragoi and Buzsáki, 2006; Schneidman et al., 2006); and two neurons will not emit the same spike train even if they are receiving exactly the same stimuli (Allen and Stevens, 1994; Mainen and Sejnowski, 1995; Shadlen and Newsome, 1998). The spatial randomness may emerge from the randomness of the connection details (Ostojic et al., 2009; Roxin et al., 2011), and the temporal stochasticity may be due to the inner stochasticity of neurons and synapses (Allen and Stevens, 1994; Mainen and Sejnowski, 1995; Shadlen and Newsome, 1998), both of which are inherent properties of neurons, synapses or networks so that the exact spike patterns of the network cannot be fully determined by its inputs. Note that the inputs of a network may exhibit spatial heterogeneity and temporal fluctuation; here, by spatial randomness and temporal stochasticity, we mean the spatial heterogeneity and temporal fluctuation of spike patterns that emerge from the inner randomness and stochasticity of the network. As synaptic plasticity depends on the spike times in the pre- and post-synaptic spike trains (Dan and Poo, 2006; Caporale and Dan, 2008; Markram et al., 2012), these variabilities of spike trains should result in the variability of synaptic changes during plasticity, which we call *efficacy variability* in this paper (See Section 2.1 for more discussions on efficacy variability). When the synapses are fixed, downstream neurons may work under the variability of spike trains by reading out the coded information through spatial and temporal averaging; however, how the variability of the spike trains influences the ability of the neuronal population to facilitate information processing under synaptic plasticity remains poorly understood.

**Figure 1:**
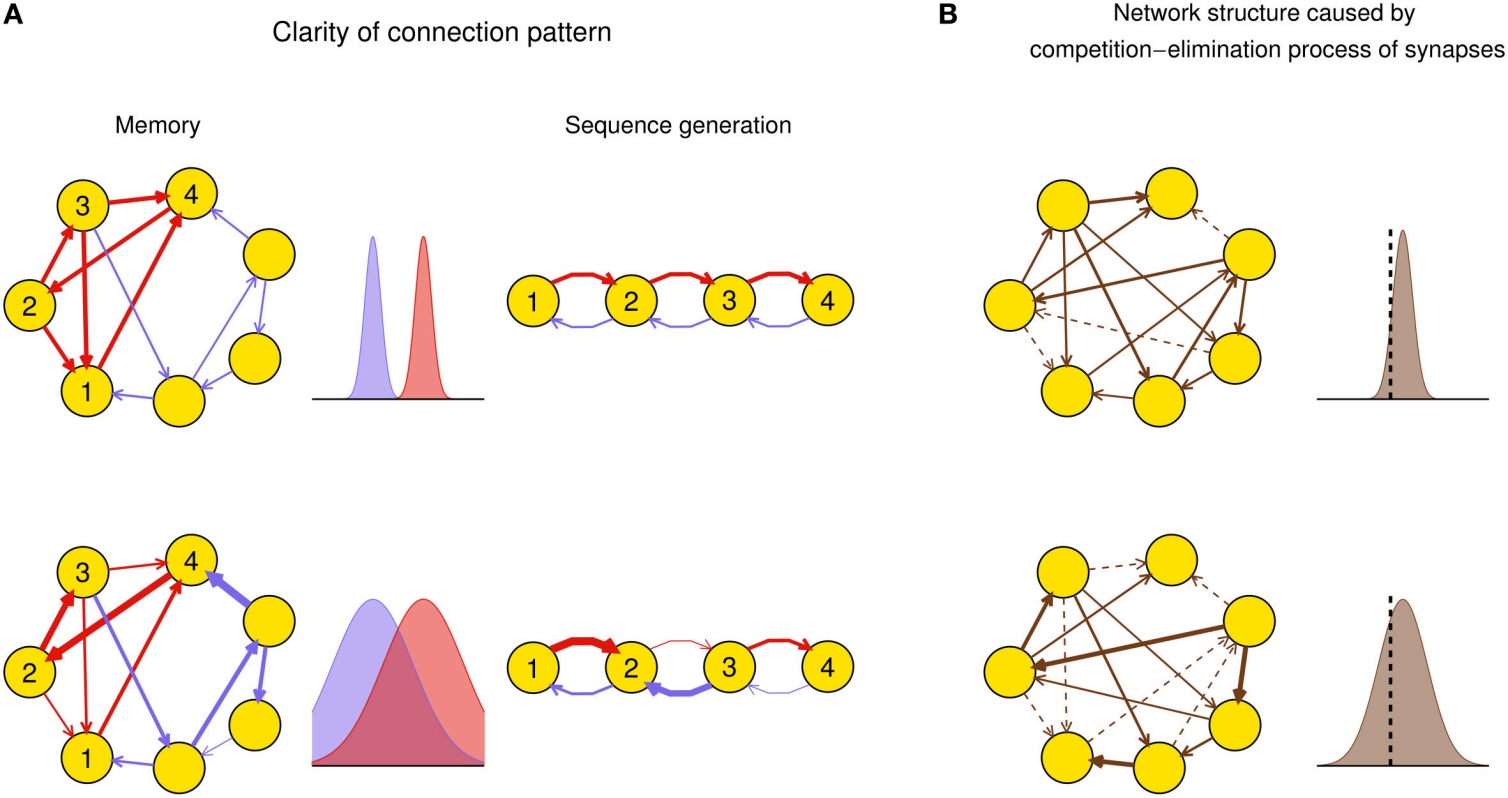
**Biological implications of efficacy variability**. **(A)** Connection patterns used for, say, memory (left column), or spike sequence generation (right column) are defined as a few synapses (red) being stronger than the others (blue). In the left column, a network of excitatory neurons stores a memory using its attractor dynamics after the intra-connections within a subpopulation (here, neurons 1–4) are strengthened (the inhibitory population that keeps the total activity of the network is not shown). When the efficacy variability is small (upper row), this subpopulation will exhibit persistently high activity if a sufficiently large number of neurons in the subpopulation have high activities initially, so the memory is retrieved. When the efficacy variability is large (lower row), this memory retrieval will fail even if the mean strength of the intra-connections (red) is stronger than that of the other ones (blue). In the right column, when the efficacy variability is small (upper row), the network is able to generate spike sequence from neuron 1 to neuron 4; but when the efficacy variability is large (lower row), it cannot generate such sequence even if the mean strength of the red synapses is larger than that of the blue ones. Widths of arrows indicate synaptic strengths. **(B)** Efficacy variability causes different network structures by controlling the degree of synaptic competition. When the efficacy variability is small (upper), only a few synapses are weaker than the elimination threshold (black dashed vertical line) and get eliminated during neural development, so most synapses are left and their strengths tend to be uniform; when the efficacy variability is large (lower), more synapses are eliminated, and the left ones are more heterogeneous and also stronger than the upper case on average. Dashed arrows represent eliminated synapses.

Efficacy variability may have important influence on the function of a network after plasticity. For example, suppose a function of a neuronal network, say memory (Mongillo et al., 2008) or spike sequence generation (Long et al., 2010), requires a connection pattern in which a few synapses (foreground synapses) have stronger efficacies than the others (background synapses). When the efficacy variability is small, both the foreground and background synapses tend to be uniform around their mean values, respectively: thus the connection pattern is clear-cut. However, when the efficacy variability is large, some foreground synapses can be very weak and some background ones can be very strong, which destroys the connection pattern even if the mean strength of the foreground synapses is still larger than that of the background ones (Figure 1A). As another example, synaptic competition and elimination is a classical scenario for the formation of neural network structure during development, when synapses compete with each other for strength and those that are too weak will disappear (Cancedda and Poo, 2009). In this case, efficacy variability quantifies the degree of competition. If we suppose that the total synaptic strength before elimination is constrained by, say, synaptic homeostasis (Watt and Desai, 2010), then when the efficacy variability is small, only a few synapses are below the elimination threshold and get eliminated, and those left also have similar strength; when the efficacy variability is large, a larger portion of synapses get below the elimination threshold, while the remaining ones have a wider efficacy distribution with also a larger mean value than the case of small efficacy variability (Figure 1B). This is consistent with the scenario found in the early development of auditory cortex (Clause et al., 2014): if the spontaneous activity of the medial nucleus of the trapezoid body (MNTB) is modified using genetic methods, then its feedforward projection to the lateral superior olive (LSO) becomes denser and weaker, which suggests that the normal pattern induces stronger efficacy variability than the genetically modified one.

**Figure 2:**
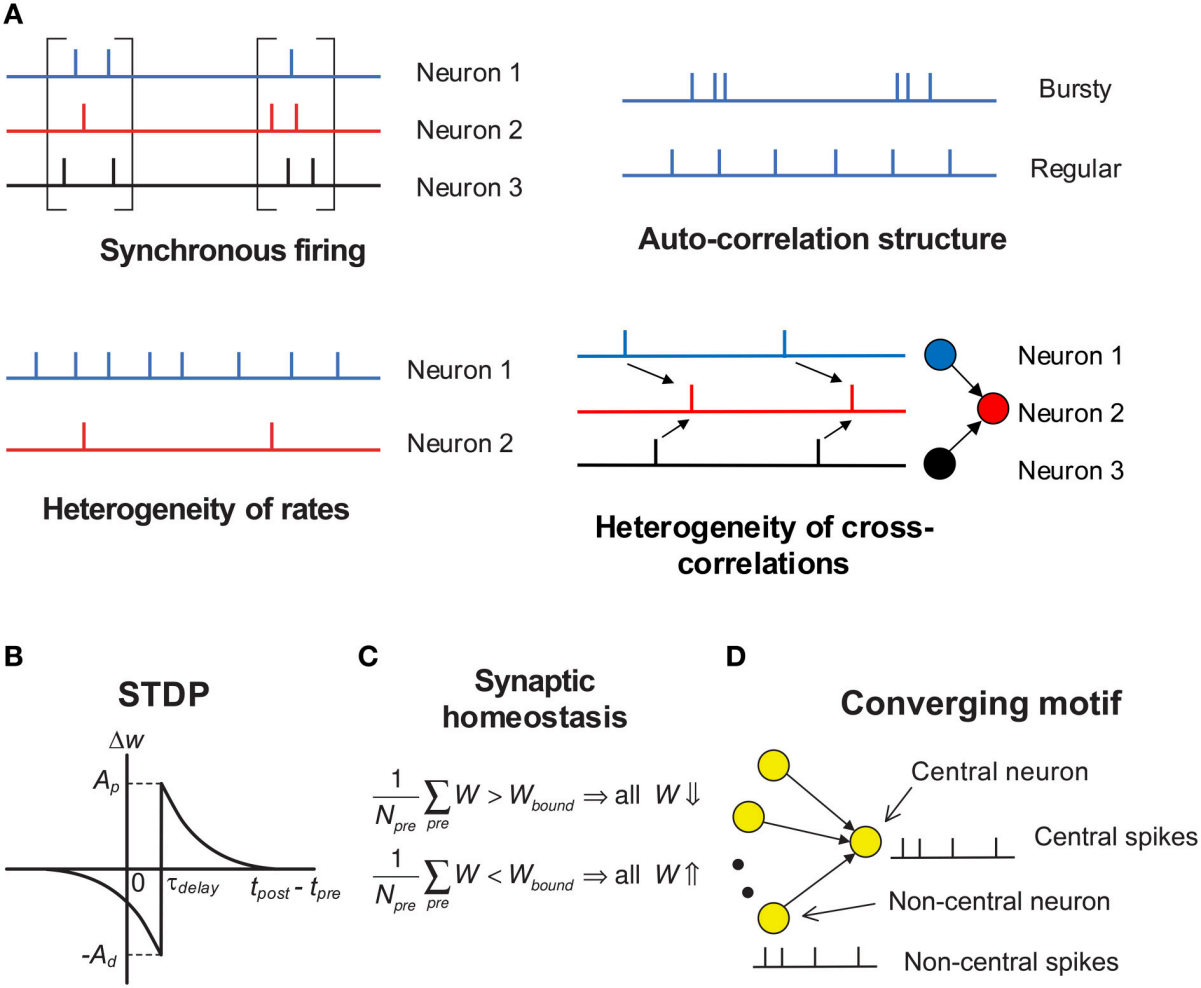
**Schematic of key concepts in our modeling work**. **(A)** The four aspects of pattern structure studied in this paper. By “synchronous firing”, we typically mean the spurt of firing activity of a population. For stationary spike trains, “auto-correlation structure” reflects the burstiness/regularity of the spike trains, which is quantified by coefficient of variance (*CV*) in this paper. Here, by “burstiness”, we typically mean the irregular structure of spike trains, instead of the regular burstiness in the spike patterns of, say, central pattern generator. For spike trains with synchronous firing, we consider three-types of “auto-correlation structure” to reflect the burstiness/regularity features of the spike patterns (see **Figure 8**). By “heterogeneity of rates”, we mean that the time-averaged firing rates are different for different neurons. By “heterogeneity of cross-correlations”, we mean that different pre-synaptic neurons of a neuron tends to fire spikes at different times relative to the spikes of the neuron. For example, in the right-bottom subplot, before a spike of neuron 2, neuron 1 tends to fire before neuron 3. **(B)** The STDP time window used in our work. The synapses are updated according to the time difference between when the post-synaptic spike is back-propagated to the dendritic end and when the pre-synaptic spike arrives at the axonal terminal. τ_*delay*_ is the difference between the axonal delay and the dendritic delay. In this paper, we generally discuss the case when τ_*delay*_ > 0 (i.e., the axonal delay is larger than the dendritic delay); and for the convenience of discussion, we set the dendritic delay to be zero, so τ_*delay*_ is the axonal delay. The case when τ_*delay*_ < 0 is then discussed in Section 3.8. The STDP updatings of all spike pairs are summed together. **(C)** Synaptic homeostasis. The synapses input to a neuron are subject to a bound on their mean strength: when their mean strength is different from this bound, all the incoming synapses of that neuron will undergo an adjustment. **(D)** Converging motif, on which we conduct all the simulations in this paper. We call the neuron that receives from many neurons in a converging motif to be the *central neuron*, and call the other neurons the *non-central neurons*; we then call the spikes emitted by the central neuron (or non-central neurons) to be *central spikes* (or *non-central spikes*). Synaptic homeostasis is imposed onto the central neuron of a converging motif. Modeling details are presented in Section 2.

Under temporal stochasticity and spatial heterogeneity, spike trains may exhibit a variety of statistical features, which form rich spike pattern structures. Groups of neurons may spurt firing activity (*synchronous firing*) (Kamioka et al., 1996; Buzsáki and Draguhn, 2004; Bartos et al., 2007), the spike train of a single neuron can be bursty or regular (*auto-correlation structure*) (Softky and Koch, 1993; Schwindt and Crill, 1999; Jacob et al., 2012), firing rates of cortical neurons are typically long-tailed distributed *in vivo* (*heterogeneity of rates*) (Shafi et al., 2007; O'Connor et al., 2010; Buzsáki and Mizuseki, 2014), and spike trains of different neurons also reveal rich degrees of interdependence (*heterogeneity of cross-correlations*) (Funahashi and Inoue, 2000; Schneidman et al., 2006; Ostojic et al., 2009; Trousdale et al., 2012, see Figure 2A). As synaptic plasticity is driven by spike trains, spike pattern structure must have strong influence on efficacy variability, thereby inducing neuronal networks with sharply different structures even under the same population rate. How spike pattern structures influence the efficacy variability of synapses remains unclear. In this study, we will move a step forward along this direction by studying this influence under a conventional pair-wise additive STDP (Gerstner et al., 1996), which involves potentiation of the synapse when presynaptic spikes precede postsynaptic spikes, and depression for the reverse ordering (Figure 2B). Neuronal networks with STDP alone are not stable due to effects of positive resonance which results in runaway excitation; and a neuron may conserve its activity level by adjusting its total input strength, which is called synaptic homeostasis (Turrigiano and Nelson, 2004; Turrigiano, 2011). In this study, we model synaptic homeostasis by supposing that the mean strength of all the synapses input to a neuron is dynamically bounded (see Figure 2C, Equation 7 in Section 2).

Synaptic efficacies under STDP can be regarded as particles doing 1-dimensional random walks driven by the stochastic processes of spike trains: a synapse gets depressed at the time when a pre-synaptic spike arrives at the axonal terminal, and gets potentiated at the time when a post-synaptic spike is back-propagated to the dendritic end. A group of particles doing random walks starting from the same point can have displacement variability either because that they have different trial-averaged drift velocities with each other, or because of diffusion. Similarly, the efficacies of two synapses can also get dissimilar because of two reasons. One is that they have different tendencies to be potentiated or depressed. For example, the first (second) pre-synaptic neuron tends to fire spikes before (after) the first (second) post-synaptic neuron. The other reason is the variability of the synaptic changes caused by the stochasticity of the spike trains, which can be accumulated with time, inducing dissimilarity even if the two synapses have the same tendency to be potentiated or depressed. In general, the total variance (TotalV) can be written as the summation of the variance caused by heterogeneity of learning rates (DriftV, short for “drift variance”) and the variance caused by diffusion (DiffV, short for “diffusion variance”) (see Equation 3 in Section 2)


(1) $$\begin{array}{l} \text{Total}V=\text{DriftV}+\text{DiffV}. \end{array}$$

During plasticity, DriftV is usually caused by the spatial heterogeneity of spike trains. For example, in classical Hebbian learning, synapses sharing the same presynaptic neuron may have different learning rates depending on the firing rates of the post-synaptic neurons; if the plasticity is spike-timing dependent, the heterogeneity of cross-correlations can induce different learning rates even if the firing rates are the same. Because of the inner stochasticity of neurons and synapses, even two neurons receiving exactly the same stimuli emit different spike trains, causing DiffV. In our STDP model, synaptic updatings do not depend on current synaptic weights, and the contributions of all spike pairs are added together; what's more, we do not consider synaptic bounds, and assume that synapses are free of bounds for simplicity. These make DriftV ∝ *t*^2^ and DiffV ∝ *t* during time *t* of running, so that the synaptic variances caused by drift velocities and diffusion in our model have the same order of time-dependence as those in Brownian motion.

In this paper, we systematically study how the four aspects of spike pattern structures, i.e., synchronous firing, auto-correlation structure, heterogeneity of rates and heterogeneity of cross-correlations as well as their interactions, influence efficacy variability under STDP and synaptic homeostasis using converging motifs (i.e., simple networks in which one neuron receives from many other neurons). See Figure 2 for the concepts above. The activities of all the neurons in converging motifs (including the non-central neurons and the central neuron, see Figure 2D) are generated using statistical models (Section 2.3), which explicitly control different aspects of pattern structure while keeping population rate constant.

The reason why we study converging motifs here is that in a recurrent network, all the synapses input to a neuron form the links of a converging motif, and all the synapses of the recurrent network can be considered as the union of the links in all the converging motifs. Under synaptic homeostasis, the mean synaptic strengths in these converging motifs are almost the same, therefore, the efficacy variability of the recurrent network is approximately to be the mean of the efficacy variability of all the converging motifs in it (see Equation 8 in Section 2). Therefore, converging motifs are basic units to understand the efficacy variability of a recurrent network.

Using statistical models (see Section 2.3), we generate spike patterns with different statistical features (such as coefficients of variance (*CV*), firing rate distributions, distributions of the spike number that a neuron fires during a synchronous event, and so on), and then study how they influence the efficacy variability of converging motifs. These spike generating models do not aim to generate spike trains with precise pre-determined spatio-temporal characteristics (see, for example, Krumin and Shoham, 2009; Macke et al., 2009; Gutnisky and Josić, 2010), but instead aim to implement physical intuitions on spatio-temporal characteristics in a simple way. For example, we use Gamma processes with different shape parameters to model bursty or regular spike trains, and use lognormal distributions to model heterogeneity of rates. Note that in our study, the spike trains of the central neuron are not generated by integrating its inputs, but instead are also generated according to statistical models, independent of the synaptic weights in converging motifs. In this way, we can explicitly control the statistical features of both the central and non-central spike trains using statistical models, and study their influences onto the efficacy variability without worrying about the feedback of the synaptic changes onto the spike train of the post-synaptic neuron as usually happens in biologically more realistic models.

The spike trains of both the central and non-central neurons are generated according to the same model, so that they have similar auto-correlation structure and synchronous firing rate fluctuation. This makes our results particularly suitable to understand the efficacy variability of a recurrent network without hidden feedforward dynamics (Ganguli and Latham, 2009). For example, in a random excitatory-inhibitory network that works in the balanced state (van Vreeswijk and Sompolinsky, 1996, 1998), spike trains exhibit irregularity and stochasticity due to being driven by fluctuation. If the dynamics of the network is unstable to perturbations of population firing rates, then oscillation may emerge (Brunel and Hakim, 1999; Brunel, 2000), resulting in synchronous firing (Figure 2A). If its dynamics is unstable to heterogeneous perturbations, then the neurons may fire with strong burstiness (Ostojic, 2014) (auto-correlation structure). Due to the heterogeneity of the input degrees and nonlinear current-rate relationship of neurons, their firing rates are typically long-tailed distributed (Roxin et al., 2011) (heterogeneity of rates). Cross-correlations between neurons may result from connectivity details such as unidirectional connections or input sharing (Ostojic et al., 2009) (heterogeneity of cross-correlations). In this paper, we aim to mimick these spike patterns using statistical models, thereby gaining understanding on their influence onto the efficacy variability of a network. In another paper that will be published soon (Bi and Zhou, in preparation), we will study the influence of spike pattern structures onto the efficacy variability of recurrent networks by implementing sophisticated spike shuffling methods onto the spike patterns self-organized by recurrent LIF networks, thereby examining the results of this paper in a biologically more plausible manner.

The Results part of this paper is organized as follows. We will first study how auto-correlation structure, synchronous firing, heterogeneity of rates and their interactions influence DiffV and DriftV (Sections 3.1–3.6), then discuss the influence of heterogeneity of cross-correlations onto DriftV (Section 3.7). Under STDP, the main effect of heterogeneity of cross-correlations is to change the drift velocities of different synapses by different degrees, thereby influencing DriftV; so we don't seriously consider how heterogeneity of cross-correlations influence DiffV, except for briefly discussing it in Supplementary Materials Section S6. In this paper, we generally discuss the case when τ_*delay*_ > 0 in STDP (i.e., the axonal delay is larger than the dendritic delay, see Figure 2B); and for the convenience of discussion, we set the dendritic delay to be zero, so τ_*delay*_ becomes the axonal delay. The case when τ_*delay*_ < 0 is then discussed in Section 3.8. The results of this paper are then summarized in Section 3.9.

## 2. Materials and methods

### 2.1. The definition of efficacy variability

In this subsection, we give the exact definition of efficacy variability, explain the meanings of DiffV and DriftV, and show our general strategy to study efficacy variability using simulations.

Suppose a set of synapses W in a neuronal network N. Now we run a plasticity process in N for several trials (or on several ensembles), and construct a matrix Δ**W**, each column of which represents the weight changes of W in one trial, and different columns represent different trials. Then we define *efficacy variability* of W during the plasticity process to be the variance of the elements of the matrix Δ**W**, i.e., Var_*S, T*_(Δ**W**), in which the subscript *S* represents integrating over row index, i.e., structural index, and *T* represents integrating over column index, i.e., trial index.

The law of total variance says that if the probability space of *Y* is decomposed into several subspaces labeled by *X*, then the variance of *Y* in the whole space is equal to the summation of the variance of the expectations in these subspaces and the expectation of the variances in these subspaces, i.e.,


(2) $$\begin{array}{l} \text{Var}\left(Y\right)=\text{Var}\left(\text{E}\left(Y|X\right)\right)+\text{E}\left(\text{Var}\left(Y|X\right)\right) \end{array}$$

Using the law of total variance, efficacy variability can be written as


(3) $$\begin{array}{l} {\text{Var}}_{S,T}\left(\Delta \text{W}\right)={\text{Var}}_{S}\left({\text{E}}_{T}\left(\Delta \text{W}\right)\right)+{\text{E}}_{S}\left({\text{Var}}_{T}\left(\Delta \text{W}\right)\right) \end{array}$$

Here, E_*T*_(Δ**W**) represents the trial expectations of the changes of all the synapses in W; and Var_*S*_(E_*T*_(Δ**W**)) is the variance of these trial expectations, representing DriftV. Var_*T*_(Δ**W**) represents the trial-to-trial variances caused by diffusion, and E_*S*_(Var_*T*_(Δ**W**)) is the average of these variances over all the synapses, representing DiffV. Equation 3 is the formal writing of Equation 1 in the introduction.

The law of total variance can decompose Var_*S, T*_(Δ**W**) in another way:


(4) $$\begin{array}{l} {\text{Var}}_{S,T}\left(\Delta \text{W}\right)={\text{Var}}_{T}\left({\text{E}}_{S}\left(\Delta \text{W}\right)\right)+{\text{E}}_{T}\left({\text{Var}}_{S}\left(\Delta \text{W}\right)\right) \end{array}$$

Here Var_*T*_(E_*S*_(Δ**W**)) is the trial-to-trial variability of the mean synaptic change of the whole network. But a real biological process only allows a single trial, so this trial-to-trial variability cannot contribute to biological functions except for individual differences. Fortunately, usually Var_*T*_(E_*S*_(Δ**W**)) ~ O(1∕|W|), with |W| being the number of synapses in W. So when |W| is large enough, Equation 4 becomes Var_*S, T*_(Δ**W**) ≈ E_*T*_(Var_*S*_(Δ**W**)), which means that the trial expectation of the efficacy variance (i.e., E_*T*_(Var_*S*_(Δ**W**))) can be used to approximate the efficacy variability Var_*S, T*_(Δ**W**). Under this insight, Equation 3 becomes


(5) $$\begin{array}{rcl} E_{T}\left({\text{Var}}_{S}\left(\Delta \text{W}\right)\right)\approx {\text{Var}}_{S}\left({\text{E}}_{T}\left(\Delta \text{W}\right)\right)+{\text{E}}_{S}\left({\text{Var}}_{T}\left(\Delta \text{W}\right)\right)\\ \; & \;= & \text{DriftV}+\text{DiffV} \end{array}$$

In our simulations, we use E_*T*_(Var_*S*_(Δ**W**)) to quantify efficacy variability. As we mentioned in the introduction, DriftV is usually caused by the spatial heterogeneity of spike trains, and DiffV is by temporal stochasticity. Methodologically, when we analyze DiffV, we set the spatial properties of spike trains (here, firing rates of neurons and cross-correlations between neuronal pairs) homogeneous, so that DriftV = 0. When we analyze DriftV, we use the fact that DriftV ~ O(*t*^2^) and DiffV ~ O(*t*) after time *t* of running, so the efficacy variability after a sufficiently long time largely reflects DriftV.

### 2.2. Converging motifs, STDP and synaptic homeostasis

Converging motifs are simple networks in which one neuron receives inputs from many other neurons (Figure 2D). We call the neuron that receives from many neurons in a converging motif to be the *central neuron*, and call the other neurons the *non-central neurons*; we then call the spikes emitted by the central neuron (or non-central neurons) to be *central spikes* (or *non-central spikes*). All the simulations in this paper are conducted on converging motifs.

In this paper, the STDP updating caused by a pair of pre- and post-synaptic spike (here, non-central and central spike) at *t*_*pre*_ and *t*_*post*_ is

(6) $$\begin{array}{r} \Delta w(t_{pre},t_{post})=\{\begin{array}{l} A_{p}\exp(-\frac{t_{post}-(t_{pre}+\tau_{delay})}{\tau_{STDP}}),\\ \;t_{post}>t_{pre}+\tau_{delay}\\ -A_{d}\exp(-\frac{(t_{pre}+\tau_{delay})-t_{post}}{\tau_{STDP}}),\\ \;t_{post}<t_{pre}+\tau_{delay} \end{array} \end{array}$$

with τ_*delay*_ being the difference between the delay caused by the propagation of pre-synaptic spike along the axon and the delay caused by the back-propagation of the post-synaptic spike along the dendrite. τ_*delay*_ > 0 (or τ_*delay*_ < 0) if the axonal delay is larger (or smaller) than the dendritic delay. In this paper, we generally discuss the case when τ_*delay*_ > 0 (i.e., the axonal delay is larger than the dendritic delay); and for the convenience of discussion, we set the dendritic delay to be zero, so τ_*delay*_ becomes the axonal delay. The case when τ_*delay*_ < 0 is then discussed in Section 3.8. The contribution of all pairs of pre- and post-synaptic spikes are added together. τ_*STDP*_ = 20 ms throughout the paper, and τ_*delay*_ = 1ms by default. For simplicity, synaptic weights in this paper are free of boundaries.

Under synaptic homeostasis, the synaptic efficacies are updated every Δ*T* time according to

(7) $$\begin{array}{l} w_{a}\left(t+\Delta T\right)=w_{a}\left(t\right)+\epsilon \left(w_{bound}-\frac{1}{N_{in}}\overset{N_{in}}{\underset{c=1}{\sum }}w_{c}\left(t\right)\right), \end{array}$$

with *w*_*a*_ being the synaptic efficacy between the *a*th non-central neuron and the central neuron, *N*_*in*_ being the in-degree of central neuron, *w*_*bound*_ being the ground line of synaptic homeostasis, and ϵ being the plasticity rate. Therefore, synaptic homeostasis dynamically bounds the mean strength of the synapses to the central neuron, and it does not change the efficacy variability of a converging motif due to its additive nature.

According to the law of total variance Equation 2, the efficacy variability of a large recurrent network can be written as

(8) $$\begin{array}{l} {\text{Var}}_{ab}\left(\Delta w_{ab}\right)={\text{Var}}_{a}\left({\text{E}}_{b\in \partial a}\left(\Delta w_{ab}\right)\right)+{\text{E}}_{a}\left({\text{Var}}_{b\in \partial a}\left(\Delta w_{ab}\right)\right), \end{array}$$

with Δ*w*_*ab*_ being the synaptic change from neuron *b* to neuron *a*, and ∂*a* being all the neurons that input to neuron *a* in the network. If synaptic homeostasis Equation 7 is imposed onto the synapses input to each neuron in the network, Var_*a*_(E_*b*∈∂*a*_(Δ*w*_*ab*_)) ≈ 0, especially if the plasticity rate is high (i.e., ϵ ≈ 1 in Equation 7). Therefore, the efficacy variability of the recurrent network is approximately the mean of the efficacy variability of all the converging motifs in it. This is the reason why we study converging motifs in this paper.

### 2.3. Spike generating models

Here are the statistical models we use to generate the spike trains of the neurons in a converging motif (Figure 2D). The spike trains of the central neuron and the non-central neurons are generated according to the same model, mimicking the dynamics of converging motifs embedded in a recurrent network without hidden feedforward dynamics (Ganguli and Latham, 2009).

#### 2.3.1. Model auto

This model generates spike trains with stationary firing rate, whose auto-correlation structure (burstiness/regularity) can be controlled.

Spikes trains are Gamma processes with inter-spike intervals following the distribution


$$p\left(x|\alpha ,\beta \right)=\frac{1}{\Gamma \left(\alpha \right)\beta^{\alpha }}x^{\alpha -1}e^{-x∕\beta }$$

The rate of the Gamma process is β∕α, and the coefficient of variance is $1∕\sqrt{\alpha }$.

We use α to control the burstiness/regularity of the spike train, while adjusting β to keep the firing rate at 20 Hz. The spike train becomes more bursty when α is smaller, and more regular when α is large.

#### 2.3.2. Model sync

This model generates synchronous firing patterns whose broadness of the distribution of spike number per neuron per synchronous event can be explicitly controlled.

In this model, a synchronous event lasts for τ_*cross*_. The spike number per neuron per synchronous event is distributed the same as the spike number of an underlying Gamma process within an interval of length τ_*cross*_. The rate of the Gamma process is *p*∕τ_*cross*_ and its coefficient of variance is *CV*_*SpikeNum*_. So the mean value of the distribution of spike number per neuron per synchronous event is *p*, and this distribution is narrow if *CV*_*SpikeNum*_ is small, and gets broadened when *CV*_*SpikeNum*_ increases. If a neuron is to fire *M* spikes in a synchronous event, then the spike times of these *M* spikes will be randomly and uniformly chosen within this time interval of duration τ_*cross*_. The occurrence of synchronous events is a Poisson process with rate *r*_0_∕*p*, so that the firing rate of a neuron is kept at *r*_0_ = 20Hz when *p* changes.

#### 2.3.3. Model long tail

This model generates long-tailed distributed firing rates for the non-central neurons in a converging motif, the firing rate of the central neuron is always kept at *r*_0_ = 20Hz.

The firing rates of the non-central neurons are lognormal distributed as


$$p\left(x|m,s\right)=\frac{1}{sx\sqrt{2\pi }}\;\exp\;\left[-\frac{{\left(\ln\;x-m\right)}^{2}}{2s^{2}}\right]$$

The mean of this distribution is at $\exp\left(m+\frac{s^{2}}{2}\right)$. Parameter *s* is used to control the shape, while *m* is accordingly adjusted to keep the mean at *r*_0_ = 20Hz. This distribution is a δ function when *s* = 0, and gradually becomes long tailed when *s* increases.

This model generates homogeneous Poisson processes by default, but it can be also combined with other models to introduce heterogeneity of rates into the spike patterns with other aspects of pattern structure.

#### 2.3.4. Model sync-auto-longTail

In this model, the firing rates of the non-central neurons follow log-normal distribution with mean *r*_0_ = 20Hz (with *r*_0_ being the firing rate of the central neuron) and shape parameter *s*. A synchronous event lasts for τ_*cross*_, and the mean spike number per neuron per synchronous event is *p*. The occurrence of synchronous events is a Gamma process with coefficient of variance *CV*_*events*_ and rate *r*_0_∕*p*, so that the population firing rate is kept at *r*_0_ when *p* changes. The spike train of a neuron with firing rate *r*_*i*_ is generated as follows: we generate a Gamma process with rate *pr*_*i*_∕*r*_0_ and coefficient of variance *CV*_*rescale*_, and divide the Gamma spike train into bins of length τ_*cross*_. Then we randomly shuffle the order of these bins to destroy possible spike dependency between adjacent bins, and the piece of spike train within the *j*th bin after shuffling is to be the piece of spike train of the neuron within the *j*th synchronous event.

In this model, the auto-correlation structure of the occurrence of synchronous events (see **Figure 10**) is controlled by the parameter *CV*_*events*_, while the auto-correlation structure of a spike train in the rescaled time is controlled by the parameter *CV*_*rescale*_.

## 3. Results

### 3.1. The scheme to investigate the influence of synchronous firing and auto-correlation structure onto DiffV

**Figure 3:**
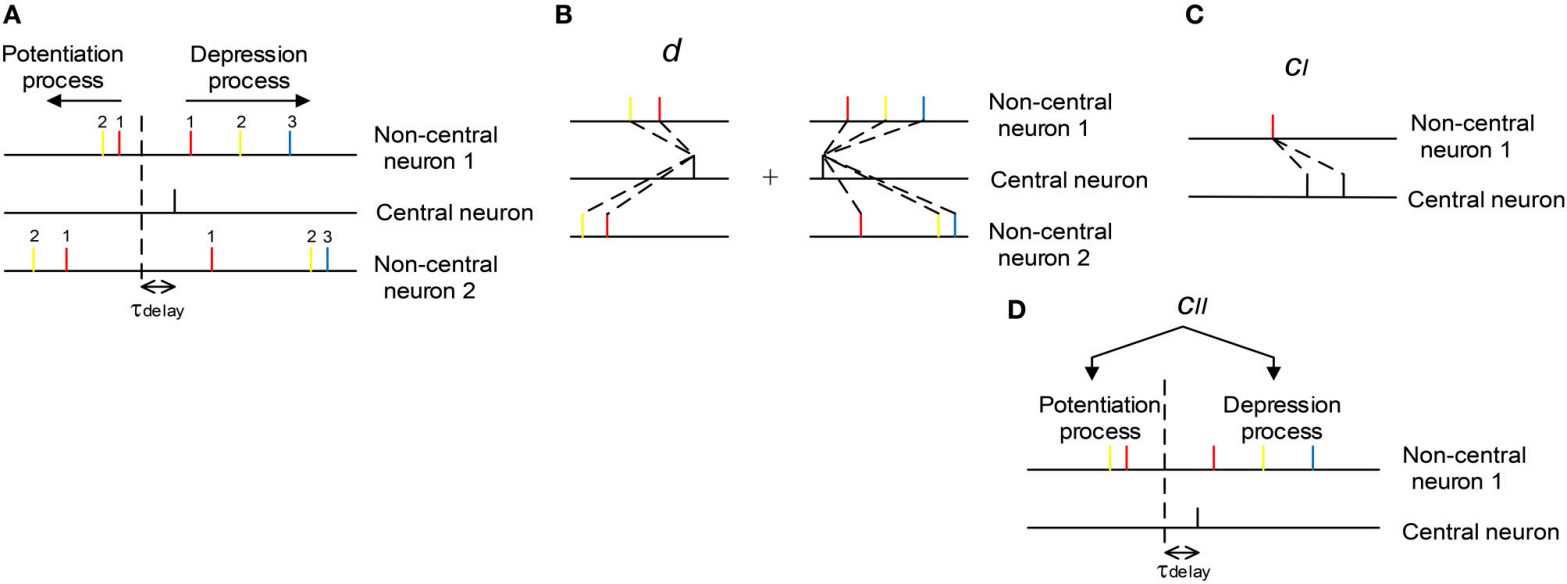
**The scheme we use to understand the influence of synchronous firing and auto-correlation structure onto DiffV**. **(A)** The indexing of *j* (see text). The non-central spikes of the same color have the same *j* index (indicated by the numbers). The dashed vertical line represents the separation of the potentiation and depression process caused by the black spike of the central neuron. Synapses between the central and non-central neurons have axonal delay τ_*delay*_. **(B)** *d* represents the mean efficacy variability caused by a single central spike in the potentiation process plus that in the depression process. **(C)** *c*_*I*_ represents the efficacy variability contributed by the correlation between synaptic changes caused by adjacent central spikes. **(D)** *c*_*II*_ represents the efficacy variability contributed by the correlation between the total potentiation and depression value imposed on the same synapse.

When the firing rates of neurons and the cross-correlations between neurons are homogeneous, synchronous firing and auto-correlation structure influence efficacy variability as a DiffV effect. To understand how these two pattern statistics influence DiffV, we write the total change of the *a*th synapse as

(9) $$\begin{array}{l} \Delta w_{a}=\underset{k=p,d}{\sum }\Delta w_{a,k}=\underset{k=p,d}{\sum }\underset{i}{\sum }\underset{j}{\sum }\Delta w_{a,k}\left(t_{i},t_{j|a,i,k}\right), \end{array}$$

with Δ*w*_*a, p*_ being the total potentiation value on the synapse, Δ*w*_*a, p*_(*t*_*i*_, *t*_*j*|*a, i, p*_) being the potentiation value pairing the *i*th spike of central neuron and the *j*th spike of the *a*th non-central neuron (the index of *j* depends on *a*, *i*, and *k*, see below), and Δ*w*_*a, d*_ and Δ*w*_*a, d*_(*t*_*i*_, *t*_*j*|*a, i, d*_) being the corresponding depression values. The index of *i* starts from the beginning of the spike train of the central neuron; but the indexing of *j*, however, is a little complicated, and depends on *a*, *i* and *k*. In our modeling work, axons have delay τ_*delay*_ (τ_*delay*_ = 1 ms by default), and the sign of STDP updatings depends on the time difference between *t*_*i*_ − τ_*delay*_ and *t*_*j*|*a, i, k*_. For the potentiation process (*k* = *p*) of the *a*th non-central neuron, we let *j* start from the spike immediately before *t*_*i*_ − τ_*delay*_ in the spike train, and go backward along the spike train; for the depression process (*k* = *d*), we let *j* start from the spike immediately after *t*_*i*_ − τ_*delay*_, and go forward along the spike train (Figure 3A). In the following discussions, we sometimes do not explicitly write the dependence of *j* on *a*, *i* and *k* for simplicity.

Using Equation 9, we can rewrite the variance of efficacy changes per spike like this:

(10) $$\begin{array}{l} {\text{Var}}_{a}\left(\Delta w_{a}\right)∕{\bar{N}}_{0}={\text{Var}}_{a}\left(\underset{k=p,d}{\sum }\underset{i}{\sum }\underset{j}{\sum }\Delta w_{a,k}\left(t_{i},t_{j}\right)\right)∕{\bar{N}}_{0}=c_{II}\cdot c_{I}\cdot d \end{array}$$

with ${\bar{N}}_{0}=r_{0}T$ being the expectation of the spike number of the central neuron during simulation time (*r*_0_ is the firing rate of the central neuron, and *T* is the duration of simulation), and

(11) $$\begin{array}{l} c_{II}=\frac{{\text{Var}}_{a}\left(\sum_{k}\sum_{i}\sum_{j}\Delta w_{a,k}\left(t_{i},t_{j}\right)\right)}{\sum_{k}{\text{Var}}_{a}\left(\sum_{i}\sum_{j}\Delta w_{a,k}\left(t_{i},t_{j}\right)\right)} \end{array}$$

(12) $$\begin{array}{l} c_{I}=\frac{\sum_{k}{\text{Var}}_{a}\left(\sum_{i}\sum_{j}\Delta w_{a,k}\left(t_{i},t_{j}\right)\right)}{\sum_{k}\sum_{i}{\text{Var}}_{a}\left(\sum_{j}\Delta w_{a,k}\left(t_{i},t_{j}\right)\right)} \end{array}$$

and

(13) $$\begin{array}{l} d=\underset{k}{\sum }\underset{i}{\sum }{\text{Var}}_{a}\left(\underset{j}{\sum }\Delta w_{a,k}\left(t_{i},t_{j}\right)\right)∕{\bar{N}}_{0} \end{array}$$

Apparently, *d* means the mean efficacy variability contributed by a single spike of the central neuron. To understand *c*_*II*_, note that

(14) $$\begin{array}{l} {\text{ Var}}_{a}(\Delta w_{a})=\sum_{k=p,d}{\text{Var}}_{a}(\sum_{i}\sum_{j}\Delta w_{a,k}(t_{i},t_{j}))\\ +2\rho_{PD}\sqrt{{\text{Var}}_{a}(\sum_{i}\sum_{j}\Delta w_{a,p}(t_{i},t_{j}))\cdot {\text{Var}}_{a}(\sum_{i}\sum_{j}\Delta w_{a,d}(t_{i},t_{j}))} \end{array}$$

with $\rho_{PD}={\text{Corr}}_{a}\left(\underset{i}{\sum }\underset{j}{\sum }\Delta w_{a,p}\left(t_{i},t_{j}\right),\underset{i}{\sum }\underset{j}{\sum }\Delta w_{a,d}\left(t_{i},t_{j}\right)\right)$ being the correlation coefficient between the total potentiation and depression value imposed on the same synapse. Therefore,

(15) $$\begin{array}{l} c_{II}=1+\rho_{PD}f_{PD} \end{array}$$

with

(16) $$\begin{array}{l} f_{PD}=\frac{2\sqrt{{\text{Var}}_{a}\left(\sum_{i}\sum_{j}\Delta w_{a,p}\left(t_{i},t_{j}\right)\right)\cdot {\text{Var}}_{a}\left(\sum_{i}\sum_{j}\Delta w_{a,d}\left(t_{i},t_{j}\right)\right)}}{\sum_{k}{\text{Var}}_{a}\left(\sum_{i}\sum_{j}\Delta w_{a,k}\left(t_{i},t_{j}\right)\right)} \end{array}$$

being the coupling factor. Similarly,

(17) $$\begin{array}{l} c_{I}=1+\underset{k}{\sum }\underset{m<n}{\sum }\rho_{m,n;k}f_{m,n;k} \end{array}$$

with $\rho_{m,n;k}={\text{Corr}}_{a}\left(\underset{j}{\sum }\Delta w_{a,k}\left(t_{m},t_{j}\right),\underset{j}{\sum }\Delta w_{a,k}\left(t_{n},t_{j}\right)\right)$ being the correlation coefficient between the synaptic changes contributed by the *m*th and *n*th spikes of the central neuron, which is particularly non-zero for two central spikes adjacent in time, and

(18) $$\begin{array}{l} f_{m,n;k}=\frac{2\sqrt{{\text{Var}}_{a}\left(\sum_{j}\Delta w_{a,k}\left(t_{m},t_{j}\right)\right)\cdot {\text{Var}}_{a}\left(\sum_{j}\Delta w_{a,k}\left(t_{n},t_{j}\right)\right)}}{\sum_{k}\sum_{i}{\text{Var}}_{a}\left(\sum_{j}\Delta w_{a,k}\left(t_{i},t_{j}\right)\right)} \end{array}$$

being the coupling factors.

Therefore, we can see that there are three factors that are important to understand the efficacy variability Var_*a*_(Δ*w*_*a*_) (Figures 3B–D):

(1) The mean efficacy variability contributed by a single spike of the central neuron, which is represented by *d*.
(2) The correlation between the synaptic changes caused by adjacent spikes of the central neuron, which contributes to Var_*a*_(Δ*w*_*a*_) through *c*_*I*_.
(3) The correlation between the total potentiation and depression value imposed on a synapse, which contributes to Var_*a*_(Δ*w*_*a*_) through *c*_*II*_.

In the following discussions, we will generate spike patterns with different synchronous firing and auto-correlation structure with homogeneous firing rates and cross-correlations using spike-generating models, and investigate how the efficacy variability as well as *d*, *c*_*I*_ and *c*_*II*_ change with model parameters; then we will discuss the physical pictures underlying these phenomena, aiming to giving the readers an intuitive understanding on how synchronous firing and temporal structure influences DiffV.

### 3.2. The influence of auto-correlation structure onto DiffV in stationary spike trains

**Figure 4:**
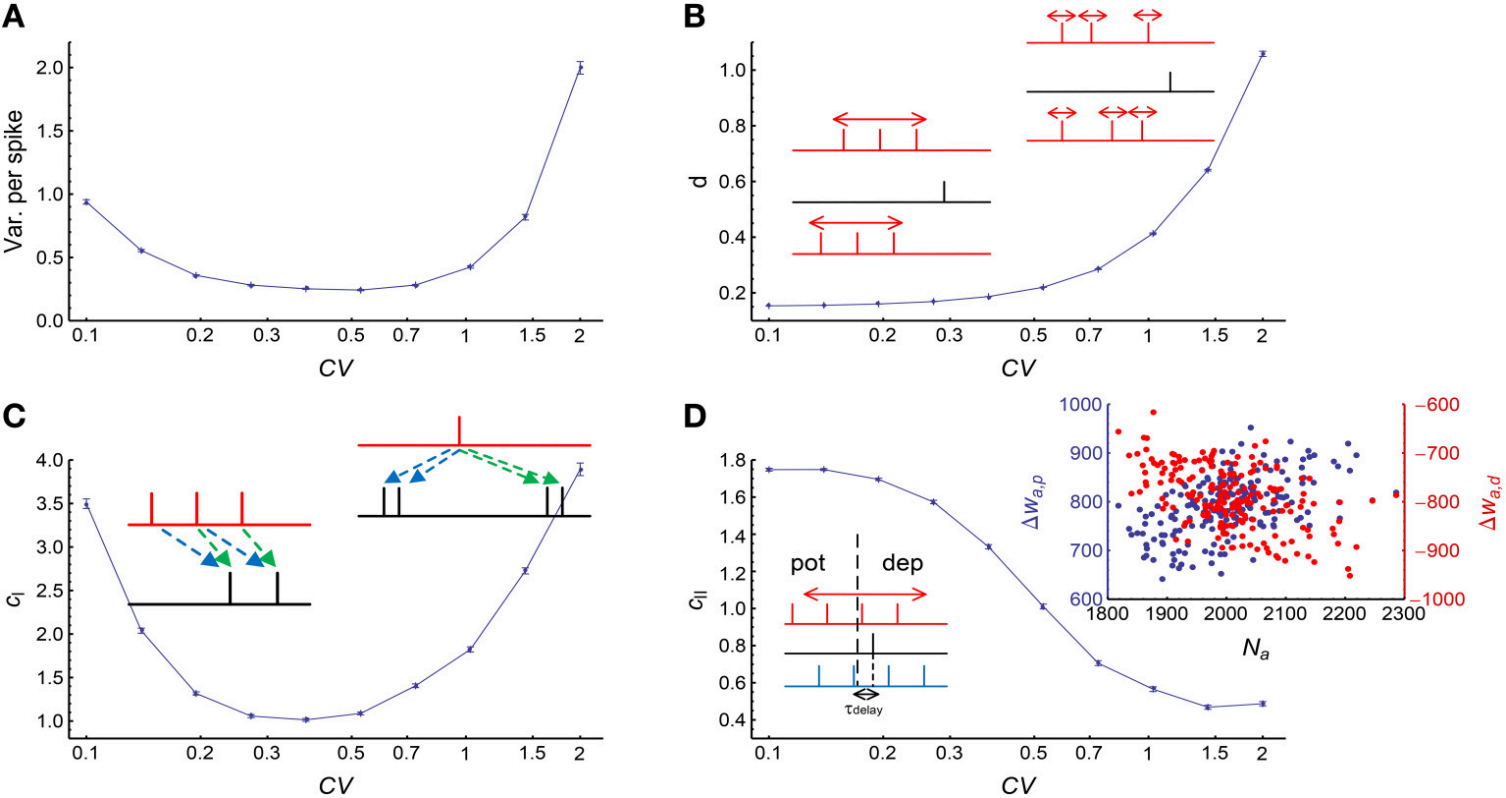
**The influence of auto-correlation structure onto DiffV in stationary spike trains**. **(A)** Variance per spike (${\text{E}}_{T}\left[{\text{Var}}_{a}\left(\Delta w_{a}\right)\right]∕{\bar{N}}_{0}$, see Equation 10) as a function of *CV*, when spike trains are generated according to Model Auto (see Section 2). **(B)** *d* as a function of *CV*. Left inset: when the non-central spike trains are regular, the non-central spikes move left or right almost simultaneously. Right inset: when the non-central spike trains get bursty, each spike can move with a larger freedom, thereby contributing to the increase of DiffV. Black lines in the insets represent the central spike train, and colored (here, red) lines represent the non-central spike trains: the same color scheme is also used in the insets of the following panels. **(C)** *c*_*I*_ as a function of *CV*. Left inset: when the spike trains are regular, the synaptic updatings caused by adjacent central spikes are correlated because of transient cross-correlation. Right inset: when the central spike train gets bursty, the synaptic updatings caused by adjacent spikes in the same bursting events are correlated. Dashed arrows of the same color in the two insets represent similar synaptic updatings during STDP. **(D)** *c*_*II*_ as a function of *CV*. Left inset: when the non-central spike trains are regular, the spikes that potentiate and depress the synapses move left or right almost simultaneously, which correlates the total potentiation and depression values. In this inset, the red spike train cause weaker potentiation and stronger depression than the blue spike train. Right inset: when the spike trains are bursty (here, *CV* = 2), the spike number of a non-central neuron (horizontal coordinate) is positively correlated with the total potentiation (blue) value and negatively correlated with the total depression (red) value. In **(A–D)**, error bars represent s.e.m., the converging motif has 200 non-central neurons. Parameters for STDP: *A*_*d*_ = *A*_*p*_ = 1 (see Figure 2B and Equation 6 in Section 2). All synaptic efficacies were 0 at the beginning, and simulations were run for 100 s biological time, with 32 trials.

We used Gamma processes (Model Auto in Section 2) to model the auto-correlation structure of stationary spike trains (whose firing rates do not change with time). We changed the shape parameter α of the Gamma processes while conserving the firing rates at *r*_0_ = 20Hz. The coefficient of variance (*CV*) of a Gamma process is $CV=1∕\sqrt{\alpha }$. When *CV* gets larger, spike trains are burstier; when *CV* gets smaller, spikes are more regular. We found that both burstiness and strong regularity in auto-correlation structure increased the efficacy variability, and the efficacy variability gets its minimal value when *CV* is around 0.3 ~ 0.7 (Figure 4A), which is the range most neurons lie within *in vivo* (Softky and Koch, 1993).

Spike trains in this model are stationary processes, and we also let *A*_*p*_ = *A*_*d*_ in our model, with *A*_*p*_ and *A*_*d*_ being the strengths of the exponentially decayed STDP windows for potentiation and depression (see Figure 2B). Therefore,

(1) the variance caused by potentiation and depression processes should be equal. From Equation 16, this means that *f*_*PD*_ = 1, so that *c*_*II*_ can link with ρ_*PD*_ in a direct way:
  (19) $$\begin{array}{l} c_{II}=1+\rho_{PD} \end{array}$$
(2) ${\text{Var}}_{a}\left(\underset{j}{\sum }\Delta w_{a,k}\left(t_{i},t_{j}\right)\right)$ does not change with the *i* index. From Equations 17 and 18, this means that *c*_*I*_ can link with ρ_*m, n*; *k*_ in a direct way:
  (20) $$\begin{array}{l} c_{I}=1+\frac{\sum_{k}\sum_{m<n}\rho_{m,n;k}}{N_{0}} \end{array}$$

with *N*_0_ being the spike number of the central neuron.

Equations 19 and 20 mean that in this model the change of *c*_*I*_ and *c*_*II*_ with model parameters directly reflect the change of the correlations.

To understand Figure 4A, we investigated how *d*, *c*_*I*_ and *c*_*II*_ change with *CV* (Figures 4B–D). We found that *d* monotonically increases with *CV* (Figure 4B), *c*_*I*_ gets its minimum when *CV* is around 0.3 ~ 0.7, and gets large both when *CV* is too large or too small (Figure 4C), and *c*_*II*_ monotically decreases with *CV* (Figure 4D). With the help of Equations 19 and 20, we can understand their changes with *CV*, and thus gain insight on the reason for the change of DiffV with *CV*.

#### 3.2.1. Understanding the change of *d* with *CV*

To understand the change of *d* with *CV*, consider the term ${\text{Var}}_{a}\left(\underset{j}{\sum }\Delta w_{a,p}\left(t_{i},t_{j|a,i,p}\right)\right)$ in Equation 13. When the spike trains of the non-central neurons are regular, *t*_*j*|*a, i, p*_ ≈ *t*_1|*a, i, p*_ − (*j* − 1)∕*r*_0_, with *r*_0_ being the firing rate, so the difference of $\underset{j}{\sum }\Delta w_{a,p}\left(t_{i},t_{j|a,i,p}\right)$ for different *a*s mainly comes from the difference of *t*_1|*a, i, p*_ (Figure 4B, left inset). What's more, {_*t*_1|*a, i, p*_}*a*_ approaches to a uniform distribution within the interval (*t*_*i*_ − τ_*delay*_ − 1∕*r*_0_, *t*_*i*_ − τ_*delay*_) when the spike trains get regular. When the spike trains get burstier, not only the distribution of *t*_1|*a, i, p*_ gets broader, but the other spikes *t*_*j*≠1|*a, i, p*_ can also move with a larger freedom, which increases the variability of $\underset{j}{\sum }\Delta w_{a,p}\left(t_{i},t_{j|a,i,p}\right)$ (Figure 4B, right inset). Similar arguments also apply to the *k* = *d* case. It is easy to show (Supplementary Material Section S1.2) that under strict regularity (*CV* = 0),

(21) $$\begin{array}{l} d_{reg}=\left(A_{d}^{2}+A_{p}^{2}\right)\frac{\tau_{STDP}}{2\Delta t}\left[\frac{1+\exp\left(-\frac{\Delta t}{\tau_{STDP}}\right)}{1-\exp\left(-\frac{\Delta t}{\tau_{STDP}}\right)}-\frac{2\tau_{STDP}}{\Delta t}\right], \end{array}$$

with Δ*t* = 1∕*r*_0_ being the inter-spike interval; and under Poisson process (*CV* = 1),

(22) $$\begin{array}{l} d_{Poi}=\left(A_{d}^{2}+A_{p}^{2}\right)\frac{\tau_{STDP}}{2\Delta t}. \end{array}$$

As $\frac{1+\exp\left(-\frac{\Delta t}{\tau_{STDP}}\right)}{1-\exp\left(-\frac{\Delta t}{\tau_{STDP}}\right)}-\frac{2\tau_{STDP}}{\Delta t}<1$ (Supplementary Material Section S1.2), *d*_*reg*_ < *d*_*Poi*_.

#### 3.2.2. Understanding the change of *c*_*I*_ with *CV*

*c*_*I*_ does not change monotonically with *CV*, it is large both when *CV* is large (i.e., spike trains are bursty) and when *CV* is very small (i.e., spike trains are very regular), and gets its minimal value when *CV* is around 0.3 ~ 0.7 (Figure 4B). Now we try to understand the change of *c*_*I*_ with *CV*.

Suppose there are two spikes of the central neuron at time *x* and *y*. Because the spike trains in this model are stationary processes, the correlation between the potentiation (or depression) value caused by these two spikes only depends on *y* − *x*:

(23) $$\begin{array}{rcl} \rho_{p}\left(y-x\right) & = & Corr_{a}\left(\underset{j\in \left(x,\infty \right)}{\sum }\Delta w_{a,p}\left(x,j\right),\underset{j\in \left(y,\infty \right)}{\sum }\Delta w_{a,p}\left(y,j\right)\right) \end{array}$$

(24) $$\begin{array}{rcl} \rho_{d}\left(y-x\right) & = & Corr_{a}\left(\underset{j\in \left(-\infty ,x\right)}{\sum }\Delta w_{a,d}\left(x,j\right),\underset{j\in \left(-\infty ,y\right)}{\sum }\Delta w_{a,d}\left(y,j\right)\right) \end{array}$$

with Δ*w*_*a, k*_(*x, j*) being the synaptic updating caused by the interaction of *x* and a spike of the *a*th non-central neuron at time *j*. This ρ_*k*_(*l*) function depends on the *CV* of the spike trains of the non-central neurons. Because of the time-reversal invariance of the statistics of the spike trains, ρ_*p*_(*l*) = ρ_*d*_(*l*)≡ρ(*l*) in our model. Using ρ(*l*), *c*_*I*_ (Equation 20) can be rewritten as

(25) $$\begin{array}{l} c_{I}=1+\frac{2}{r_{0}}\int_{0}^{\infty }C_{0}\left(l\right)\rho \left(l\right)\text{d}l \end{array}$$

with *r*_0_ being the firing rate of the central neuron, and *C*_0_(*l*) being the auto-correlation of the central neuron. $\frac{C_{0}\left(l\right)}{r_{0}}$ means the probability density to find another central spike at time *l* given an central spike at time 0, which can be written as

(26) $$\begin{array}{l} \frac{C_{0}\left(l\right)}{r_{0}}=\overset{\infty }{\underset{i=1}{\sum }}q_{i}\left(l\right) \end{array}$$

with *q*_*i*_(*l*) being the distribution of the interval between the spike at time 0 and the next *i*th spike. Spike trains in our model are Gamma processes, so that the inter-spike interval follows $\Gamma \left(x|\alpha ,\beta \right)=\frac{1}{\Gamma \left(\alpha \right)\beta^{\alpha }}x^{\alpha -1}e^{-x∕\beta }$. It is easy to show that *q*_*i*_(*l*) = Γ(*l*|*iα*, β) (http://mathworld.wolfram.com/GammaDistribution.html).

From Equation 25, we know that the *CV* of the non-central spike trains influences *c*_*I*_ through ρ(*l*), while the *CV* of the central spike train influences *c*_*I*_ through *q*_0_(*l*).

**Figure 5:**
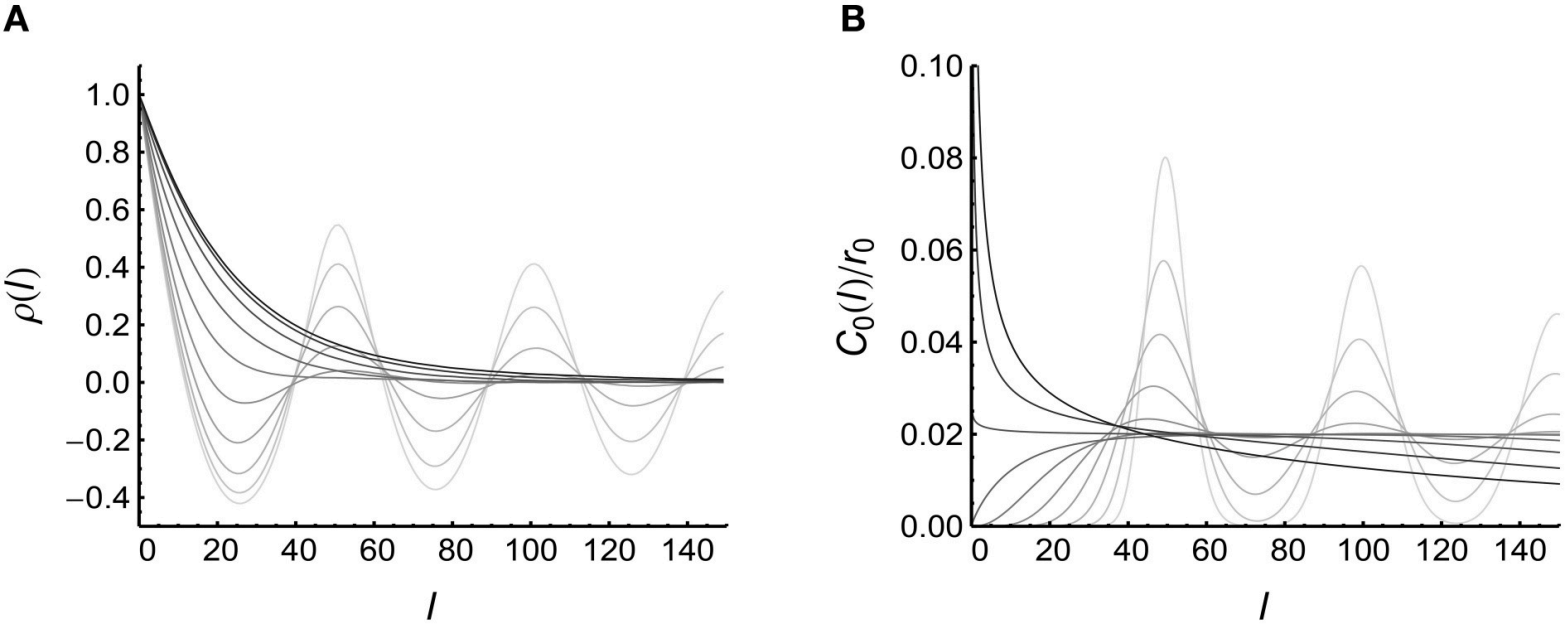
**Understanding the influence of auto-correlation structure onto *c*_*I*_ (see Equation 25)**. **(A)** ρ(*l*) when *CV* = 0.1, 0.139, 0.195, 0.271, 0.379, 0.528, 0.737, 1.03, 1.43, 2.00 (represented by lines from light to dark). **(B)** *C*_0_(*l*)∕*r*_0_ when *CV* takes values the same as in **(A)**. In **(A)**, the results are from 10,000 trials of simulations on a converging motif with 200 non-central neurons. **(B)** is plotted using Equation 26.

To understand the change of *c*_*I*_ with *CV*, we plot the functions ρ(*l*) and *C*_0_(*l*)∕*r*_0_ under different *CV* values (Figure 5). We find that:

(1) When spike trains are bursty (*CV* > 1), ρ(*l*) increases with *CV*, and *C*_0_(*l*)∕*r*_0_ becomes more concentrated near zero (at which point ρ(*l*) gets its maximal value) when *CV* increases. Both of these two factors increase the integration $\frac{1}{r_{0}}\int C_{0}\left(l\right)\rho \left(l\right)\text{d}l$ in Equation 25, contributing to the increase of *c*_*I*_ with *CV* when spike trains are bursty.
(2) When spike trains are very regular (*CV* is very small), both ρ(*l*) and *C*_0_(*l*)∕*r*_0_ become more concentrated near *l* = *N*∕*r*_0_ (with *r*_0_ = 20 Hz being the firing rate and *N* being an positive integer): in this case, $\frac{1}{r_{0}}\int C_{0}\left(l\right)\rho \left(l\right)\text{d}l$ also increases because of the overlap of the peaks of ρ(*l*) and *C*_0_(*l*)∕*r*_0_.

Intuitively, the change of *c*_*I*_ with *CV* can be understood like this:

(1) When *CV* is large, the non-central spike trains tend to be clustered into bursting events, and the time scale τ_*auto*_ of the bursting events increases with *CV* (it is easy to show that $\tau_{auto}\sim \frac{CV^{2}}{r_{0}}$ when *CV* > 1, see Supplementary Material Section S1.1): this strengthened and broadened auto-correlation makes ρ(*l*) increase with *CV* when *CV* > 1. At the same time, more adjacent central spikes are also gathered closer by bursting events, which increases the correlation between the synaptic changes caused by adjacent central spikes (Figure 4C, right inset): this is the effect of *C*_0_(*l*)∕*r*_0_.
(2) When *CV* is very small, the spike trains are very regular. In this case, two adjacent spikes {*t*_*i*_, *t*_*i*+*m*_} of the central neuron have the relation *t*_*i*+*m*_ ≈ *t*_*i*_+*m*∕*r*_0_ if they are well within the time scale τ_*auto*_ of the oscillating decaying auto-correlation (it is easy to show that $\tau_{auto}\sim \frac{1}{4CV^{2}r_{0}}$ when *CV* < 1, see Supplementary Material Section S1.1); and two spikes {*t*_*j*|*a, i, k*_, *t*_*j*|*a, i*+*m, k*_} of the *a*th non-central neuron also have the relation *t*_*j*|*a, i*+*m, k*_ ≈ *t*_*j*|*a, i, k*_+*m*∕*r*_0_. This means that *t*_*i*_−*t*_*j*|*a, i, k*_ ≈ *t*_*i*+*m*_−*t*_*j*|*a, i*+*m, k*_, which makes the synaptic changes caused by *t*_*i*_ and *t*_*i*+*m*_ almost the same (i.e., $\underset{j}{\sum }\Delta w_{a,k}\left(t_{i},t_{j}\right)\approx \underset{j}{\sum }\Delta w_{a,k}\left(t_{i+m},t_{j}\right)$) (Figure 4C, left inset). This increases the correlation ρ_*i, i*+*m*; *k*_ caused by adjacent central spikes, thereby increasing *c*_*I*_.

In this model (Model Auto in Section 2), we suppose that the firing rates of the non-central neurons r_*_ are the same as the firing rate of the central neuron *r*_0_. What if r_*_ ≠ *r*_0_? Figure 5 suggests that when spike trains are very regular, ρ(*l*) peaks at *N*∕r_*_ (with *N* being a positive integer), and *C*_0_(*l*)∕*r*_0_ peaks at *N*∕*r*_0_ when *CV* ≪ 1. Equation 25 indicates that *c*_*I*_ gets its maximum value when the peaks of ρ(*l*) overlap with the peaks of *C*_0_(*l*)∕*r*_0_. This overlap is strong when r_*_ = *r*_0_, but can also occur when *N*_1_r_*_ = *N*_2_*r*_0_, with *N*_1_ and *N*_2_ being two positive integers with no common divisor larger than 1. For the simplicity of the following argument, we call the increase of the correlation ρ_*m, n*; *k*_ caused by adjacent central spikes under strong regularity and *N*_1_r_*_ = *N*_2_*r*_0_ to be the mechanism of *transient cross-correlation*. Because of the decaying oscillation of ρ(*l*) and *C*_0_(*l*)∕*r*_0_, *N*_1_ and *N*_2_ tend to be small integers.

#### 3.2.3. Understanding the change of *c*_*II*_ with *CV*

We find that *c*_*II*_ > 1 when spike trains are regular, which means that the potentiation and depression values are positively correlated (ρ_*PD*_ > 0) in this case; and *c*_*II*_ < 1 when spike trains are bursty, which means that the potentiation and depression values are negatively correlated (ρ_*PD*_ < 0). Now we try to understand the change of *c*_*II*_ with *CV*.

(1) Under strong regularity, if *t*_*j*|*a, i, k*_ are well within the time scale of τ_*auto*_ with *t*_1|*a, i, p*_, then *t*_*j*|*a, i, p*_ ≈ *t*_1|*a, i, p*_ − (*j* − 1)∕*r*_0_ and *t*_*j*|*a, i, d*_ ≈ *t*_1|*a, i, p*_+*j*∕*r*_0_. This means that if *t*_1|*a, i, p*_ moves farther away from *t*_*i*_, the potentiation value $\underset{j}{\sum }\Delta w_{a,p}\left(t_{i},t_{j|a,i,p}\right)$ will get weakened, while the depression value $\underset{j}{\sum }\Delta w_{a,d}\left(t_{i},t_{j|a,i,d}\right)$ will get strengthened (Figure 4D, left inset), which means that $\underset{j}{\sum }\Delta w_{a,p}\left(t_{i},t_{j|a,i,p}\right)$ and $\underset{j}{\sum }\Delta w_{a,d}\left(t_{i},t_{j|a,i,d}\right)$ positively correlated. This makes $\underset{i}{\sum }\underset{j}{\sum }\Delta w_{a,p}\left(t_{i},t_{j|a,i,p}\right)$ and $\underset{i}{\sum }\underset{j}{\sum }\Delta w_{a,d}\left(t_{i},t_{j|a,i,d}\right)$ are also positively correlated. By the definition of ρ_*PD*_, we know that ρ_*PD*_ > 0 in this case.
(2) To understand the case when *CV* is large, note that the Fano factor of a long time scale approaches to *CV*^2^ (Cox, 1962; Tuckwell, 1988; Nawrot et al., 2008), which means that different non-central neurons may emit quite different numbers of spikes during simulation when *CV* is large. If the *a*th non-central neuron fires more (less) spikes, then both the potentiation and depression values imposed on the *a*th synapses tend to be strong (weak) (Figure 4D, right inset). This is the reason why ρ_*PD*_ is negative when spike trains are bursty.

Comparing Figure 4A with Figures 4B–D, we know that both *d* and *c*_*I*_ are the reasons for the steep increase of DiffV under large *CV*, while *c*_*I*_ (i.e., the mechanism of transient cross-correlation) is the reason for the fast increase of DiffV with the decrease of *CV* under small *CV*. *c*_*II*_, however, does not significantly contribute to the change of DiffV under large or small *CV*.

#### 3.2.4. The interaction of auto-correlation structure with heterogeneity of rates

Till now, we only considered the case when the spike trains are Gamma processes with the same firing rate. In Supplementary Material Section S2, we will consider the case that the Gamma processes of different non-central neurons have different firing rates, while their mean firing rate is still the same as the firing rate of the central neuron *r*_0_. Our simulations suggest that heterogeneity of rates does not strongly influence DiffV (as different synapses have different diffusion strengths under heterogeneity of rates, DiffV here means the mean diffusion strength over all the synapses) when the spike trains are bursty, but discounts the increase of DiffV with the decrease of *CV* under strong regularity because of it destroying transient cross-correlation (Supplementary Figure 1). This makes DiffV tend to monotonically increase with *CV*.

### 3.3. The influence of synchronous firing onto DiffV

There are usually two scenarios regarding to synchronous firing: one is spike synchrony, which means that the spikes of neurons are emitted almost simultaneously; the other one is rate synchrony, which means that the firing rates of neurons rise and fall at the same time. The key difference between these two scenarios is the distribution of the spike number of a neuron within a synchronous event. For spike synchrony, a neuron can fire no more than a single spike in a synchronous event, so that this distribution is narrow; but for rate synchrony, different neurons can fire a different number of spikes in a synchronous event, so that this distribution is broad. To understand how DiffV is influenced by synchronous firing under different distributions of spike numbers per neuron per synchronous event, we generated spike trains using Model Sync (Section 2). In this model, a synchronous event lasts for τ_*cross*_. The spike number per neuron per synchronous event is distributed the same as the spike number of an underlying Gamma process within an interval of length τ_*cross*_. The rate of this Gamma process is *p*∕τ_*cross*_ and its coefficient of variance is *CV*_*SpikeNum*_. So the mean value of the distribution of spike number per neuron per synchronous event is *p*, and this distribution is narrow if *CV*_*SpikeNum*_ is small, and gets broadened when *CV*_*SpikeNum*_ increases. If a neuron is to fire *M* spikes in a synchronous event, then the spike times of these *M* spikes will be randomly and uniformly chosen within this time interval of duration τ_*cross*_. The occurrence of synchronous events is a Poisson process with rate *r*_0_∕*p*, so that the firing rate of a neuron is kept at *r*_0_ = 20Hz when *p* changes. We focus on the case that τ_*cross*_ ≪ τ_*STDP*_ and τ_*cross*_ ≪ *p*∕*r*_0_ (with *p*∕*r*_0_ being the average inter-event interval) in our study. It is easy to think that if τ_*cross*_ ≪ *p*∕*r*_0_ but τ_*cross*_ > τ_*STDP*_, STDP updatings will tend to zero so that DiffV will decrease with τ_*cross*_; and if τ_*cross*_ > *p*∕*r*_0_, the spike pattern will approach to asynchronous state.

For spike patterns with synchronous firing, we don't have simple equations like Equations 19 and 20 to directly link ρ_*m, n*; *k*_ and ρ_*PD*_ with *c*_*I*_ and *c*_*II*_. The coupling factors *f*_*PD*_ (Equation 16) and *f*_*m, n*; *k*_ (Equation 18) must be considered. As

$$\begin{array}{l} \sqrt{{\text{Var}}_{a}\left(\underset{j}{\sum }\Delta w_{a,k}\left(t_{m},t_{j}\right)\right)\cdot {\text{Var}}_{a}\left(\underset{j}{\sum }\Delta w_{a,k}\left(t_{n},t_{j}\right)\right)}\;\\ \;\leq \frac{1}{2}\left({\text{Var}}_{a}\left(\underset{j}{\sum }\Delta w_{a,k}\left(t_{m},t_{j}\right)\right)+{\text{Var}}_{a}\left(\underset{j}{\sum }\Delta w_{a,k}\left(t_{n},t_{j}\right)\right)\right) \end{array}$$

and the equality is obtained only when ${\text{Var}}_{a}\left(\underset{j}{\sum }\Delta w_{a,k}\left(t_{m},t_{j}\right)\right)={\text{Var}}_{a}\left(\underset{j}{\sum }\Delta w_{a,k}\left(t_{n},t_{j}\right)\right)$, *f*_*m, n*; *k*_ reflects the inhomogeneity of ${\text{Var}}_{a}\left(\underset{j}{\sum }\Delta w_{a,k}\left(t_{m},t_{j}\right)\right)$ for different *m*. And similarly, *f*_*PD*_ reflects the difference between ${\text{Var}}_{a}\left(\underset{i}{\sum }\underset{j}{\sum }\Delta w_{a,p}\left(t_{i},t_{j}\right)\right)$ and ${\text{Var}}_{a}\left(\underset{i}{\sum }\underset{j}{\sum }\Delta w_{a,d}\left(t_{i},t_{j}\right)\right)$ relative to their total values.

**Figure 6:**
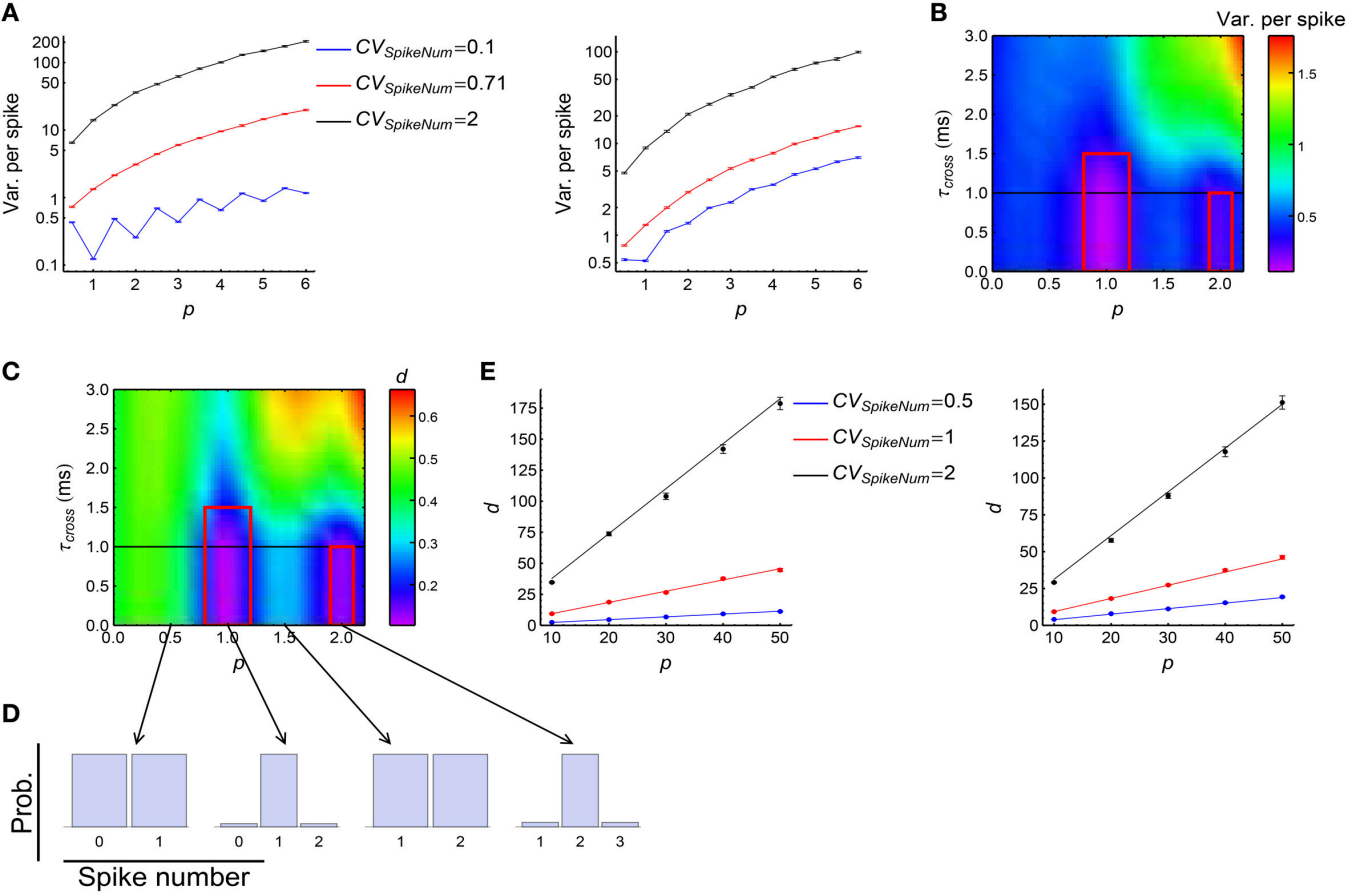
**The influence of synchronous firing onto DiffV and the contribution of *d***. **(A)** Variance per spike (${\text{E}}_{T}\left[{\text{Var}}_{a}\left(\Delta w_{a}\right)\right]∕{\bar{N}}_{0}$, see Equation 10) as a function of *p* under different *CV*_*SpikeNum*_ values for τ_*cross*_ = 0.5 ms (left) and τ_*cross*_ = 2.5 ms (right), when spike trains are generated according to Model Sync (see Section 2). The efficacy variances span a large range, so here we use log scale to better show their changes. **(B)** Variance per spike as a function of *p* and τ_*cross*_ when *CV*_*SpikeNum*_ = 0.1. Note the reduction of variance per spike in the red squares. The black horizontal line represents the axonal delay τ_*delay*_ = 1 ms. **(C)** *d* as a function of *p* and τ_*cross*_ when *CV*_*SpikeNum*_ = 0.1. Note the reduction of *d* in the red squares. **(D)** The distribution of spike number per neuron per synchronous event under different *p* values indicated by the starting points of the arrows, when *CV*_*SpikeNum*_ = 0.1. Note that the distribution peaks at *p* when *p* is an integer. **(E)** Comparison of the numeric results (dots with error bars) of *d* with the analytic results (solid lines) given by Equations 28 and 29 for τ_*cross*_ = 0.5 ms (left) and τ_*cross*_ = 2.5ms (right). In **(A–E)**, the size of the converging motif, the parameters for STDP as well as the simulation time and trials are the same as in Figure 4. Error bars represent s.e.m.

We investigated how DiffV changes with *p*, τ_*cross*_ and the *CV*_*SpikeNum*_. Our simulations suggest:

(1) DiffV tends to increase with the strength of synchronous firing (i.e., *p*) and the broadness of the distribution of the spike number per neuron per synchronous event (i.e., *CV*_*SpikeNum*_) (Figure 6A, see Supplementary Figures 2A1,B1,C1 for more information);
(2) when *CV*_*SpikeNum*_ is small enough, DiffV can be significantly decreased if *p* is close to an integer and τ_*cross*_ < τ_*delay*_ (the red squares in Figure 6B).

**Figure 7:**
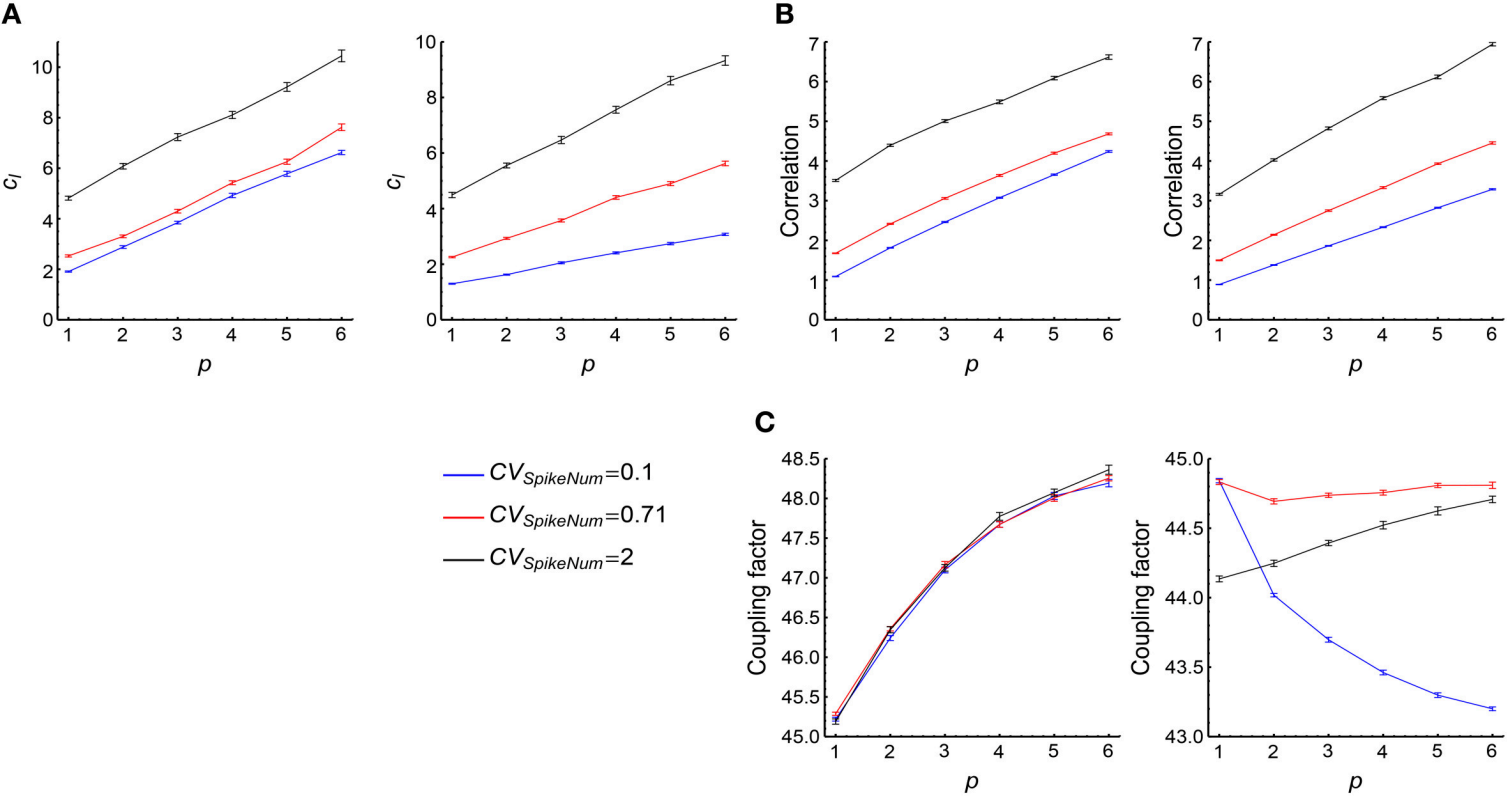
**The contribution of *c*_*I*_ to the influence of synchronous firing onto DiffV**. **(A)** *c*_*I*_ as a function of *p* under different *CV*_*SpikeNum*_ values for τ_*cross*_ = 0.5 ms (left) and τ_*cross*_ = 2.5 ms (right), when spike trains are generated according to Model Sync (see Section 2). **(B)** $\underset{l,k}{\sum }\rho_{l,k}$ as a function of *p* under different *CV*_*SpikeNum*_ for τ_*cross*_ = 0.5 ms (left) and τ_*cross*_ = 2.5 ms (right). We found that ρ_*l, k*_ safely decays to zero when *l* ≥ 50 in our parameter range, therefore we cut off *l* at *l* = 50 when calculating the summation. **(C)** $\underset{l,k}{\sum }f_{l,k}$ as a function of *p* for τ_*cross*_ = 0.5 ms (left) and τ_*cross*_ = 2.5 ms (right). Note that the percentage that $\underset{l,k}{\sum }f_{l,k}$ changes with *p* or *CV*_*SpikeNum*_ is far smaller than that of $\underset{l,k}{\sum }\rho_{l,k}$. In **(A–C)**, the size of the converging motif, the parameters for STDP as well as the simulation time and trials are the same as in Figure 4. Error bars represent s.e.m.

Here we explain the underlying physical pictures of these phenomena based on the observation of the change of *d*, *c*_*I*_ and *c*_*II*_ with model parameters:

(1) *d* is the only reason for the second phenomenon above (the red squares in Figure 6C, compare with the changes of *c*_*I*_ and *c*_*II*_ in Supplementary Figures 2A3,A4). To understand this, suppose a synchronous event happening during [*t*_1_, *t*_2_] (*t*_2_ = *t*_1_ + τ_*cross*_), then the central neuron will receive its afferents during [*t*_1_ + τ_*delay*_, *t*_2_ + τ_*delay*_], with τ_*delay*_ being the axonal delay. If τ_*cross*_ ≤ τ_*delay*_, the spike time *t*_0_ of the central neuron *t*_0_ < *t*_1_ + τ_*delay*_, which means that the central neuron always starts to receive spikes after its own firing in a synchronous event, so that all the spikes it receives depress the corresponding synapses after the synchronous event. If τ_*cross*_ ≤ τ_*delay*_ ≪ τ_*STDP*_, the depression value Δ*w* contributed by each non-central spike is approximately the same. If *p* is an integer and *CV*_*SpikeNum*_ is small enough, the distribution of the spike number per neuron per synchronous event will sharply peak at *p* (Figure 6D). In this case, nearly all the non-central neurons fire exactly *p* spikes during a synchronous event, so that almost all the synapses are depressed by *pΔw* after this synchronous event. This is the reason why *d* is so small in this case. Mathematically, it can be shown (Supplementary Materials Section S3.1) that when τ_*cross*_ ≤ τ_*delay*_ ≪ τ_*STDP*_,
  (27) $$\begin{array}{l} d\approx {\text{Var}}_{a}\left(N_{a}\right)\left[A_{d}^{2}\exp\left(-\frac{2\tau_{delay}}{\tau_{STDP}}\right)+\frac{r_{0}\tau_{STDP}}{2p}\left(A_{d}^{2}+A_{p}^{2}\right)\right] \end{array}$$
  with E_*T*_ represent average over trials, and Var_*a*_(*N*_*a*_) being the variance of spike numbers of the non-central neurons in a synchronous event. We can see that *d* gets reduced if Var_*a*_(*N*_*a*_) is small, which is the case when *CV*_*SpikeNum*_ is small and *p* is an integer. When τ_*cross*_ > τ_*delay*_, however, it is possible that *t*_1_ + τ_*delay*_ < *t*_0_ < *t*_2_ + τ_*delay*_, so that all the in-coming spikes of the central neuron during [*t*_1_ + τ_*delay*_, *t*_0_) potentiate the corresponding synapses, and all the in-coming spikes during (*t*_0_, *t*_2_ + τ_*delay*_] depress the corresponding synapses. This may split the synapses into different directions, thereby increasing *d* (note the large *d* value when τ_*cross*_ > τ_*delay*_ in Figure 6C).
(2) *d* tends to increase with *p* or *CV*_*SpikeNum*_ (Figure 6E, see Supplementary Figures 2A2,B2,C2 for more information), thereby contributing to the tendency of the increase of DiffV with *p* or *CV*_*SpikeNum*_. In this paper, we generally suppose that τ_*cross*_ ≪ τ_*STDP*_ and τ_*delay*_ ≪ τ_*STDP*_. When *p* large, it can be shown (Supplementary Materials Section S3.1) that if τ_*cross*_ ≤ τ_*delay*_, then
  (28) $$\begin{array}{r} d=E_{T}\left[{\text{Var}}_{a}\left(\underset{j}{\sum }\Delta w_{a,d}\left(t_{i},t_{j}\right)\right)\right]+{\text{E}}_{T}\left[{\text{Var}}_{a}\left(\underset{j}{\sum }\Delta w_{a,p}\left(t_{i},t_{j}\right)\right)\right]\\ \;\approx pCV_{SpikeNum}^{2}A_{d}^{2}\exp\left(-\frac{2\tau_{delay}}{\tau_{STDP}}\right)\\ \;+\;CV_{SpikeNum}^{2}\frac{r_{0}\tau_{STDP}}{2}\left(A_{d}^{2}+A_{p}^{2}\right), \end{array}$$
  with E_*T*_ representing trial average; and if τ_*cross*_ > τ_*delay*_, then
  (29) $$\begin{array}{l} d\approx p[CV_{SpikeNum}^{2}A_{d}^{2}\frac{\tau_{delay}}{\tau_{cross}}\exp(-\frac{\tau_{cross}+\tau_{delay}}{\tau_{STDP}})\\ \;+\;A_{d}^{2}\exp(-\frac{\tau_{cross}+\tau_{delay}}{2\tau_{STDP}})\cdot A\\ \;+\;A_{p}^{2}\exp(-\frac{\tau_{cross}-\tau_{delay}}{2\tau_{STDP}})\cdot B]\\ \;+\;CV_{SpikeNum}^{2}\frac{r_{0}\tau_{STDP}}{2}(A_{d}^{2}+A_{p}^{2}) \end{array}$$
  with
  $$A=\left(\frac{1}{6}+\frac{1}{3}CV_{SpikeNum}^{2}\right)-\frac{1}{2}{\left(\frac{\tau_{delay}}{\tau_{cross}}\right)}^{2}-\frac{1}{3}\left(CV_{SpikeNum}^{2}-1\right){\left(\frac{\tau_{delay}}{\tau_{cross}}\right)}^{3}$$
  $$B={\left(1-\frac{\tau_{delay}}{\tau_{cross}}\right)}^{2}\left(\frac{1}{2}+\frac{1}{3}\left(CV_{SpikeNum}^{2}-1\right)\left(1-\frac{\tau_{delay}}{\tau_{cross}}\right)\right).$$
  We compare the analytic results with numeric results in Figure 6E, we can see the tendency that *d* increases with both *p* and *CV*_*SpikeNum*_. The first terms of Equations 28 and 29 represent the contribution of the synchronous event S_0_ that the central spike *t*_*i*_ belongs to, and the last terms represent the contributions of the other synchronous events. *p* only exists in the first terms of Equations 28 and 29, which suggests that *p* increases *d* through increasing the synchrony strength of S_0_. *CV*_*SpikeNum*_ exists in both terms, which suggests that the broadness of the spike number distribution in both S_0_ and the other synchronous events contribute to *d*.
(3) *c*_*I*_ tends to increase with both *p* and *CV*_*SpikeNum*_ (Figure 7A, see more in Supplementary Figures 2A3, B3, C3), thereby contributing to the tendency of the increase of DiffV with *p* or *CV*_*SpikeNum*_. From Equation 17, we know that the change of *c*_*I*_ may either caused by the correlation ρ_*m, n*; *k*_ or by the coupling factor *f*_*m, n*; *k*_. To estimate their contributions to *c*_*I*_, we define
  (30) $$\begin{array}{l} \rho_{l,k}=\frac{\sum_{m}\rho_{m,\left(m+l\right);k}}{\sum_{m}1},\;l=1,2,\cdots \;;k=p,d, \end{array}$$
  and
  (31) $$\begin{array}{l} f_{l,k}=\underset{m}{\sum }f_{m,\left(m+l\right);k}\;l=1,2,\cdots \;;k=p,d. \end{array}$$
  We find that the change of $\underset{k}{\sum }\underset{l}{\sum }f_{l,k}$ with model parameters is far slower than the change of $\underset{k}{\sum }\underset{l}{\sum }\rho_{l,k}$ (Figures 7B,C), which suggests that the coupling factors *f*_*m, n*; *k*_ do not significantly contribute to the change of *c*_*I*_, and the correlation ρ_*m, n*; *k*_ between synaptic updatings caused by adjacent central spikes is the main reason for the change of *c*_*I*_. It is easy to think that if two spikes of the central neuron are in the same synchronous event, then the correlation of the synaptic updatings caused by them tends to be large; but if they are in different synchronous events, then the correlation of the synaptic changes caused by them tends to be small. To understand the increase of *c*_*I*_ with *p* and *CV*_*SpikeNum*_, let us consider the following toy model. Suppose that ρ_*mn*_ = *a*_0_ if *m* and *n* are in the same synchronous event (this assumption is particularly correct when τ_*cross*_ < τ_*delay*_), and ρ_*mn*_ = 0 if they are in different synchronous events. Suppose that the central neuron fires *N*_*s*_ spikes in the *s*th synchronous event, then the total value of the correlation coefficients will be
  $$\begin{array}{l} \underset{m<n}{\sum }\rho_{mn}=a_{0}\underset{s}{\sum }\frac{N_{s}\left(N_{s}-1\right)}{2}\\ =a_{0}M\int_{-\infty }^{\infty }q\left(N_{s}\right)\frac{N_{s}\left(N_{s}-1\right)}{2}\text{d}N_{s} \end{array}$$
  with *M* being the number of synchronous events, and *q*(*N*_*s*_) being the distribution of the spike number per synchronous event. If *p* is sufficiently large, $q\left(N_{s}\right)\sim N\left(p,\sigma^{2}\right)$ with $\sigma^{2}=p\cdot CV_{SpikeNum}^{2}$. In this case
  (32) $$\begin{array}{r} \underset{m<n}{\sum }\rho_{mn}\approx \frac{a_{0}Mp\left(-1+p+CV_{SpikeNum}^{2}\right)}{2}\\ =\frac{a_{0}N_{0}\left(-1+p+CV_{SpikeNum}^{2}\right)}{2} \end{array}$$
  with *N*_0_ being the total spike number of the central neuron. From Equation 32, we can see that $\underset{m<n}{\sum }\rho_{mn}$ tend to increase with both *p* and *CV*_*SpikeNum*_, which explains why *c*_*I*_ tend to increase with *p* and *CV*_*SpikeNum*_. Intuitively, *p* gathers more spikes into the same synchronous event; and *CV*_*SpikeNum*_, which induces heterogeneity of spike numbers in a synchronous event, concentrates spikes into fewer synchronous events. Both of them increase the number of spike pairs that lie in the same synchronous event, thereby increasing $\underset{m<n}{\sum }\rho_{mn}$.
(4) We found that when *p* is large, the change of *c*_*II*_ with *p* is small (Supplementary Figures 2A4, B4, C4), which suggests that the contribution of *c*_*II*_ to the increase of DiffV with *p* is small compared to the other factors. The change of *c*_*II*_ with *CV*_*SpikeNum*_ is not strong when τ_*cross*_ ≤ τ_*delay*_; and *c*_*II*_ tends to decrease with *CV*_*SpikeNum*_ when τ_*cross*_ > τ_*delay*_ (Supplementary Figures 2A4, B4, C4), which negatively contributes to the increase of DiffV with *CV*_*SpikeNum*_. These suggest that *c*_*II*_ is not an important factor to understand the change of DiffV under synchronous firing. We move more discussions on *c*_*II*_ into Supplementary Materials Section S3.2.

From the discussion above, we know that both the mean (controlled by *p*) and variance (controlled by *CV*_*SpikeNum*_) of the spike number per neuron per synchronous event are important factors to understand the change of DiffV under synchronous firing. They contribute to DiffV mainly through *d* and *c*_*I*_.

### 3.4. The influence of the interaction of auto-correlation structure and synchronous firing onto DiffV

**Figure 8:**
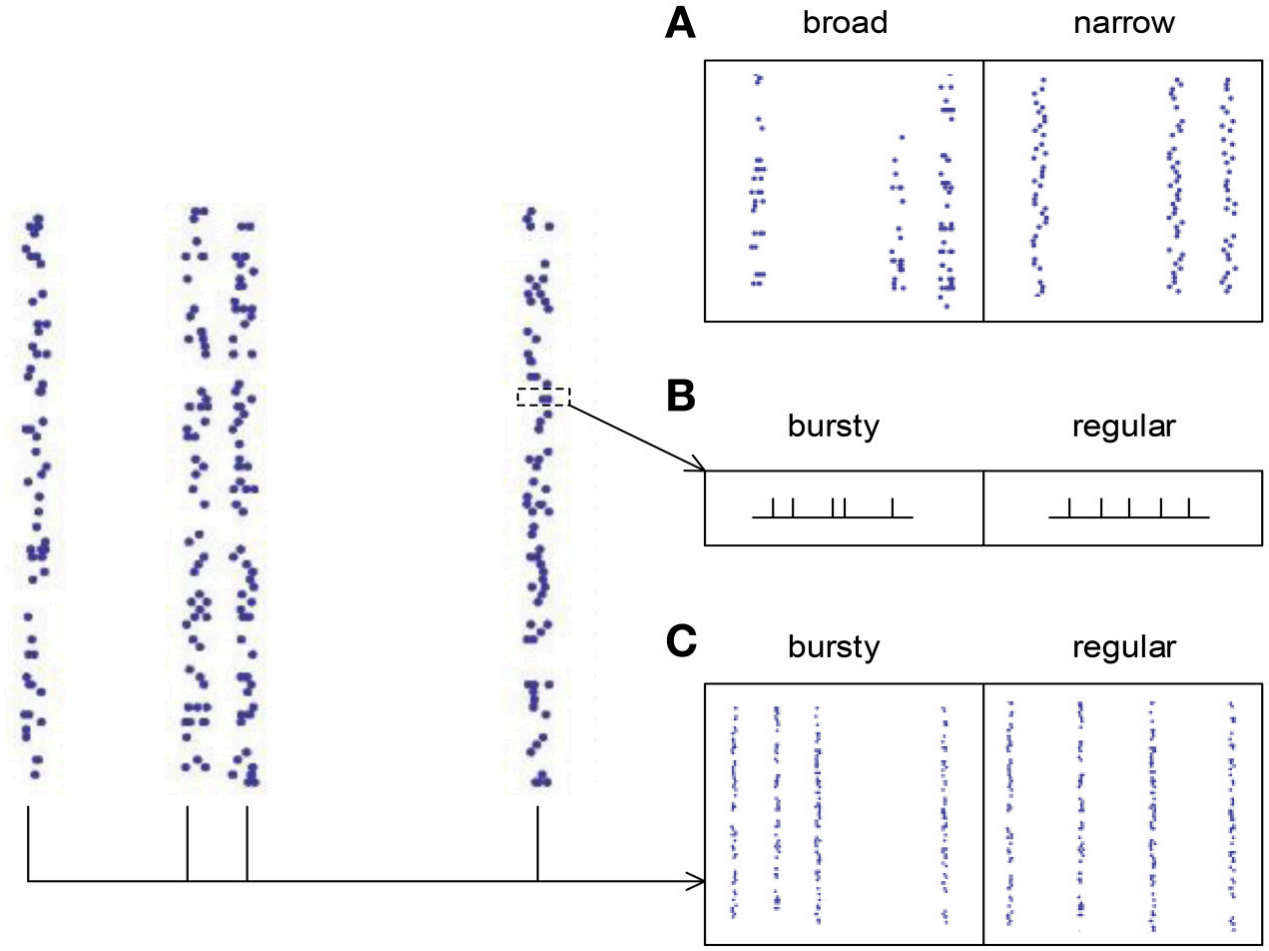
**The three types of auto-correlation structure we consider under synchronous firing**. **(A)** The broadness of the distribution of the spike numbers a neuron fires in different synchronous events (AT_SpikeNum_). Note that in the left panel, a neuron fires quite different number of spikes during different synchronous events; while in the right panel, the spike numbers of a neuron during different synchronous events are almost the same. **(B)** The burstiness/regularity of the pieces of spike trains within synchronous events (AT_WithinEvent_). **(C)** The burstiness/regularity of the occurrence of synchronous events (AT_events_).

The spike pattern of a real neuronal population possesses both synchronous firing and auto-correlation structure, so it is desirable to know how these two pattern structures interact to influence DiffV. This is a complicated problem, because auto-correlation structure comes into spike patterns with synchronous firing in at least three ways (Figure 8):

(1) The broadness of the distribution of the spike numbers a neuron fires in different synchronous events (AT_SpikeNum_). If the distribution gets broad, a neuron may burst several spikes in some synchronous events, while keep silent in some others.
(2) The burstiness/regularity of the pieces of spike trains within synchronous events (AT_WithinEvent_).
(3) The burstiness/regularity of the occurrence of synchronous events (AT_events_).

There may be other auto-correlation structures under synchronous firing. For example, in biological systems, the amplitudes of synchronous events may exhibit strong variability (Gireesh and Plenz, 2008; Petermann et al., 2009), which further increases the complexity of the problem. However, we argue that this varying-amplitude situation may be included into the constant-amplitude scenario using AT_events_, after noting that strong synchronous events can be regarded as the burstiness of many small synchronous events. For simplicity, we will not consider this varying-amplitude situation in our following discussions.

We have already discussed the effect of AT_SpikeNum_ in the previous subsection, and we will perform detailed discussions on how AT_WithinEvent_ and AT_events_ influence DiffV through *d*, *c*_*I*_ and *c*_*II*_ in Supplementary Material Section S4. Here we summarize our main results:

(1) The broadness of the distribution of the spike numbers a neuron fires in different synchronous events increases DiffV through increasing *d* and *c*_*I*_. When τ_*cross*_ ≤ τ_*delay*_, it does not significantly change *c*_*II*_; when τ_*cross*_ > τ_*delay*_, it decreases *c*_*II*_, which negatively contributes to the increase of DiffV (see the previous subsection).
(2) The burstiness of the piece of spike train within a synchronous event significantly increases DiffV when τ_*cross*_ > τ_*delay*_. It does this through increasing *d* and *c*_*II*_, it does not significantly influence *c*_*I*_ (Supplementary Material Section S4.1, Supplementary Figure 3).
(3) The burstiness of the occurrence of synchronous events increases DiffV through increasing *d* and *c*_*I*_. It decreases *c*_*II*_, which negatively contributes to the increase of DiffV (Supplementary Material Section S4.2, Supplementary Figure 7).

In Supplementary Material Section S4 and Supplementary Figures 3–10, we explain the underlying mechanisms of these influences using both numeric and analytic approaches. We find that broader distribution of spike number per neuron per synchronous event, burstier spike trains within synchronous events, and burstier occurrence of synchronous events tend to increase DiffV. These results can be concluded into a rule of thumb: the burstiness of spike trains tends to increase DiffV, while the regularity tends to decrease DiffV.

From Section 3.2, we know that when the spike trains are stationary processes, DiffV increases with the decrease of *CV* if *CV* ≪ 1 (Figure 4A), because of transient cross-correlation. However, here we find that after synchronous firing is added into the spike pattern, DiffV usually monotonically decreases with the decrease of *CV*. To understand this, note that in our model, we suppose that the pieces of spike trains that belong to different synchronous events are independent of each other. In this case, transient cross-correlation may be fragile under synchronous firing, because the synaptic changes caused by two central spikes *t*_*m*_ and *t*_*n*_ (i.e., $\underset{j}{\sum }\Delta w_{a}\left(t_{m},t_{j}\right)$ and $\underset{j}{\sum }\Delta w_{a}\left(t_{n},t_{j}\right)$) are hard to be correlated if *t*_*m*_ and *t*_*n*_ belong to different synchronous events, even if *m* and *n* are nearby by index.

### 3.5. The influence of heterogeneity of rates onto DriftV

Under heterogeneity of rates or heterogeneity of cross-correlations, different synapses may drift with different velocities, thereby inducing DriftV. Heterogeneity of rates and heterogeneity of cross-correlations may also influence the diffusion of synapses. However, as DriftV ∝ *t*^2^ and DiffV ∝ *t*, DriftV will dominate in a long run. Therefore, we will focus on their influence onto DriftV in the main text, their influence on DiffV will be briefly discussed in the Supplementary Materials Section S6.

Suppose that the activities of the central and non-central neurons are time-modulated simultaneously, so that the firing rate of the central neuron *r*_0_(*t*) = 〈*r*_0_(*t*)〉*x*(*t*) while the firing rate of the *a*th non-central neuron *r*_*a*_(*t*) = 〈*r*_*a*_(*t*)〉*x*(*t*), with 〈·〉 representing time average, and *x*(*t*) representing a function with 〈*x*(*t*)〉 = 1. Then, the change of the *a*th synapse per unit time is

(33) $$\begin{array}{l} v_{a}=\frac{{\text{dE}}_{T}(\Delta w_{a})}{\text{d}t}=\int_{0}^{\infty }\text{d}\tau (H(\tau )r_{0}(t)r_{a}(t-\tau_{delay}-\tau )\\ \;+H(-\tau )r_{0}(t-\tau )r_{a}(t-\tau_{delay}))\\ \;=\langle r_{0}(t)\rangle \langle r_{a}(t)\rangle \int_{-\infty }^{\infty }\text{d}\tau H(\tau )C(-\tau_{delay}-\tau ) \end{array}$$

with *H*(τ) being the STDP time window and *C*(−τ_*delay*_ − τ) = 〈*x*(*t*)*x*(*t* − τ_*delay*_ − τ)〉 being the auto-correlation of *x*(*t*). If we average the equation above over *a*, we will have

(34) $$\begin{array}{l} E_{a}\left(v_{a}\right)=\langle r_{0}\left(t\right)\rangle {\text{E}}_{a}\left(\langle r_{a}\left(t\right)\rangle \right)\cdot \int_{-\infty }^{\infty }\text{d}\tau H\left(\tau \right)C\left(-\tau_{delay}-\tau \right) \end{array}$$

The two equations above togther give

(35) $$\begin{array}{l} v_{a}={\text{E}}_{a}\left(v_{a}\right)\cdot \frac{\langle r_{a}\left(t\right)\rangle }{{\text{E}}_{a}\left(\langle r_{a}\left(t\right)\rangle \right)} \end{array}$$

Therefore,

(36) $$\begin{array}{l} {\text{Var}}_{a}\left(v_{a}\right)=\frac{{\left[{\text{E}}_{a}\left(v_{a}\right)\right]}^{2}}{{\left[{\text{E}}_{a}\left(\langle r_{a}\left(t\right)\rangle \right)\right]}^{2}}\cdot {\text{Var}}_{a}\left(\langle r_{a}\left(t\right)\rangle \right) \end{array}$$

**Figure 9:**
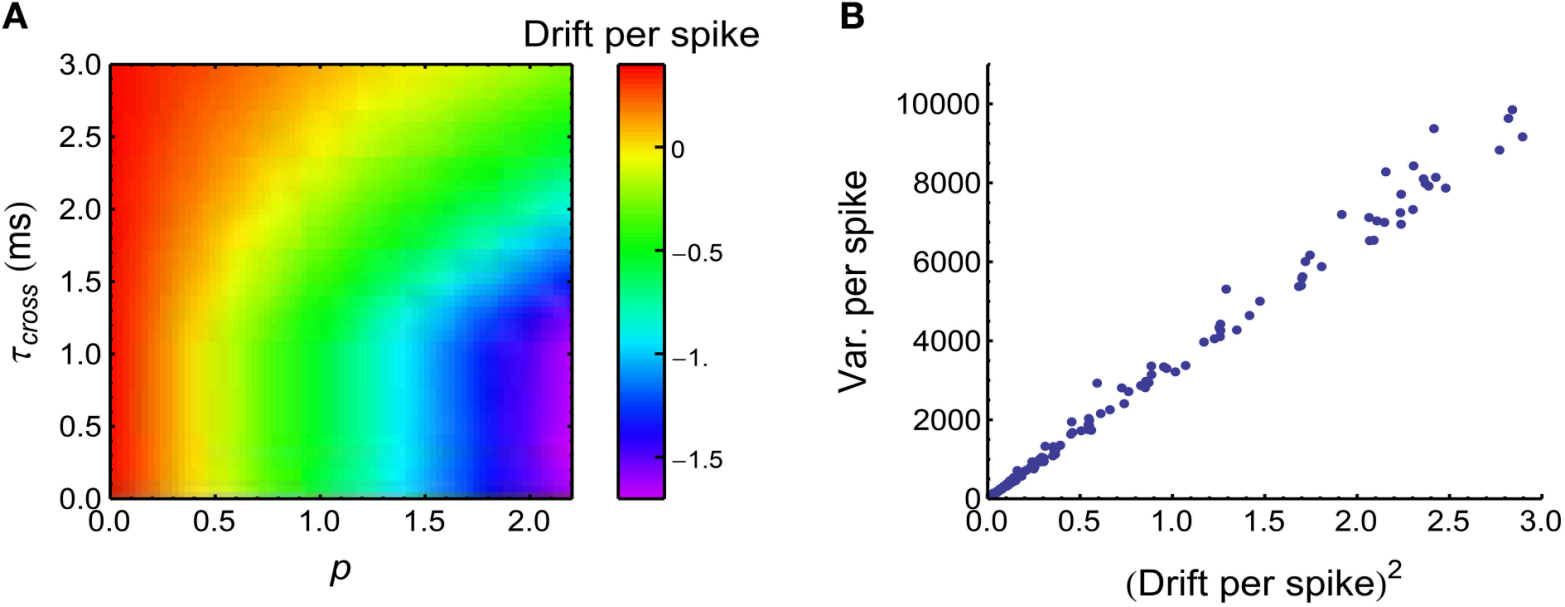
**Synchronous firing influences P-D imbalance, and heterogeneity of rates makes use of P-D imbalance to change DriftV**. **(A)** Drift per spike (${\text{E}}_{a,T}\left(\Delta w_{a}\right)∕{\bar{N}}_{0}$, with ${\bar{N}}_{0}$ being the trial-averaged spike number of the central neuron) as a function of *p* and τ_*cross*_, when the spike trains have synchronous firing and the firing rates of neurons are long-tailed distributed (Model Long Tail in Section 2; *s* = 1). Note that synchronous firing strengthens depression in this model. **(B)** ${\text{Var}}_{a}\left(\Delta w_{a}\right)\propto {\left[{\text{E}}_{a}\left(\Delta w_{a}\right)\right]}^{2}$, which is consistent with Equation 36. Different dots represent different (τ_*cross*_, *p*) pairs that are uniformly distributed in the range of **(A)**. In **(A,B)**, *A*_*p*_ = 2, *A*_*d*_ = 1. The size of the converging motif, and the simulation time and trials are the same as in Figure 4.

This equation suggests that the heterogeneity of rates 〈*r*_*a*_(*t*)〉 can induce DriftV; but if the population rate E_*a*_(〈*r*_*a*_(*t*)〉) is kept constant, DriftV also depends on |E_*a*_(*v*_*a*_)|, which quantifies the imbalance of the strengths of the potentiation and depression process (*P-D imbalance*). If E_*a*_(*v*_*a*_) = 0, which means that STDP cannot change the mean synaptic strength (*P-D balance*), then DriftV = 0; if |E_*a*_(*v*_*a*_)| is large, which means that P-D imbalance is strong, then DriftV is also large. From Equation 34, we know that if the firing rates 〈*r*_0_(*t*)〉 and E_*a*_(〈*r*_*a*_(*t*)〉) are kept constant, then P-D imbalance is controlled by the time-modulated pattern *x*(*t*), which will be changed if synchronous firing is added into the spike pattern. Intuitively, during a synchronous event, the axonal delay in the converging motif tends to make the central neuron receive spikes from the non-central neurons *after* its own spikes, which depresses the synapses under STDP (Figure 9A, a similar phenomenon has been observed in Lubenov and Siapas, 2008). Under heterogeneity of rates, this makes DriftV become accordingly changed (Figure 9B). In summary, heterogeneity of rates makes use of P-D imbalance to induce DriftV; and synchronous firing can influence P-D imbalance, thereby influencing DriftV under heterogeneity of rates.

### 3.6. The interaction of heterogeneity of rates, synchronous firing and auto-correlation structure

From the arguments above, we know that if spike trains are stationary processes (with *x*(*t*) = 1 for any *t*), then auto-correlation structure cannot influences DriftV under heterogeneity of rates (because auto-correlation structure cannot change *x*(*t*)). Its influences onto DiffV in this case are discussed in Supplementary Materials Section S3. Interesting things happen when synchronous firing also comes into the picture. As we mentioned in Section 3.4, under synchronous firing, auto-correlation structure may come into the spike pattern in three ways (Figure 8):

(1) The broadness of the distribution of spike number per neuron per synchronous event (AT_SpikeNum_).
(2) The burstiness/regularity of the pieces of spike trains within synchronous events (AT_WithinEvent_).
(3) The burstiness/regularity of the occurrence of synchronous events (AT_events_).

**Figure 10:**
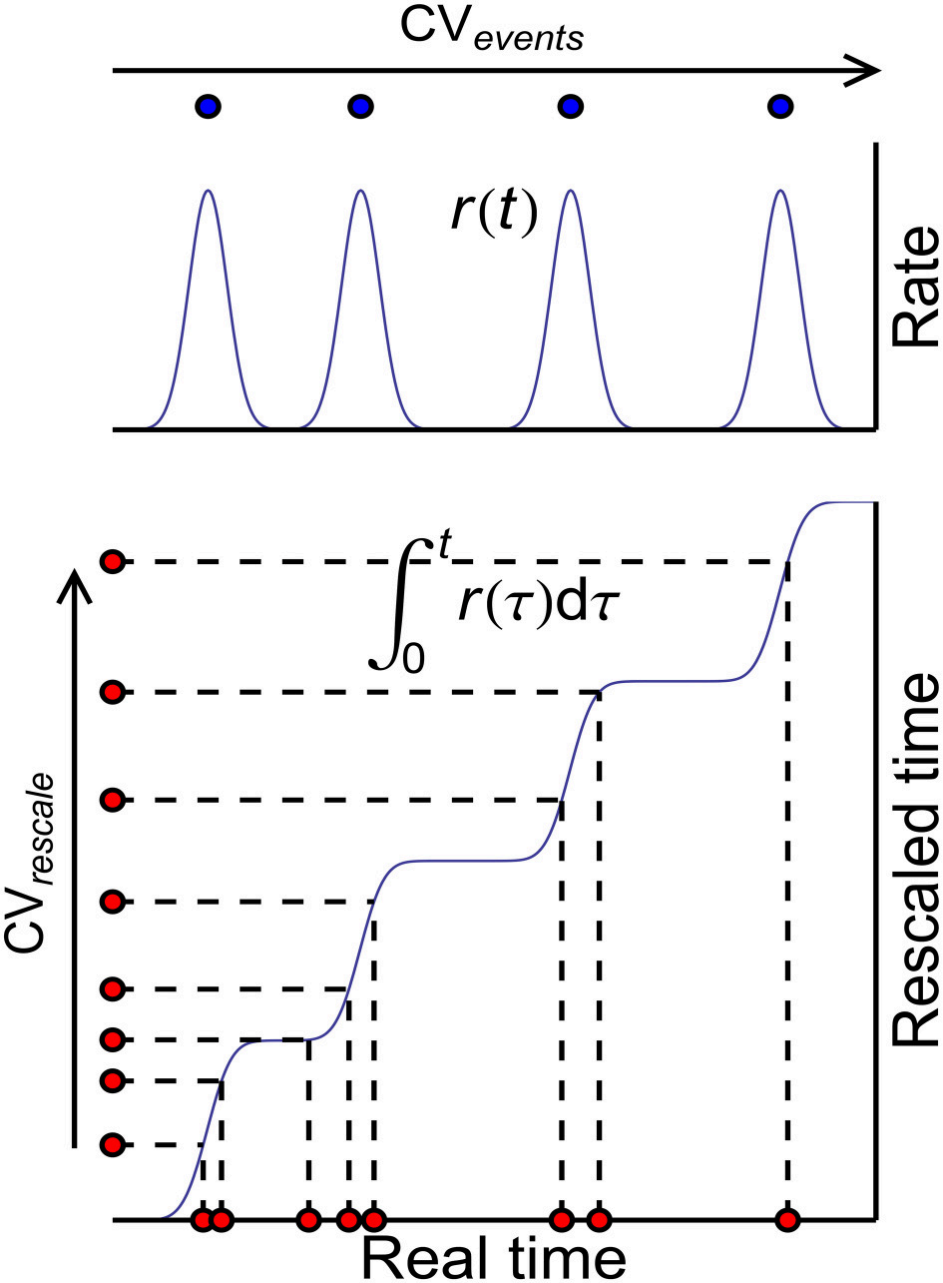
**The scheme to classify auto-correlation structure under synchronous firing**. Blue curves represent the population firing rate (upper) and the accumulative function of the population firing rate (lower) in the real time. Blue dots (upper) represent the times when the synchronous events occur, whose auto-correlation structure is quantified by their *CV* value *CV*_*events*_. Red dots (lower) represent the spikes in the real time and their correspondences in the rescaled time, whose auto-correlation structure is quantified by their *CV* value *CV*_*rescale*_.

These three auto-correlation structures can be classified into two kinds: AT_SpikeNum_ and AT_WithinEvent_ do not change *x*(*t*), so that they do not change P-D imbalance, nor DriftV under heterogeneity of rates; AT_events_, however, changes *x*(*t*), thereby changing P-D imbalance, and DriftV under heterogeneity of rates. An idea to separate these two kinds of auto-correlation structure is to define rescaled time using the accumulative function of population firing rate (Pillow, 2009)

(37) $$\begin{array}{l} \Lambda \left(t\right)=\int_{0}^{t}r\left(s\right)ds, \end{array}$$

which stretches the inter-spike intervals in proportion to the firing rate (Figure 10). Auto-correlation structure then comes into the picture in two ways: the auto-correlation structure of the spikes in the rescaled time, which can be quantified by the *CV* value *CV*_*rescale*_, and the auto-correlation structure of the occurrence of synchronous events, which can be quantified by *CV*_*events*_. Apparently, *CV*_*rescale*_ has no influence on *x*(*t*), thereby nor DriftV under heterogeneity of rates; but *CV*_*events*_ influences *x*(*t*), thereby DriftV under heterogeneity of rates. Combining the discussion on the influence of temporal structure onto DiffV in Section 3.4, we come to the conclusion that under synchronous firing and heterogeneity of rates,

(1) The temporal factors that increases either *CV*_*rescale*_ or *CV*_*events*_ increase DiffV.
(2) The temporal factors that influences *CV*_*rescale*_ do not change P-D imbalance, thereby nor DriftV under heterogeneity of rates.
(3) The temporal factors that influences *CV*_*events*_ change P-D imbalance, thereby DriftV under heterogeneity of rates.

Indeed, we find that both AT_SpikeNum_ and AT_WithinEvent_ increase *CV*_*rescale*_ and don't influence P-D imbalance (Supplementary Figures 11A,B); while AT_events_ increases *CV*_*events*_ and may change P-D imbalance especially when *CV*_*events*_ > 1 (Supplementary Figures 11C,D). The influence of *CV*_*events*_ onto P-D imbalance seems complicated, and depends on the values of τ_*delay*_, *A*_*p*_ and *A*_*d*_ (see Supplementary Figure 12, the physical mechanisms will be explained in Supplementary Material Section S5.2):

(1) Suppose during a synchronous event S_0_, the central neuron fires at time *t*_0_. Because of the axonal delay τ_*delay*_, there is usually a time interval between *t*_0_ and when the spikes from non-central neurons arrive at the axonal terminal, and the typical length of this interval is τ_*delay*_. If synchronous events are *not* close to each other, so that no non-central spikes from synchronous events *other than* S_0_ arrive at the central neuron during this interval (i.e., different synchronous events do not *overlap* with each other), then E_*a, T*_(Δ*w*_*a*_) will increases (or decreases) with *CV*_*events*_ if *A*_*p*_exp(τ_*delay*_∕τ_*STDP*_) > *A*_*d*_exp(−τ_*delay*_∕τ_*STDP*_) (or *A*_*p*_exp(τ_*delay*_∕τ_*STDP*_) < *A*_*d*_exp(−τ_*delay*_∕τ_*STDP*_)). In this paper, typically τ_*delay*_ ≪ τ_*STDP*_, so these conditions become *A*_*p*_ > *A*_*d*_ or *A*_*p*_<*A*_*d*_.
(2) If synchronous events are allowed to overlap with each other, then *CV*_*events*_ will increase the chance of this overlapping when it is large (typically when *CV*_*events*_ > 1). In this case, E_*a, T*_(Δ*w*_*a*_) will decrease (or increase) with *CV*_*events*_ if τ_*delay*_ > 0 (or τ_*delay*_ < 0). See the meaning of τ_*delay*_ < 0 in Figure 2B.

**Figure 11:**
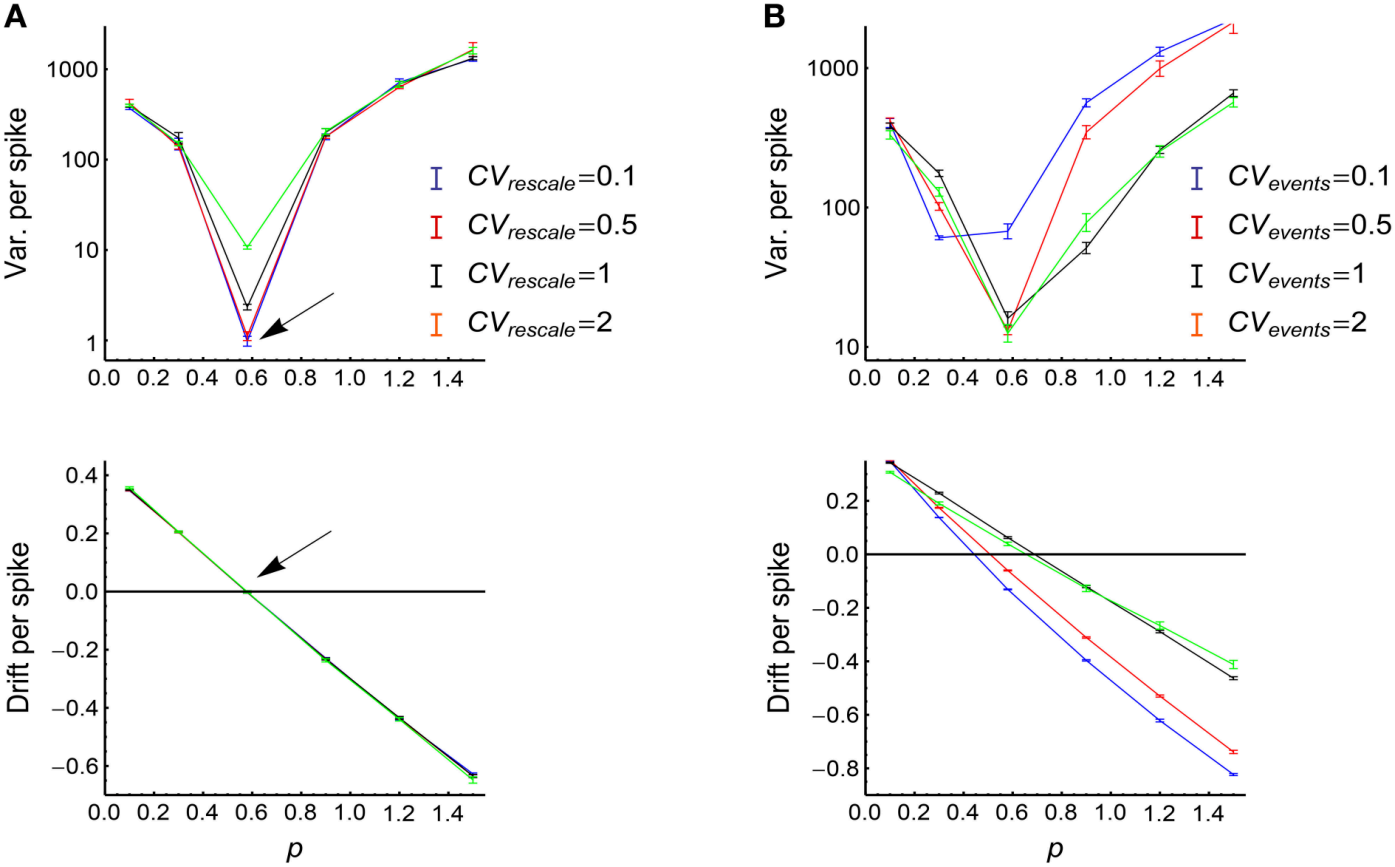
**The influence of auto-correlation structure onto the efficacy variability under synchronous firing and heterogeneity of rates**. **(A)** Upper: Variance per spike as a function of *p* at different *CV*_*rescale*_, keeping *CV*_*events*_ = 0.71 and *s* = 1 (see Model Sync-Auto-LongTail in Section 2). Lower: the corresponding mean efficacy changes per spike, which represent P-D imbalance. The arrows indicate the *p* value at which the mean efficacy change is almost zero (indicated by the horizontal black line), so DriftV ≈ 0 at this point due to P-D balance. Error bars represent s.e.m. in normal scale and relative errors corresponding to s.e.m. in log scale. **(B)** The same as **(A)** except that different lines represent different *CV*_*events*_ values, keeping *CV*_*rescale*_ = 0.71. In **(A,B)**, τ_*cross*_ = 2ms, *A*_*p*_ = 2, *A*_*d*_ = 1, τ_*delay*_ = 1 ms. The size of the converging motif, as well as the simulation time and trials are the same as in Figure 4.

To get a better understanding on how heterogeneity of rates, synchronous firing and auto-correlation structure interact with each other, we used Model Sync-Auto-LongTail (see Section 2) to generate spikes. In this model, for simplicity, the auto-correlation structure in the rescaled time and the auto-correlation structure of the occurrence of the synchronous events are respectively controlled by a single parameter (i.e., *CV*_*rescale*_ and *CV*_*events*_). We generated spike patterns using this model, and investigated how the efficacy variability changes with model parameters (Figure 11). The results validate our arguments above:

(1) *CV*_*rescale*_ hardly influences P-D imbalance (Figure 11A, lower panel).
(2) When the strength of firing events is adjusted so that the potentiation and depression almost balance each other (so that DriftV ≈ 0), the efficacy variability significantly increases with *CV*_*rescale*_ (Figure 11A, upper panel). However, when the potentiation and depression are imbalanced, the efficacy variability becomes hardly influenced by *CV*_*rescale*_ (Figure 11A, upper panel). This is because that *CV*_*rescale*_ can only increase DiffV, and can hardly influence DriftV. As DiffV ∝ *t* while DriftV ∝ *t*^2^, the contribution of *CV*_*rescale*_ to efficacy variability after a long run is only obvious when DriftV ≈ 0, which is realized at P-D balance.
(3) The influence of *CV*_*events*_ onto efficacy variability is non-monotonically complicated (Figure 11B, upper panel). The reason is that *CV*_*events*_ not only influences DiffV, but also influences DriftV in a complicated way (Figure 11B, lower panel), and DriftV is also influenced by *p*. Therefore, potentiation and depression are balanced at different *p* values for different *CV*_*events*_ values.

### 3.7. The influence of heterogeneity of cross-correlations onto DriftV

Heterogeneity of cross-correlations mainly influences efficacy variability in DriftV manner. Suppose the firing rate of the central neuron is *r*_0_(*t*) = *r*_0_·*x*_0_(*t*) and the firing rate of the *a*th non-central neuron is *r*_*a*_(*t*) = *r*_*a*_·*x*_*a*_(*t*), with *r*_0_ and *r*_*a*_ being their time-averaged firing rates and 〈*x*_0_(*t*)〉 = 〈*x*_*a*_(*t*)〉 = 1 (here 〈·〉 represent time average), then similarly as Equation 33, we have

$$v_{a}=r_{0}r_{a}\int_{-\infty }^{\infty }\text{d}\tau H\left(\tau \right)C_{a}\left(-\tau_{delay}-\tau \right),$$

with *C*_*a*_(τ) = 〈*x*_0_(*t*)*x*_*a*_(*t* + τ)〉 being the *unit cross-correlation* between the central neuron and the *a*th non-central neuron. Therefore,

(38) $$\begin{array}{l} {\text{Var}}_{a}\left(v_{a}\right)=r_{0}^{2}{\text{Var}}_{a}\left[r_{a}\int_{-\infty }^{\infty }\text{d}\tau H\left(\tau \right)C_{a}\left(-\tau_{delay}-\tau \right)\right], \end{array}$$

so the heterogeneity of *C*_*a*_(τ) influences DriftV together with the heterogeneity of *r*_*a*_.

For simplicity, we did not investigate how the heterogeneity of *C*_*a*_(τ) interact with other pattern structures. Under STDP, the main effect of the heterogeneity of *C*_*a*_(τ) is to influence DriftV with its interaction with the heterogeneity of firing rates through Equation 38, which will not change even when the other pattern statistics are considered. The influence of the heterogeneity of *C*_*a*_(τ) onto DriftV can be understood by comparing Equation 38 with

$$r_{0}^{2}{\text{E}}_{a}{\left[\int_{-\infty }^{\infty }\text{d}\tau H\left(\tau \right)C_{a}\left(-\tau_{delay}-\tau \right)\right]}^{2}{\text{Var}}_{a}\left(r_{a}^{2}\right)=\frac{{\text{E}}_{a}{\left(v_{a}\right)}^{2}}{{\text{E}}_{a}{\left(r_{a}\right)}^{2}}{\text{Var}}_{a}\left(r_{a}\right),$$

which quantifies the DriftV caused by heterogeneity of rates under P-D imbalance.

### 3.8. The case when τ_*delay*_ < 0

In the discussions above, we generally suppose that τ_*delay*_ ≥ 0, which means that the axonal delay for pre-synaptic spikes is no smaller than the dendritic delay for post-synaptic back-propagated spikes. Here we discuss the case when τ_*delay*_ < 0, i.e., the dendritic delay is larger than the axonal delay.

To understand the relationship between the case τ_*delay*_ > 0 and the case τ_*delay*_ < 0, let us first consider a synapse from neuron *a* to neuron *b* in the following two cases. Case I: suppose neuron *a* (neuron *b*) fires a spike at time *t*_*a*_ (*t*_*b*_), and *A*_*p*_ = *A*, *A*_*d*_ = *B* in the STDP time window Equation 6. In this case, whether the synapse is potentiated or depressed depends on the time difference between *t*_*b*_ and *t*_*a*_ + τ_*delay*_ (i.e., the sign of Δ*t*_1_ ≡ *t*_*b*_ − (*t*_*a*_ + τ_*delay*_)). Case II: now we let the spike delay become −τ_*delay*_, and *A*_*p*_ = *B*, *A*_*d*_ = *A* in Equation 6, and we also reverse the spike trains (just like showing the two neurons a backward movie), then neuron *a* will fire at time *T*−*t*_*a*_ (with *T* being the total duration of the spike pattern), and neuron *b* will fire at time *T*−*t*_*b*_. In this case, whether the synapse is potentiated or depressed depends on the sign of Δ*t*_2_ ≡ (*T* − *t*_*b*_) − ((*T* − *t*_*a*_) − τ_*delay*_) = −Δ*t*_1_. If Δ*t*_1_ > 0, then the synapse in Case I will be potentiated by *A*exp(−Δ*t*_1_∕τ_*STDP*_), and the synapse in Case II will be depressed by −*A*exp(−Δ*t*_1_∕τ_*STDP*_); if Δ*t*_1_ < 0, then the synapse in Case I will be depressed by −*B*exp(Δ*t*_1_∕τ_*STDP*_), and the synapse in Case II will be potentiated by *B*exp(Δ*t*_1_∕τ_*STDP*_). After applying this argument to every pair of spikes in the spike trains of the central neuron and the *a*th non-central neuron in a converging motif, we have that


(39) $$\begin{array}{l} \Delta w_{a,p}\left(\text{Case I}\right)=-\Delta w_{a,d}\left(\text{Case II}\right), \end{array}$$

(40) $$\begin{array}{l} \Delta w_{a,d}\left(\text{Case I}\right)=-\Delta w_{a,p}\left(\text{Case II}\right). \end{array}$$

Therefore, the total synaptic change Δ*w*_*a*_ = Δ*w*_*a, p*_ + Δ*w*_*a, d*_ satisfies

(41) $$\begin{array}{l} \Delta w_{a}\left(\text{Case I}\right)=-\Delta w_{a}\left(\text{Case II}\right), \end{array}$$

which means that

(42) $$\begin{array}{l} E_{a}\left[\Delta w_{a}\left(\text{Case I}\right)\right]=-{\text{E}}_{a}\left[\Delta w_{a}\left(\text{Case II}\right)\right], \end{array}$$

but

(43) $$\begin{array}{l} {\text{Var}}_{a}\left[\Delta w_{a}\left(\text{Case I}\right)\right]={\text{Var}}_{a}\left[\Delta w_{a}\left(\text{Case II}\right)\right]. \end{array}$$

The spike trains generated by our statistical models (Section 2.3) are all statistically time-reversal invariant (i.e., people cannot tell whether a spike pattern is forward or backward by analyzing its statistics). Therefore, from Equation 43, our results on how auto-correlation structure, synchronous firing and heterogeneity of rates influence the efficacy variability are all valid for τ_*delay*_ < 0, after replacing τ_*delay*_ by |τ_*delay*_|. The influence of heterogeneity of cross-correlations can be obtained by directly substituting τ_*delay*_ < 0 into Equation 38.

### 3.9. A summary on the efficacy variability in converging motifs

Here we summarize our results in the previous subsections on the efficacy variability in converging motifs.

#### 3.9.1. DiffV

(1) In stationary spike trains (i.e., spike trains with constant trial-averaged firing rate over time) with *homogeneity* of rates, DiffV gets large either when the spike trains get bursty or strongly regular, and DiffV is smallest when *CV* is within the range 0.3~0.7 (Section 3.2). The only reason why DiffV is large under strong regularity and homogeneity of rates is transient cross-correlation. In stationary spike trains with *heterogeneity* of rates, DiffV tends to monotonically increase with *CV* (Supplementary Material Section S2), because the transient cross-correlation under strong regularity is destroyed by heterogeneity of rates.
(2) In stationary spike trains, heterogeneity of rates does not strongly influence DiffV when the spike trains are bursty, but discounts the increase of DiffV with the decrease of *CV* under strong regularity (Supplementary Material Section S3).
(3) Synchronous firing usually increases DiffV, except when τ_*cross*_ ≤ |τ_*delay*_| and each non-central neuron fires almost the same number of spikes during a synchronous event (Section 3.3).
(4) Under synchronous firing, auto-correlation structure may have a number of types, such as the broadness of the distribution of the spike numbers a neuron fires in different synchronous events (AT_SpikeNum_), the burstiness/regularity of the pieces of spike trains within synchronous events (AT_WithinEvent_), and the burstiness/regularity of the occurrence of synchronous events (AT_events_). We find that broader distribution of spike number per neuron per synchronous event, burstier spike trains within synchronous events, and burstier occurrence of synchronous events tend to increase DiffV (Section 3.4). These findings can be concluded into a rule of thumb: the burstiness of spike trains tends to increase DiffV, while the regularity tends to decrease DiffV.

#### 3.9.2. DriftV

(1) Auto-correlation structure in stationary spike trains does not influence DriftV.
(2) Heterogeneity of rates makes use of P-D imbalance to induce DriftV; and synchronous firing can change P-D imbalance, thereby changing DriftV under heterogeneity of rates (Section 3.5).
(3) Under synchronous firing, auto-correlation structure can be classified into two classes using rescaled-time transform (Figure 10): the factors that influence *CV*_*rescale*_ (here, AT_SpikeNum_ and AT_WithinEvent_) and the factors that influence *CV*_*events*_ (here, AT_events_). The factors that influence *CV*_*rescale*_ do not change P-D imbalance, thereby nor DriftV under heterogeneity of rates; while the factors that influence *CV*_*events*_ change P-D imbalance, thereby DriftV under heterogeneity of rates (Section 3.6). The influence of *CV*_*events*_ onto P-D imbalance is complicated (see Supplementary Materials Section S6.2 for detailed discussions); but if synchronous events are not too close to each other, the synapses will get stronger (or weaker) with the increase of *CV*_*events*_ if *A*_*p*_exp(τ_*delay*_∕τ_*STDP*_) > *A*_*d*_exp(−τ_*delay*_∕τ_*STDP*_) (or *A*_*p*_exp(τ_*delay*_∕τ_*STDP*_) < *A*_*d*_exp(−τ_*delay*_∕τ_*STDP*_)).
(4) Heterogeneity of cross-correlations can induce DriftV together with heterogeneity of rates (Section 3.7).

## 4. Discussion

In this paper, we systematically studied the influences of four aspects of pattern structures (i.e., synchronous firing, auto-correlation structure, heterogeneity of rates and heterogeneity of cross-correlations, as well as their interactions) onto efficacy variability under STDP, using spike generating models on converging motifs. We separated efficacy variability into two parts: the variability induced by the heterogeneity of change rates of different synapses (DriftV), and the variability induced by weight diffusion caused by stochasticity of spike trains during learning (DiffV). Our main findings are that both synchronous firing and burstiness in auto-correlation structure tend to increase DiffV, heterogeneity of rates induces DriftV when potentiation and depression in STDP are not balanced (P-D imbalance), and heterogeneity of cross-correlations induces DriftV together with heterogeneity of rates.

The reason why we focused on these four aspects of pattern structures is that under STDP they are the only four that mainly influence the lowest order of DiffV and DriftV: for DiffV we only investigated the mean diffusion strength over synapses (i.e., E_*a*_(Var_*T*_(Δ*w*_*a*_)), with *a* being the index of non-central neurons, and *T* representing integration over trials), neglecting the possible heterogeneity of diffusion strengths for different synapses; and for DriftV we did not consider correlations among drift velocities of synapses. Strictly speaking, heterogeneity of rates and heterogeneity of cross-correlations may not only make different synapses drift at different velocities, but also make them have different diffusion strengths. However, as DriftV ∝ *t*^2^ while DiffV ∝ *t*, the heterogeneity of diffusion strengths caused by them is only important when DriftV≈0. For simplicity, we did not seriously consider their influences onto the heterogeneity of diffusion strengths in this study, but only briefly discussed it in Supplementary Materials Section S6.

In this paper, the spike trains of the central neurons in converging motifs are generated according to purely statistical models, instead of integrating their inputs. By doing this, we are able to focus to investigate the influence of spike pattern statistics to the efficacy variability, without worrying about the feedback from synaptic changes to the spike trains of the central neurons. As we mentioned in the introduction, our spike trains are used to mimick the spike patterns of recurrent networks in which the dynamics of the neurons are almost the same. Although biological neurons *do* fire spikes by integrating their inputs, we presume that if we replace the central neurons in converging motifs with a biologically more plausible model (such as integrate-and-fire neurons), the difference between the obtained results from the results in this paper will be limited. For example, if the spike patterns of the pre-synaptic neurons have synchronous firing, then the post-synaptic neuron will also be likely to fire immediately after the synchronous events of the pre-synaptic neurons. The time delay between the firing of the pre- and post-synaptic neurons can be absorbed into the STDP parameter τ_*delay*_ in our model. If the pre-synaptic neurons fire burstily (regularly), the post-synaptic neurons may also tend to fire burstily (regularly), because of its stronger (regular) input fluctuation. Worsely, if we replace the central neurons with integrate-and-fire neurons, the post-synaptic spike trains will depend on neuronal model parameters, and their statistics will also continuously change during the plasticity of the synapses. These will make the results less convincing.

In our STDP model, synaptic updatings do not depend on the current weights, and the contributions of all spike pairs are added together. We chose this STDP model largely because of its simplicity, especially for analytic treatment. This form of STDP has been used in the literature to study, for example, the generation of functional maps (Song and Abbott, 2001; Widloski and Fiete, 2014) and synfire chains (Jun and Jin, 2007; Fiete et al., 2010) during development, the organization of feedforward or recurrent connections to enhance the processing of correlation or temporal information (Kistler and van Hemmen, 2000; Gilson et al., 2009a,b), the generation of a negative sensory image (Roberts and Bell, 2000), and the control of synchrony (Lubenov and Siapas, 2008) and the evolution of connectivity (Babadi and Abbott, 2013; Ocker et al., 2015) within a recurrent network. Our work provides an angle to review these works, e.g., to understand why these models work in spite of the noises of plasticity, to investigate how the functional performances of STDP may be influenced by the second-order statistics of spike patterns.

There has been literature about the influence of the second-order spike pattern statistics onto the synaptic evolution under STDP. The theoretical works using phenomenological STDP models like ours usually focused on the generation of input selectivity when a post-synaptic neuron or a recurrent network is driven by two pools of inputs with different cross-correlations (Gütig et al., 2003; Gilson et al., 2009a,b,c, 2010). There have been also analytical discussions about the increase of the efficacy variability under Poisson spike trains (Kempter et al., 1999; Gilson et al., 2009a). Comparing to these works, our work has two aspects of contributions. Firstly, we provide an angle to investigate the second-order statistics of spike patterns in a physically intuitive way. To do this, we separated the second-order statistics into four parts (i.e., synchronous firing, auto-correlation structure, heterogeneity of rates and heterogeneity of cross-correlations, see Figure 2A), and considered three types of auto-correlation structure (see Figure 8) under spike patterns with synchronous events. All these statistics have clear physical meanings, which helps to build an qualitative understanding on spike patterns. Secondly, we provide a theoretical framework to understand how each of these second-order statistics influences efficacy variability. DriftV under heterogeneity of rates and heterogeneity of cross-correlations can be calculated using the trial-averaged synaptic change rates (Equations 36, 38), which has been done in a large body of literature (e.g., Kempter et al., 1999; Meffi et al., 2006; Gilson et al., 2009a,b,c). To understand the influence onto DiffV, we investigated the change of three parameters (i.e., *d*, *c*_*I*_ and *c*_*II*_) with pattern structures, each of which has clear physical meaning (see Figure 3). This framework can also be applied to study the influence of spike pattern structures onto efficacy variability under other STDP rules. Therefore, we believe our work will facilitate computational researchers to understand the simulation results of their models, and also provide a new angle for experimentalists to interpret their observations.

The STDP time window may have a variety of forms of complex realization, depending on synaptic types, spike patterns and even locations of synapses on dendrites (Caporale and Dan, 2008), and may also depend on the current synaptic weight (Bi and Poo, 1998). STDP may also have a variety of spike pairing schemes (Burkitt et al., 2004; Morrison et al., 2008). We used a STDP model with all-to-all pairing scheme in this paper; and we presume that our results may be more sensitive to the spike pairing scheme than to the shape of STDP time window. Synchronous firing gathers more pre- and post-synaptic spikes closer, which strengthens the STDP interactions between them, so that the variability of spike trains will be more likely to transform into the variability of synaptic weights. As a result, the increase of DiffV with synchronous firing seems to be universal as long as STDP accomplishes large synaptic changes for spike pairs that are adjacent in time. Stronger burstiness induces stronger variability of spike times and also stronger correlations between synaptic updatings caused by adjacent spikes (note the increased *d* and *c*_*I*_ in Figure 4), which may also increase DiffV under other STDP time windows. However, in nearest neighbor spike pairing schemes (Burkitt et al., 2004), a pre-synaptic (post-synaptic) spike may shield other spikes immediately before (after) it from STDP interactions, thereby reducing efficacy variability if spikes are gathered too close by synchronous firing or bursts.

Although the influence of spike patterns varies across systems, the concept of *efficacy variability* should be of general importance. The stochasticity of synapses and neuronal responses as well as the emergent heterogeneity of rates and cross-correlations in network dynamics together make efficacy variability an unavoidable nature of plasticity. Therefore, it is of great meaning to understand how animals make use of efficacy variability and get around of it in future researches. We believe that the concept of efficacy variability not only provides a new perspective to understand the function of plasticity, but is also a new angle to review our current knowledge on learning.

## Author contributions

ZB and CZ conceived the idea and designed the research. ZB performed the research. ZB and CZ wrote the paper.

### Conflict of interest statement

The authors declare that the research was conducted in the absence of any commercial or financial relationships that could be construed as a potential conflict of interest.

## References

[B1] Allen C. Stevens C. F. (1994). An evaluation of causes for unreliability of synaptic transmission. Proc. Natl. Acad. Sci. U.S.A. 91, 10380–10383. 10.1073/pnas.91.22.10380 7937958 PMC45023

[B2] Babadi B. Abbott L. F. (2013). Pairwise analysis can account for network structures arising from spike-timing dependent plasticity. PLoS Comput. Biol. 9:e1002906. 10.1371/journal.pcbi.1002906 23436986 PMC3578766

[B3] Bartos M. Vida I. Jonas P. (2007). Synaptic mechanisms of synchronized gamma oscillations in inhibitory interneuron networks. Nat. Rev. Neurosci. 8, 45–56. 10.1038/nrn2044 17180162

[B4] Bi G. Q. Poo M. M. (1998). Synaptic modifications in cultured hippocampal neurons: dependence on spike timing, synaptic strength, and postsynaptic cell type. J. Neurosci. 18, 10464–10472. 9852584 10.1523/JNEUROSCI.18-24-10464.1998 PMC6793365

[B5] Brunel N. (2000). Dynamics of sparsely connected networks of excitatory and inhibitory spiking neurons. J. Comput. Neurosci. 8, 183–208. 10.1023/A:1008925309027 10809012

[B6] Brunel N. Hakim V. (1999). Fast global oscillations in networks of integrate-and-fire neurons with low firing rates. Neural Comput. 11, 1621–1671. 10.1162/089976699300016179 10490941

[B7] Burkitt A. N. Meffin H. Grayden D. B. (2004). Spike-timing-dependent plasticity: the relationship to rate-based learning for models with weight dynamics determined by a stable fixed point. Neural Comput. 16, 885–940. 10.1162/089976604773135041 15070504

[B8] Buzsáki G. Draguhn A. (2004). Neuronal oscillations in cortical networks. Science 304, 1926–1929. 10.1126/science.1099745 15218136

[B9] Buzsáki G. Mizuseki K. (2014). The log-dynamic brain: how skewed distributions affect network operations. Nat. Rev. Neurosci. 15, 264–278. 10.1038/nrn3687 24569488 PMC4051294

[B10] Cancedda L. Poo M. M. (2009). Synapse formation and elimination: competition and the role of activity, in Encyclopedia of Neuroscience, ed Squire L. R. (Oxford: Academic Press), 697–703.

[B11] Caporale N. Dan Y. (2008). Spike timing-dependent plasticity: a Hebbian learning rule. Annu. Rev. Neurosci. 31, 25–46. 10.1146/annurev.neuro.31.060407.125639 18275283

[B12] Clause A. Kim G. Sonntag M. Weisz C. J. Vetter D. E. Rübsamen R.. (2014). The precise temporal pattern of prehearing spontaneous activity is necessary for tonotopic map refinement. Neuron 82, 822–835. 10.1016/j.neuron.2014.04.001 24853941 PMC4052368

[B13] Cox D. R. (1962). Renewal Theory. London: Chapman and Hall.

[B14] Dan Y. Poo M. M. (2006). Spike timing-dependent plasticity: from synapse to perception. Physiol. Rev. 86, 1033–1048. 10.1152/physrev.00030.2005 16816145

[B15] Dragoi G. Buzsáki G. (2006). Temporal encoding of place sequences by hippocampal cell assemblies. Neuron 50, 145–157. 10.1016/j.neuron.2006.02.023 16600862

[B16] Fiete I. R. Senn W. Wang C. Hahnloser R. H. R. (2010). Spike time-dependent plasticity and heterosynaptic competition organize networks to produce long scale-free sequences of neural activity. Neuron 65, 563–576. 10.1016/j.neuron.2010.02.003 20188660

[B17] Funahashi S. Inoue M. (2000). Neuronal interactions related to working memory processes in the primate prefrontal cortex revealed by cross-correlation analysis. Cereb. Cortex 10, 535–551. 10.1093/cercor/10.6.535 10859132

[B18] Ganguli S. Latham P. (2009). Feedforward to the past: the relation between neuronal connectivity, amplification, and short-term memory. Neuron 61, 499–501. 10.1016/j.neuron.2009.02.006 19249270

[B19] Gerstner W. Kempter R. van Hemmen J. L. Wagner H. (1996). A neuronal learning rule for sub-millisecond temporal coding. Nature 383, 76–78. 10.1038/383076a0 8779718

[B20] Gilson M. Burkitt A. N. Grayden D. B. Thomas D. A. van Hemmen J. L. (2009a). Emergence of network structure due to spike-timing-dependent plasticity in recurrent neuronal networks. I. Input selectivity-strengthening correlated input pathways. Biol. Cybern. 101, 81–102. 10.1007/s00422-009-0319-4 19536560

[B21] Gilson M. Burkitt A. N. Grayden D. B. Thomas D. A. van Hemmen J. L. (2009b). Emergence of network structure due to spike-timing-dependent plasticity in recurrent neuronal networks. II. Input selectivity-symmetry breaking. Biol. Cybern. 101, 103–114. 10.1007/s00422-009-0320-y 19536559

[B22] Gilson M. Burkitt A. N. Grayden D. B. Thomas D. A. van Hemmen J. L. (2009c). Emergence of network structure due to spike-timing-dependent plasticity in recurrent neuronal networks IV: structuring synaptic pathways among recurrent connections. Biol. Cybern. 101, 427–444. 10.1007/s00422-009-0346-1 19937070

[B23] Gilson M. Burkitt A. N. Grayden D. B. Thomas D. A. van Hemmen J. L. (2010). Emergence of network structure due to spike-timing-dependent plasticity in recurrent neuronal networks V: self-organization schemes and weight dependence. Biol. Cybern. 103, 365–386. 10.1007/s00422-010-0405-7 20882297

[B24] Gireesh E. D. Plenz D. (2008). Neuronal avalanches organize as nested theta- and beta/gamma oscillations during development of cortical layer 2/3. Proc. Natl. Acad. Sci. U.S.A. 105, 7576–7581. 10.1073/pnas.0800537105 18499802 PMC2396689

[B25] Gütig R. Aharonov R. Rotter S. Sompolinsky H. (2003). Learning input correlations through nonlinear temporally asymmetric Hebbian plasticity. J. Neurosci. 23, 3697–3714. Available online at: http://www.jneurosci.org/content/23/9/3697.full 12736341 10.1523/JNEUROSCI.23-09-03697.2003 PMC6742165

[B26] Gutnisky D. A. Josić K. (2010). Generation of spatiotemporally correlated spike trains and local field potentials using a multivariate autoregressive process. J. Neurophysiol. 103, 2912–2930. 10.1152/jn.00518.2009 20032244

[B27] Jacob V. Petreanu L. Wright N. Svoboda K. Fox K. (2012). Regular spiking and intrinsic bursting pyramidal cells show orthogonal forms of experience-dependent plasticity in layer V of barrel cortex. Neuron 73, 391–404. 10.1016/j.neuron.2011.11.034 22284191 PMC3524456

[B28] Jun J. K. Jin D. (2007). Development of neural circuitry for precise temporal sequences through spontaneous activity, axon remodeling, and synaptic plasticity. PLoS ONE 2:e723. 10.1371/journal.pone.0000723 17684568 PMC1933597

[B29] Kamioka H. Maeda E. Jimbo Y. Robinson H. P. C. Kawana A. (1996). Spontaneous periodic synchronized bursting during formation of mature patterns of connections in cortical cultures. Neurosci. Lett. 206, 109–112. 10.1016/S0304-3940(96)12448-4 8710163

[B30] Kempter R. Gerstner W. van Hemmen J. L. (1999). Hebbian learning and spiking neurons. Phys. Rev. E 59, 4498–4515. 10.1103/PhysRevE.59.4498

[B31] Kistler W. M. van Hemmen J. L. (2000). Modeling synaptic plasticity in conjunction with the timing of preand postsynaptic action potentials. Neural Comput. 12, 385–405. 10.1162/089976600300015844 10636948

[B32] Kohn A. Smith M. A. (2005). Stimulus dependence of neuronal correlation in primary visual cortex of the macaque. J. Neurosci. 25, 3661–3673. 10.1523/JNEUROSCI.5106-04.2005 15814797 PMC6725370

[B33] Krumin M. Shoham S. (2009). Generation of spike trains with controlled auto- and cross-correlation functions. Neural Comput. 21, 1642–1664. 10.1162/neco.2009.08-08-847 19191596

[B34] Long M. A. Jin D. Z. Fee M. S. (2010). Support for a synaptic chain model of neuronal sequence generation. Nature 468, 394–399. 10.1038/nature09514 20972420 PMC2998755

[B35] Lubenov E. V. Siapas A. G. (2008). Decoupling through synchrony in neuronal circuits with propagation delays. Neuron 58, 118–131. 10.1016/j.neuron.2008.01.036 18400168

[B36] Macke J. H. Berens P. Ecker A. S. Tolias A. S. Bethge M. (2009). Generating spike trains with specified correlation coefficients. Neural Comput. 21, 397–423. 10.1162/neco.2008.02-08-713 19196233

[B37] Mainen Z. F. Sejnowski T. J. (1995). Reliability of spike timing in neocortical neurons. Science 268, 1503–1506. 10.1126/science.7770778 7770778

[B38] Markram H. Gerstner W. Sjöström P. J. (2012). Spike-timing-dependent plasticity: a comprehensive overview. Front. Synaptic Neurosci. 4:2. 10.3389/fnsyn.2012.00002 22807913 PMC3395004

[B39] Meffi H. Besson J. Burkitt A. N. Grayden D. B. (2006). Learning the structure of correlated synaptic subgroups using stable and competitive spike-timing-dependent plasticity. Phys. Rev. E 73:041911. 10.1103/PhysRevE.73.041911 16711840

[B40] Mongillo G. Barak O. Tsodyks M. (2008). Synaptic theory of working memory. Science 319, 1543–1546. 10.1126/science.1150769 18339943

[B41] Morrison A. Diesmann M. Gerstner W. (2008). Phenomenological models of synaptic plasticity based on spike timing. Biol. Cybern. 98, 459–478. 10.1007/s00422-008-0233-1 18491160 PMC2799003

[B42] Nawrot M. P. Boucsein C. Molina V. R. Riehle A. Aertsen A. Rotter S. (2008). Measurement of variability dynamics in cortical spike trains. J. Neurosci. Methods 169, 374–390. 10.1016/j.jneumeth.2007.10.013 18155774

[B43] Ocker G. K. Litwin-Kumar A. Doiron B. (2015). Self-organization of microcircuits in networks of spiking neurons with plastic synapses. PLoS Comput. Biol. 11:e1004458. 10.1371/journal.pcbi.1004458 26291697 PMC4546203

[B44] O'Connor D. H. Peron S. P. Huber D. Svoboda K. (2010). Neural activity in barrel cortex underlying vibrissa-based object localization in mice. Neuron 67, 1048–1061. 10.1016/j.neuron.2010.08.026 20869600

[B45] Ostojic S. (2014). Two types of asynchronous activity in networks of excitatory and inhibitory spiking neurons. Nat. Neurosci. 17, 594–600. 10.1038/nn.3658 24561997

[B46] Ostojic S. Brunel N. Hakim V. (2009). How connectivity, background activity, and synaptic properties shape the cross-correlation between spike trains. J. Neurosci. 29, 10234–10253. 10.1523/JNEUROSCI.1275-09.2009 19692598 PMC6665800

[B47] Petermann T. Thiagarajana T. C. Lebedev M. A. Nicolelis M. A. L. Chialvo D. R. Plenz D. (2009). Spontaneous cortical activity in awake monkeys composed of neuronal avalanches. Proc. Natl. Acad. Sci. U.S.A. 106, 15921–15926. 10.1073/pnas.0904089106 19717463 PMC2732708

[B48] Pillow J. W. (2009). Time-rescaling methods for the estimation and assessment of non-Poisson neural encoding models, in Advances in Neural Information Processing System 22, eds Bengio Y. Schuurmans D. Lafferty J. Williams C. Culotta A. (Cambridge: MIT Press), 1473–1481.

[B49] Roberts P. D. Bell C. C. (2000). Computational consequences of temporally asymmetric learning rules. II. Sensory image cancellation. J. Comput. Neurosci. 9, 67–83. 10.1023/A:1008938428112 10946993

[B50] Roxin A. Brunel N. Hansel D. Mongillo G. van Vreeswijk C. (2011). On the distribution of firing rates in networks of cortical neurons. J. Neurosci. 31, 16217–16226. 10.1523/JNEUROSCI.1677-11.2011 22072673 PMC6633220

[B51] Schneidman E. Berry M. J. II Sege R. Bialek W. (2006). Weak pairwise correlations imply strongly correlated network states in a neural population. Nature 440, 1007–1012. 10.1038/nature04701 16625187 PMC1785327

[B52] Schwindt P. Crill W. (1999). Mechanisms underlying burst and regular spiking evoked by dendritic depolarization in layer 5 cortical pyramidal neurons. J. Neurophysiol. 81, 1341–1354. 10085360 10.1152/jn.1999.81.3.1341

[B53] Shadlen M. N. Newsome W. T. (1998). The variable discharge of cortical neurons: implications for connectivity, computation, and information coding. J. Neurosci. 18, 3870–3896. 9570816 10.1523/JNEUROSCI.18-10-03870.1998 PMC6793166

[B54] Shafi M. Zhou Y. Quintana J. Chow C. Fuster J. Bodner M. (2007). Variability in neuronal activity in primate cortex during working memory tasks. Neuroscience 146, 1082–1108. 10.1016/j.neuroscience.2006.12.072 17418956

[B55] Softky W. R. Koch C. (1993). The highly irregular firing of cortical cells is inconsistent with temporal integration of random EPSPs. J. Neurosci. 13, 334–350. 8423479 10.1523/JNEUROSCI.13-01-00334.1993 PMC6576320

[B56] Song S. Abbott L. F. (2001). Cortical development and remapping through spike timing-dependent plasticity. Neuron 32, 339–350. 10.1016/S0896-6273(01)00451-2 11684002

[B57] Trousdale J. Hu Y. Shea-Brown E. Josić K. (2012). Impact of network structure and cellular response on spike time correlations. PLoS Comput. Biol. 8:e1002408. 10.1371/journal.pcbi.1002408 22457608 PMC3310711

[B58] Tuckwell H. C. (1988). Introduction to Theoretical Neurobiology, Vol. 2. Cambridge: Cambridge University Press.

[B59] Turrigiano G. (2011). Too many cooks? Intrinsic and synaptic homeostatic mechanisms in cortical circuit refinement. Annu. Rev. Neurosci. 34, 89–103. 10.1146/annurev-neuro-060909-153238 21438687

[B60] Turrigiano G. G, Nelson, S. B. (2004). Homeostatic plasticity in the developing nervous system. Nat. Rev. Neurosci. 5, 97–107. 10.1038/nrn1327 14735113

[B61] van Vreeswijk C. Sompolinsky H. (1996). Chaos in neuronal networks with balanced excitatory and inhibitory activity. Science 274, 1724–1726. 10.1126/science.274.5293.1724 8939866

[B62] van Vreeswijk C. Sompolinsky H. (1998). Chaotic balanced state in a model of cortical circuits. Neural Comput. 10, 1321–1371. 10.1162/089976698300017214 9698348

[B63] Watt A. J. Desai N. S. (2010). Homeostatic plasticity and STDP: keeping a neuron's cool in a fluctuating world. Front. Synaptic Neurosci. 2:5. 10.3389/fnsyn.2010.00005 21423491 PMC3059670

[B64] Widloski J. Fiete I. R. (2014). A model of grid cell development through spatial exploration and spike time-dependent plasticity. Neuron 83, 481–495. 10.1016/j.neuron.2014.06.018 25033187

