# Supplementary material for: Spike Pattern Structure Influences Synaptic Efficacy Variability under STDP and Synaptic Homeostasis. I: Spike Generating Models on Converging Motifs
Source: Front Comput Neurosci. 2016 Feb 23;10:14. doi: 10.3389/fncom.2016.00014 (PMC4763167; doi:10.3389/fncom.2016.00014)
Supplement: Supplementary file 1 [file DataSheet1.pdf]

Supplementary Material:
Spike Pattern Structure Influences Synaptic Efficacy Variability Under STDP and Synaptic Homeostasis. I: Spike Generating Models on Converging Motifs

Zedong Bi* and Changsong Zhou†

*Correspondence: zedong.bi@outlook.com

†Correspondence: cszhou@hkbu.edu.hk

1 The Influence of Auto-correlation Structure onto DiffV

1.1 The time scale of auto-correlation of Gamma process

In **Section 3.2** of the main text, we make the statement that for a Gamma process, the time scale of auto-correlation $\tau_{cross} \sim \frac{CV^2}{r_0}$ when $CV > 1$, and $\tau_{cross} \sim \frac{1}{4CV^2 \cdot r_0}$ when $CV < 1$, with r_0 being the rate of the Gamma process. Here, we make a simple proof.

Gamma process is a renewal process in which the inter-spike intervals are independently identically distributed as Gamma distribution:

$$\Gamma(x|\alpha, \beta) = \frac{1}{\Gamma(\alpha)\beta^\alpha} x^{\alpha-1} e^{-x/\beta} \tag{1}$$

We denote X_i to be the ith inter-spike interval, starting from the 0th spike. By defining moment generating function of Gamma distribution (http://mathworld.wolfram.com/gammadistribution.html)

$$Q(t) = \int_{-\infty}^{\infty} \Gamma(x|\alpha, \beta) e^{xt} \mathrm{d}x,$$

it is easy to prove that $X \equiv \sum_{i=1}^{n} X_i$ (which is the distribution of the interval between the 0th spike and the nth spike) follows $\Gamma(x|n\alpha, \beta)$, i.e.

$$X \equiv \sum_{i=1}^{n} X_i \sim \Gamma(x|n\alpha, \beta). \tag{2}$$

1) When $\alpha < 1$ (i.e. $CV = \frac{1}{\sqrt{\alpha}} > 1$), the auto-correlation of a Gamma process concentrates near zero, which describes the tendency that if the 0th spike is at t_0, then the next few spikes are also near t_0. From **eq.2**, we see that when $n = \frac{1}{\alpha}$, $X \sim \Gamma(x|1, \beta)$, which is an exponential distribution. This means that if we choose a spike every n spikes from the Gamma process, these chosen spikes will form a Poisson process, whose connected auto-correlation

is zero. This suggests that the time scale of the auto-correlation of the Gamma process should be no longer than the time scale of n inter-spike intervals, which means that

$$\tau_{cross} \sim \frac{n}{r_0} = \frac{1}{\alpha r_0} = \frac{CV^2}{r_0}$$

with r_0 being the firing rate.

2) When $\alpha > 1$ (i.e. $CV = \frac{1}{\sqrt{\alpha}} < 1$), the connected auto-correlation of a Gamma process is oscillating decaying. This is because that if the 0th spike is at t_0, then the next nth spike tend to be near $t_n = t_0 + \frac{n}{r_0}$, because of the regularity of the spike train. From **eq.2**, the standard deviation (s.d.) of the distribution of the time of the nth spike will be $\beta\sqrt{n\alpha}$. The oscillating behavior of the auto-correlation will be damped if the spike time distribution of adjacent spikes are overlapped, especially when the probability that a spike appears at $\frac{t_{n-1}+t_n}{2}$ is almost the same as the probability that a spike appears at t_{n-1} or t_n. This happens when the s.d. of the distribution is equal to half of the inter-spike interval:

$$\beta\sqrt{n\alpha} = \frac{1}{2r_0},$$

which gives (using $\beta = \frac{1}{\alpha r_0}$)

$$n = \frac{\alpha}{4}.$$

Therefore, the time scale of the auto-correlation of the Gamma process should be no longer than the time scale of n inter-spike intervals, which means that

$$\tau_{cross} \sim \frac{n}{r_0} = \frac{\alpha}{4r_0} = \frac{1}{4CV^2 r_0}.$$

1.2 The derivation of eqs. 21 and 22 in the main text

This subsection will derive **eqs. 21** and **22** in the main text.

Suppose a spike of the central neuron is at time t_i, let's consider the potentiation value caused by it in the ath non-central neuron, which is

$$\sum_j \Delta w_{a,p}(t_i, t_{j|a,i,p}) = A_p \sum_{j=1}^{\infty} \exp(-\frac{t_i - \tau_{delay} - t_{j|a,i,p}}{\tau_{STDP}}).$$

Under strict regularity, $t_{j|a,i,p} = t_{1|a,i,p} - (j-1)\Delta t$, with Δt being the inter-spike interval. If we define $t_0 = t_i - \tau_{delay} - t_{1|a,i,p}$, then the equation above becomes

$$\sum_j \Delta w_{a,p}(t_i, t_{j|a,i,p}) = A_p \exp(-\frac{t_0}{\tau_{STDP}})[1 + \exp(-\frac{\Delta t}{\tau_{STDP}}) + \exp(-\frac{2\Delta t}{\tau_{STDP}}) + \cdots]$$

$$= \frac{A_p \exp(-\frac{t_0}{\tau_{STDP}})}{1 - \exp(-\frac{\Delta t}{\tau_{STDP}})}.$$

Under strict regularity, t_0 is uniformly distributed within $[-\Delta t, 0]$, and it is easy to show that if the size of the

converging motif is sufficiently large,

$$\mathrm{Var}_a(\sum_j \Delta w_{a,p}(t_i,t_j)) = A_p^2 \frac{1}{(1-\exp(-\frac{\Delta t}{\tau_{STDP}}))^2}[\frac{\tau_{STDP}}{2\Delta t}(1-\exp(-\frac{2\Delta t}{\tau_{STDP}})) - \frac{\tau_{STDP}^2}{\Delta t^2}(1-\exp(-\frac{\Delta t}{\tau_{STDP}}))^2]$$

$$= A_p^2 \frac{\tau_{STDP}}{2\Delta t}[\frac{1+\exp(-\frac{\Delta t}{\tau_{STDP}})}{1-\exp(-\frac{\Delta t}{\tau_{STDP}})} - \frac{2\tau_{STDP}}{\Delta t}].$$

After considering the depression process, we have

$$d_{reg} = \sum_{k=p,d} \mathrm{Var}_a(\sum_j \Delta w_{a,k}(t_i,t_j)) = (A_d^2 + A_p^2)\frac{\tau_{STDP}}{2\Delta t}[\frac{1+\exp(-\frac{\Delta t}{\tau_{STDP}})}{1-\exp(-\frac{\Delta t}{\tau_{STDP}})} - \frac{2\tau_{STDP}}{\Delta t}], \tag{3}$$

which is **eq.21** in the main text.

Under Poisson processes, the ocurrences of spikes in any two small time bins of length $\mathrm{d}t$ are independent, and the variance of the number of spikes within a time bin is $r_0\mathrm{d}t$, with r_0 being the firing rate. As $\Delta w_{a,p}(t_i,t_j) = A_p \exp(-\frac{t}{\tau_{STDP}})$ (with $t = t_i - \tau_{delay} - t_j$), we have

$$\mathrm{Var}_a(\sum_j \Delta w_{a,p}(t_i,t_j)) = A_p^2 \int_0^\infty \exp(-\frac{2t}{\tau_{STDP}}) r_0 \mathrm{d}t$$

$$= A_p^2 \frac{\tau_{STDP}}{2\Delta t}.$$

Therefore,

$$d_{Poi} = \sum_{k=p,d} \mathrm{Var}_a(\sum_j \Delta w_{a,k}(t_i,t_j)) = (A_d^2 + A_p^2)\frac{\tau_{STDP}}{2\Delta t}, \tag{4}$$

which is **eq.22** in the main text.

From **eq.3** and **eq.4**, we know that to prove $d_{reg} < d_{Poi}$ is equivalent to prove $\frac{1+\exp(-\frac{\Delta t}{\tau_{STDP}})}{1-\exp(-\frac{\Delta t}{\tau_{STDP}})} - \frac{2\tau_{STDP}}{\Delta t} < 1$, which is then equivalent to prove

$$f(x) = (x+1)\exp(-x) < 1 \quad \text{for } x > 0.$$

This statement above is easy to prove after noting that $f(0) = 1$ and $f'(x) < 0$ for $x > 0$.

2 The Interaction of Auto-correlation Structure and Heterogeneity of Rates

In this section, we will consider the case when the spike trains are stationary processes (so that the trial averaged firing rates do not change with time), and different non-central neurons have different firing rates. In this case, the trial averaged synaptic change rate is

$$\frac{\mathrm{dE}_T(\Delta w_a)}{\mathrm{d}t} = r_0 r_a (A_p - A_d)\int_0^\infty \exp(-\frac{\tau}{\tau_{STDP}})\mathrm{d}\tau,$$

with r_0 being the firing rate of the central neuron, r_a being the firing rate of the ath non-central neuron. Therefore,

$$\mathrm{DriftV} \propto \frac{\mathrm{dVar}_a(\mathrm{E}_T(\Delta w_a))}{\mathrm{d}t} = [r_0(A_p - A_d)\int_0^{\infty} \exp(-\frac{\tau}{\tau_{STDP}})\mathrm{d}\tau]^2 \mathrm{Var}_a(r_a).$$

We see that auto-correlation structure does not enter the formula above, and therefore does not influence DriftV in this case. To intuitively understand this, we denote the interval between a central spike t_i and a non-central spike t_j to be $\Delta t = t_i - t_j$; and note that as we suppose that the central and non-central spike trains are independent, the distribution of Δt should be always uniform within $(0, \infty)$ in the long run, *independent* on the auto-correlation structure of their spike trains. This causes the same potentiation and depression under STDP even when the auto-correlation structure changes.

Next, we want to understand how the auto-correlation structure may change DiffV under heterogeneity of rates. To do this, we generate spike trains as Gamma processes, with the firing rate of the central neuron being kept at $r_0 = 20$Hz, and the firing rates of the non-central neuron following lognormal distribution with mean r_0 (by combining Model Auto & Model Long Tail in **Materials and Methods** in the main text). The coefficient of variance (CV) of the spike trains of the central and non-central neurons are the same. We set $A_p = A_d$, so that $\mathrm{DrV} = 0$ and $\mathrm{DiV} \approx \mathrm{E}_T(\mathrm{Var}_a(\Delta w_a))$ (see **eq.5** in the main text). We then compared $\mathrm{E}_T(\mathrm{Var}_a(\Delta w_a))$ in this model with that under *homogeneity* of rate introduced in **Section 3.2** of the main text. We find that heterogeneity of rates hardly changes DiffV when spike trains are bursty, but effectively discounts the increase of DiffV caused by regularity (**Supplementary Figure 1A**).

To understand the reason of this phenomenon, suppose that the smallest firing rate of the non-central neurons is r_{min} and the largest is r_{max}, and we can divide the interval $[r_{min}, r_{max}]$ into many bins of length 2ϵ, with ϵ being a small value. We denote the sth bin to be $\mathcal{A}_s = (r_s - \epsilon, r_s + \epsilon)$, with r_s being the middle value of this bin. If the converging motif is very large, then there will be many non-central neurons whose firing rates lie within each bin. After implementing the theorem of total variance (see **eq.2** in the main text), we have that

$$\mathrm{Var}_a(\Delta w_a) = \mathrm{E}_{\mathcal{A}_s}(\mathrm{Var}_{r_t \in \mathcal{A}_s}(\Delta w_t)) + \mathrm{Var}_{\mathcal{A}_s}(\mathrm{E}_{r_t \in \mathcal{A}_s}(\Delta w_t)). \tag{5}$$

As $\mathrm{DriftV} = 0$ here, we have $\mathrm{Var}_{\mathcal{A}_s}(\mathrm{E}_{r_t \in \mathcal{A}_s}(\Delta w_t)) = 0$, so that

$$\mathrm{E}_T(\mathrm{Var}_a(\Delta w_a)) = \mathrm{E}_T[\mathrm{E}_{\mathcal{A}_s}(\mathrm{Var}_{r_t \in \mathcal{A}_s}(\Delta w_t))] = \mathrm{E}_{\mathcal{A}_s}[\mathrm{E}_T(\mathrm{Var}_{r_t \in \mathcal{A}_s}(\Delta w_t))] \tag{6}$$

which means that the value of $\mathrm{E}_T(\mathrm{Var}_a(\Delta w_a))$ can be understood by investigating how $\mathrm{E}_T(\mathrm{Var}_{r_t \in \mathcal{A}_s}(\Delta w_t))$ changes with r_s. As $\mathcal{A}_s$ is a small bin near r_s, we can suppose that all the values within $\mathcal{A}_s$ can be approximately by r_s, so that

$$\mathrm{E}_T(\mathrm{Var}_{r_t \in \mathcal{A}_s}(\Delta w_t)) \approx \mathrm{E}_T(\mathrm{Var}_{r_t = r_s}(\Delta w_t)), \tag{7}$$

in which $\mathrm{E}_T(\mathrm{Var}_{r_t = r_s}(\Delta w_t))$ represents the efficacy variability when the firing rates of all non-central neurons are approximated to be r_s. Using simulations, we found that $\mathrm{E}_T(\mathrm{Var}_{r_t = r_s}(\Delta w_t))$ changes with r_s in the following features (**Supplementary Figure 1B**):

1) $\mathrm{E}_T(\mathrm{Var}_{r_t = r_s}(\Delta w_t))$ tends to increase with r_s.

2) When $CV \ll 1$ (i.e. the spike trains are regular), $\mathrm{E}_T(\mathrm{Var}_{r_t = r_s}(\Delta w_t))$ tends to sharply peak at $r_s = r_0$, and may also peak at $2r_0$, $\frac{1}{2}r_0$ etc.

To understand the first point above, note that if we regard the synaptic changes under STDP as random walks,

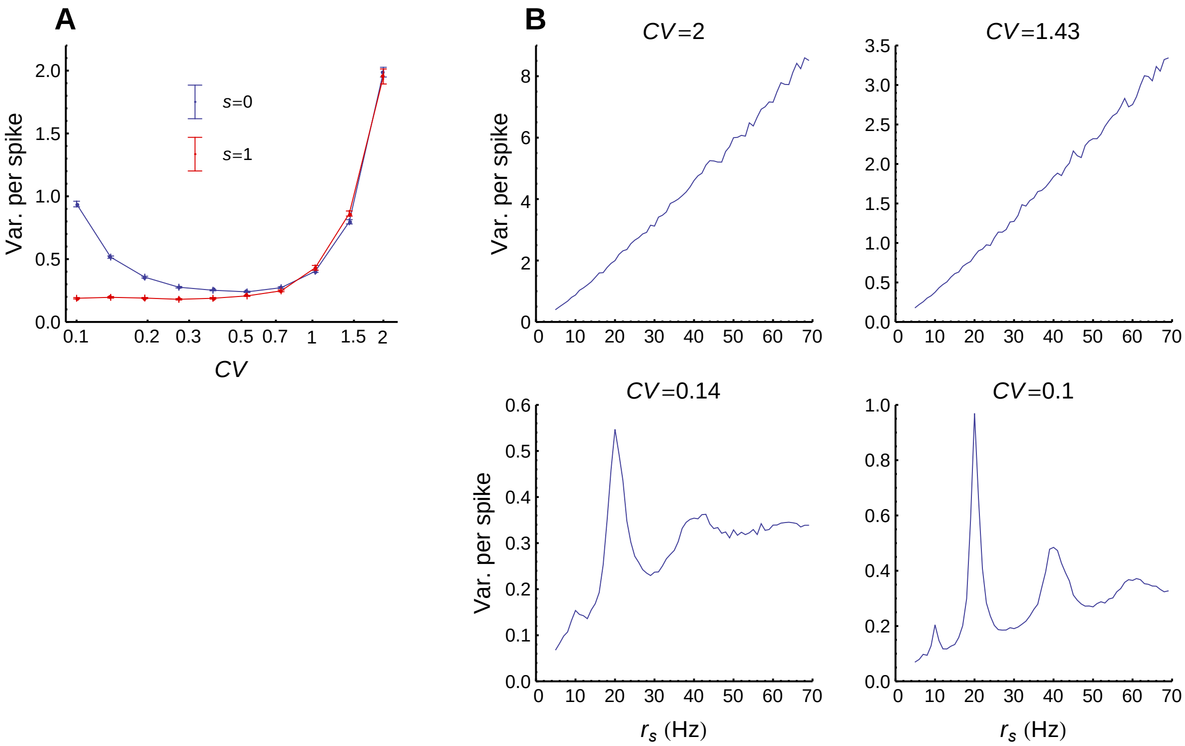

Figure 1: **How the auto-correlation structure changes DiffV under heterogeneity of rates.** (**A**) Variance per spike ($\mathrm{E}_T(\mathrm{Var}_a(\Delta w_a))/\bar{N}_0$, with $\bar{N}_0$ being the expectation of the spike number of the central neuron) as a function of CV. We see that heterogeneity of rates does not significantly influence DiffV (as DriftV $= 0$ here, $\mathrm{E}_T(\mathrm{Var}_a(\Delta w_a)) = \mathrm{DiffV}$) when the spikes are bursty, but removes the increase of DiffV caused by strong regularity. The spike trains are Gamma processes, with the firing rate of the central neuron being kept at $r_0 = 20$Hz. The firing rates of the non-central neurons follow lognormal distributions with mean r_0 and shape parameter s (by combining Model Auto & Model Long Tail in **Materials and Methods** in the main text). The coefficient of variance (CV) of the spike trains of the central and non-central neurons are the same. We set $A_p = A_d$, so that DriftV $= 0$. Error bars represent s.e.m. (**B**) Variance per spike as a function of r_s, if the firing rate of the central neuron is r_0 and the firing rates of all the non-central neurons are r_s. Error bars are not shown for clarity. In **A-B**, the size of the converging motif, the parameters for STDP as well as the simulation time and trials are the same as in **Figure 4** in the main text.

then the synaptic change caused by a spike of a non-central neuron can be regarded as a step of the random walk. If the firing rates of the non-central neurons are large, then the step number of the random walk on a synapse per unit time will be large, thereby increasing the diffusion strength. In our model, we found $\mathrm{E}_T(\mathrm{Var}_{r_t=r_s}(\Delta w_t))\tilde{\propto} r_s$ (with $\tilde{\propto}$ representing "approximately proportional to") when $CV > 1$ (**Supplementary Figure 1B, upper panels**). From **eq.6** and **7**, this implies that $\mathrm{E}_T(\mathrm{Var}_a(\Delta w_a))\tilde{\propto}\mathrm{E}_s(r_s)$. This means that DiffV will not significantly change as long as the mean firing rate of the non-central neurons conserves, which explains why $\mathrm{E}_T(\mathrm{Var}_a(\Delta w_a))$ does not change significantly when the firing rates of the non-central neurons become widely distributed (**Supplementary Figure 1A**).

The second point above can be understood using the mechanism transient cross-correlation introduced in **Section 3.2.2** in the main text. When spike trains are strongly regular, if $N_1 r_s = N_2 r_0$ (with N_1 and N_2 being two positive integers with no common divisor larger than 1), then $\mathrm{E}_T(\mathrm{Var}_{r_t=r_s}(\Delta w_t))$ can be enlarged by the correlation between the synaptic updatings caused by adjacent central spikes. DiffV under strong regularity has a sharp peak at $r_s = r_0$ (**Supplementary Figure 1B lower panels**). So if the firing rates of all the non-central neurons are r_0, then DiffV will be large under strong regularity. However, if the firing rates of the non-central neurons are heterogeneous, then many r_s will be at non-peak values in **Supplementary Figure 1B lower panels**. From **eq.6** and **7**, this implies that $\mathrm{E}_T(\mathrm{Var}_a(\Delta w_a))$ will be decreased. Therefore, heterogeneity of rates decreases DiffV in regular spike trains (see **Supplementary Figure 1A**) by destroying the transient cross-correlation between the central neuron and the non-central neurons.

3 The Influence of Synchronous Firing onto DiffV

3.1 The Influence of Synchronous Firing onto d

This subsection will derive **eqs. 27-29** in the main text.

Because of $\tau_{cross} \ll \tau_{STDP}$, we neglect the contribution to the efficacy variance by the random displacements of spike times in a synchronous event, therefore the efficacy variance is only contributed by the difference of spike numbers of different neurons in a synchronous event. In this case, suppose an central spike at time t_i, and a synchronous event $\mathcal{S}$ happening during (t_1, t_2) with $t_i - \tau_{delay} < t_1 < t_2$, then we approximate the STDP updating caused by the central spike and any non-central spike in $\mathcal{S}$ using the time difference between t_i and the middle time $\frac{t_1+t_2}{2}$ of $\mathcal{S}$, i.e.

$$\Delta w_a(t_i, t_{j\in\mathcal{S}}) \approx -A_d \exp[-\frac{(t_1+t_2)/2 - (t_i - \tau_{delay})}{\tau_{STDP}}] \tag{8}$$

Thus, the efficacy variance caused by pairing the central spike and the synchronous event $\mathcal{S}$ is

$$\mathrm{Var}_a(\sum_j \Delta w_a(t_i, t_{j\in\mathcal{S}})) \approx \mathrm{Var}_a(N_a) A_d^2 \exp[-\frac{2((t_1+t_2)/2 - (t_i - \tau_{delay}))}{\tau_{STDP}}]$$

with N_a being the spike number of the ath non-central neuron during $\mathcal{S}$. This approximation will be used in our following calculations.

$\tau_{cross} \leq \tau_{delay}$:

Given an central spike at time t_i, we denote the synchronous event that the central spike belongs to as $\mathcal{S}_0$. When $\tau_{cross} \leq \tau_{delay}$, the interaction of $\mathcal{S}_0$ and t_i can only depress the synapses. After considering the axonal delay, the

duration of the interval between t_i and the middle time of $\mathcal{S}_0$ is uniformly distributed within $(\tau_{delay} - \frac{\tau_{cross}}{2}, \tau_{delay} + \frac{\tau_{cross}}{2})$, therefore, its contribution to $\mathrm{E}_T(\mathrm{Var}(\sum_j \Delta w_{a,d}(t_i, t_j)))$ is

$$\mathrm{E}_T(\mathrm{Var}_a(\sum_{j\in\mathcal{S}_0} \Delta w_{a,d}(t_i, t_j))) = \mathrm{Var}_a(N_a) A_d^2 \int_{\tau_{delay}-\frac{\tau_{cross}}{2}}^{\tau_{delay}+\frac{\tau_{cross}}{2}} \frac{1}{\tau_{cross}} \exp(-\frac{2x}{\tau_{STDP}})\mathrm{d}x$$

$$\approx \mathrm{Var}_a(N_a) A_d^2 \exp(-\frac{2\tau_{delay}}{\tau_{STDP}}). \tag{9}$$

We again use the fact that $\tau_{cross} \ll \tau_{STDP}$ in the approximation above.

In our model, the occurrence of synchronous events is a Poisson process of rate r_0/p. For simplicity, we set $\tau_{cross} \to 0$ for the synchronous events other than $\mathcal{S}_0$. In this case,

$$\mathrm{E}_T(\mathrm{Var}_a(\sum_{j\notin\mathcal{S}_0} \Delta w_{a,d}(t_i, t_j))) = \mathrm{Var}_a(N_a) A_d^2 \int_0^{\infty} \exp(\frac{-2x}{\tau_{STDP}}) r_0/p\mathrm{d}x]$$

$$= \mathrm{Var}_a(N_a(p)) A_d^2 \frac{r_0 \tau_{STDP}}{2p} \tag{10}$$

Similarly,

$$\mathrm{E}_T(\mathrm{Var}_a(\sum_{j\notin\mathcal{S}_0} \Delta w_{a,p}(t_i, t_j))) = \mathrm{Var}_a(N_a(p)) A_p^2 \frac{r_0 \tau_{STDP}}{2p} \tag{11}$$

Therefore, combining **eqs.9-11**, we have that for $\tau_{cross} \leq \tau_{delay}$,

$$d \approx \mathrm{Var}_a(N_a)[A_d^2 \exp(-\frac{2\tau_{delay}}{\tau_{STDP}}) + \frac{r_0 \tau_{STDP}}{2p}(A_d^2 + A_p^2)] \tag{12}$$

which is **eq.27** in the main text. When p is large, $\mathrm{Var}_a(N_a(p)) \approx pCV_{SpikeNum}^2$ (Cox (1962); Tuckwell (1988); Nawrot et al. (2008)), so that

$$d \approx pCV_{SpikeNum}^2 A_d^2 \exp(-\frac{2\tau_{delay}}{\tau_{STDP}}) + CV_{SpikeNum}^2 \frac{r_0 \tau_{STDP}}{2}(A_d^2 + A_p^2) \tag{13}$$

which is **eq.28** in the main text.

$\tau_{cross} > \tau_{delay}$:

Suppose that the synchronous event $\mathcal{S}_0$ that the central spike t_i belongs to lasts during (t_1, t_2), then when $\tau_{cross} > \tau_{delay}$, there are two possibilities:

1) If $t_i < t_1 + \tau_{delay}$, all the non-central spikes come at the central neuron after t_i, thereby depressing the synapses.

2) If $t_i > t_1 + \tau_{delay}$, then it is possible that some non-central spikes come at the central neuron earlier than t_i, which potentiate the corresponding synapses, while the other non-central spikes come at the central neuron later than t_i, which depress the corresponding synapses.

After considering these two possibilities, we can write the contribution of $\mathcal{S}_0$ to $\mathrm{E}_T(\mathrm{Var}_a(\sum_j \Delta w_{a,d}(t_i, t_j)))$ as

$$\mathrm{E}_T(\mathrm{Var}_a(\sum_{j\in\mathcal{S}_0} \Delta w_{a,d}(t_i, t_j)))$$

$$= \mathrm{Var}_a(N_a)A_d^2\int_{\frac{\tau_{cross}}{2}}^{\tau_{delay}+\frac{\tau_{cross}}{2}}\frac{1}{\tau_{cross}}\exp(-\frac{2x}{\tau_{STDP}})\mathrm{d}x+\int_0^{\tau_{cross}-\tau_{delay}}\frac{1}{\tau_{cross}}\mathrm{Var}_a(N_a,\frac{\tau_{cross}-x}{\tau_{cross}})A_d^2\exp(-\frac{2(\tau_{cross}-x)/2}{\tau_{STDP}})\mathrm{d}x$$

$$\approx \mathrm{Var}_a(N_a)A_d^2\frac{\tau_{delay}}{\tau_{cross}}\exp(-\frac{\tau_{cross}+\tau_{delay}}{\tau_{STDP}})+\frac{1}{\tau_{cross}}A_d^2\exp(-\frac{\tau_{cross}+\tau_{delay}}{2\tau_{STDP}})\cdot\int_0^{\tau_{cross}-\tau_{delay}}\mathrm{Var}_a(N_a,\frac{\tau_{cross}-x}{\tau_{cross}})\mathrm{d}x. \tag{14}$$

And we again use the fact that $\tau_{cross} \ll \tau_{STDP}$ in the approximation above. In this equation, $\mathrm{Var}_a(N_a, x)$ means that if M_a of the N_a spikes of the ath non-central neuron are chosen (which means that they lie within an interval of duration $\Delta\tau$ with $\Delta\tau/\tau_{cross} = x$ in a synchronous event), then $\mathrm{Var}_a(N_a, x) = \mathrm{Var}_a(M_a)$. In this model (Model Sync in the main text), the time of each of the N_a spikes is independently and uniformly distributed within the synchronous event $\mathcal{S}_0$, therefore each of them has probability x to be chosen.

Similarly, the contribution of $\mathcal{S}_0$ to $\mathrm{E}_T(\mathrm{Var}_a(\sum_j \Delta w_{a,p}(t_i, t_j)))$ is

$$\mathrm{E}_T(\mathrm{Var}_a(\sum_{j\in\mathcal{S}_0}\Delta w_{a,p}(t_i,t_j))) = \int_0^{\tau_{cross}-\tau_{delay}}\frac{1}{\tau_{cross}}\mathrm{Var}_a(N_a,\frac{x}{\tau_{cross}})A_p^2\exp(-\frac{2x/2}{\tau_{STDP}})\mathrm{d}x$$

$$\approx \frac{1}{\tau_{cross}}A_p^2\exp(-\frac{\tau_{cross}-\tau_{delay}}{2\tau_{STDP}})\cdot\int_0^{\tau_{cross}-\tau_{delay}}\mathrm{Var}_a(N_a,\frac{x}{\tau_{cross}})\mathrm{d}x. \tag{15}$$

And the same as the $\tau_{delay} < \tau_{cross}$ case (**eqs.10** and **11**), we approximate the contribution of the synchronous events other than $\mathcal{S}_0$ as

$$\mathrm{E}_T(\mathrm{Var}_a(\sum_{j\notin\mathcal{S}_0}\Delta w_{a,d}(t_i,t_j))) + \mathrm{E}_T(\mathrm{Var}_a(\sum_{j\notin\mathcal{S}_0}\Delta w_{a,p}(t_i,t_j))) = \mathrm{Var}_a(N_a(p))\frac{r_0\tau_{STDP}}{2p}(A_d^2+A_p^2). \tag{16}$$

Eqs.14-16 together give the value of $\mathrm{E}_T(\mathrm{Var}_a(\sum_j \Delta w_{a,d}(t_i, t_j))) + \mathrm{E}_T(\mathrm{Var}_a(\sum_j \Delta w_{a,p}(t_i, t_j)))$.

Now we calculate the value of $\mathrm{Var}_a(N_a, x)$ in **eqs.14** and **15**. Suppose that there are N non-central neurons, and the number of chosen spikes of the ath non-central neuron is M_a, then

$$\mathrm{Var}_a(N_a,x) = \frac{M_1^2+M_2^2+\cdots+M_N^2}{N} - (\frac{M_1+M_2+\cdots+M_N}{N})^2$$

Here M_a follows binomial distribution, whose mean is $N_a x$ and variance is $N_a x(1-x)$. Therefore,

$$\mathrm{Var}_a(N_a,x) = \int \mathrm{d}N_a q(N_a)(N_a x(1-x)+N_a^2x^2) - (\int \mathrm{d}N_a q(N_a)N_a)^2\cdot x^2$$

$$= px(1-x) + \mathrm{Var}_a(N_a)x^2$$

$$\approx px(1-x) + pCV_{SpikeNum}^2 x^2 \tag{17}$$

with $q(N_a)$ being the distribution of N_a, and we also use the fact that $\mathrm{Var}_a(N_a) \approx pCV^2_{SpikeNum}$ if p is large and $\int \mathrm{d}N_a q(N_a)N_a = p$. Therefore, we know that in **eq.14**,

$$\int_0^{\tau_{cross}-\tau_{delay}}\mathrm{Var}_a(N_a,\frac{\tau_{cross}-x}{\tau_{cross}})\mathrm{d}x$$

$$= \tau_{cross}p\frac{1}{2}(1-(\frac{\tau_{delay}}{\tau_{cross}})^2) + \tau_{cross}p(CV_{SpikeNum}^2-1)\frac{1}{3}(1-(\frac{\tau_{delay}}{\tau_{cross}})^3), \tag{18}$$

and in **eq.15**,

$$\int_0^{\tau_{cross}-\tau_{delay}} \mathrm{Var}_a(N_a, \frac{x}{\tau_{cross}})\mathrm{d}x$$

$$= \tau_{cross} p \frac{1}{2}((1-\frac{\tau_{delay}}{\tau_{cross}})^2) + \tau_{cross} p (CV_{SpikeNum}^2 - 1)\frac{1}{3}((1-\frac{\tau_{delay}}{\tau_{cross}})^3). \tag{19}$$

Combining **eqs.14-19**, we have

$$d \approx p[CV^2 A_d^2 \frac{\tau_{delay}}{\tau_{cross}} \exp(-\frac{\tau_{cross}+\tau_{delay}}{\tau_{STDP}}) + A_d^2 \exp(-\frac{\tau_{cross}+\tau_{delay}}{2\tau_{STDP}}) \cdot \mathcal{A}$$

$$+A_p^2 \exp(-\frac{\tau_{cross}-\tau_{delay}}{2\tau_{STDP}}) \cdot \mathcal{B}] + CV_{SpikeNum}^2 \frac{r_0 \tau_{STDP}}{2}(A_d^2 + A_p^2), \tag{20}$$

with

$$\mathcal{A} = (\frac{1}{6} + \frac{1}{3}CV_{SpikeNum}^2) - \frac{1}{2}(\frac{\tau_{delay}}{\tau_{cross}})^2 - \frac{1}{3}(CV_{SpikeNum}^2 - 1)(\frac{\tau_{delay}}{\tau_{cross}})^3$$

$$\mathcal{B} = (1-\frac{\tau_{delay}}{\tau_{cross}})^2(\frac{1}{2} + \frac{1}{3}(CV_{SpikeNum}^2 - 1)(1-\frac{\tau_{delay}}{\tau_{cross}})),$$

which gives **eq.29** in the main text.

3.2 The Influence of Synchronous Firing onto c_{II}

In this section, we will try to understand the influence of synchronous firing onto c_{II} (**Supplementary Figure 2 A4, B4, C4**). When p is large, the change of c_{II} with p is small (**Supplementary Figure 2 A4, B4, C4**), which suggests that compared to the other factors, c_{II} contributes little to the increase of DiffV with p. When $\tau_{cross} \leq \tau_{delay}$, the change of c_{II} with $CV_{SpikeNum}$ is not strong; and when $\tau_{cross} > \tau_{delay}$, c_{II} tends to decrease with $CV_{SpikeNum}$ (**Supplementary Figure 2 A4, B4, C4**), which negatively contributes to the increase of DiffV with $CV_{SpikeNum}$. These facts suggest that c_{II} is not an important factor to understand the change of DiffV under synchronous firing. In this section, we will discuss the influence of synchronous firing onto c_{II} only for completeness. Readers may skip this subsection when reading for the first time.

From **Supplementary Figure 2 A4, B4** and **C4**, we can see that c_{II} is usually smaller than 1, which, by definition (**eq.15** in the main text), means that ρ_{PD} is usually negative. The reason for this negative correlation is the same as that shown in **Figure 4D inset** in the main text: if the ath non-central neuron fires more (less) spikes, then both the potentiation and depression imposed on the ath synapses tend to be strong (weak). Therefore, the total potentiation and depression value tend to be negatively correlated through the heterogeneity of spike numbers of the non-central neurons. However, c_{II} increases with the decrease of $CV_{SpikeNum}$, and becomes larger than 1 when $CV_{SpikeNum}$ is small enough. By definition (**eq.15** in the main text), $c_{II} > 1$ means $\rho_{PD} > 0$. We will try to understand this phenomenon in the following discussion.

Suppose a central spike t_i and the synchronous event $\mathcal{S}_0$ that t_i belongs to, let us consider a synchronous event $\mathcal{S}_-$ that occurs before $\mathcal{S}_0$, and a synchronous event $\mathcal{S}_+$ that occurs after $\mathcal{S}_0$. Then under the case that the inter-event interval p/r_0 is large enough (which is realized when p is large) and the occurrence of synchronous events is not too bursty, it is likely that

$$t_i - \tau_{delay} > t_j, \text{ for all } t_j \in \mathcal{S}_-$$

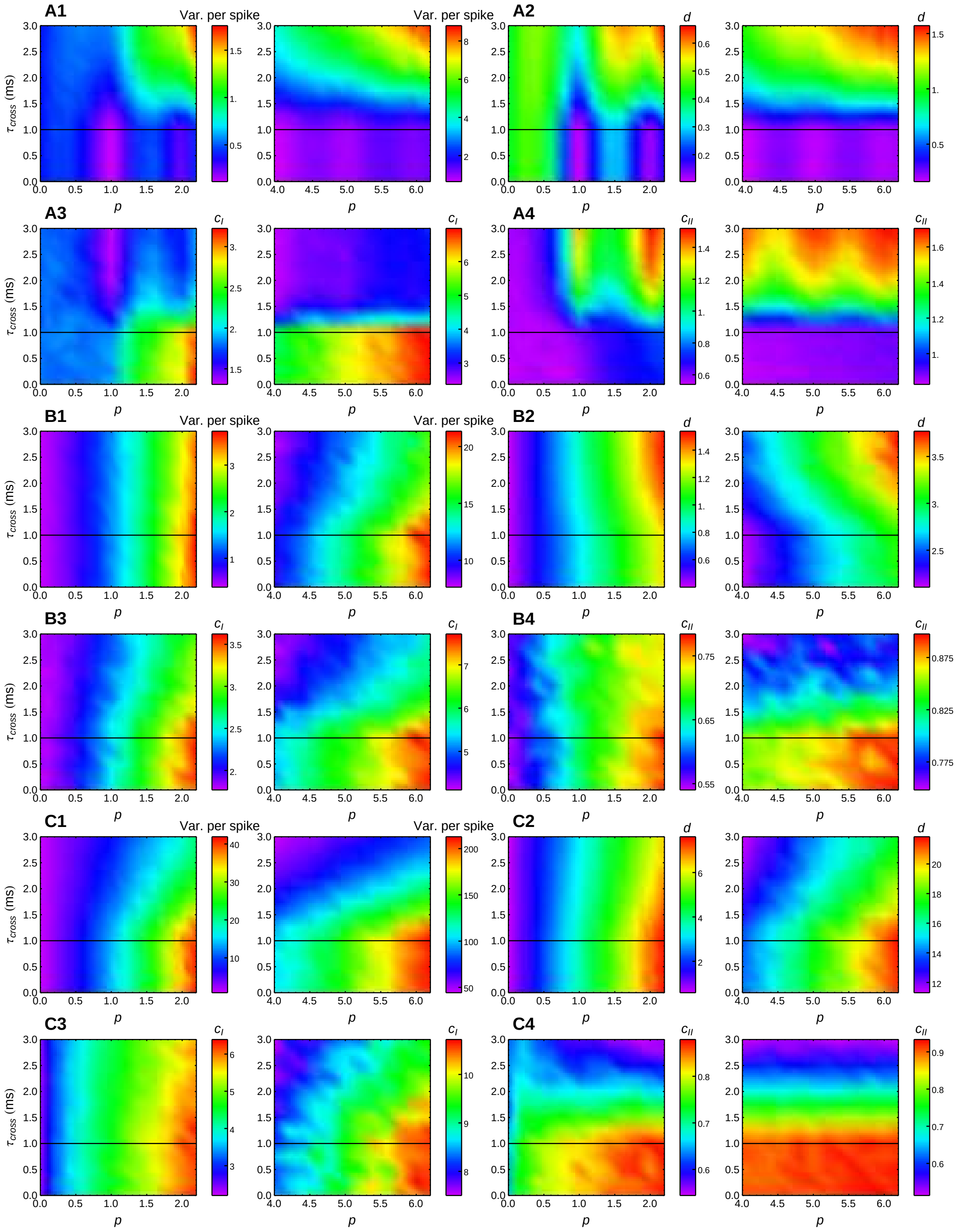

Figure 2: **How the efficacy variability, d, c_I and c_{II} change with p and $CV_{SpikeNum}$.** (**A1**) Variance per spike as a function of p when $CV_{SpikeNum} = 0.1$. The horizontal black line represents the axonal delay $\tau_{delay} = 1$ms. (**A2-A4**) The same as **A1**, but for d, c_I and c_{II}. Spike trains are generated according to Model Sync in the main text. (**B1-B4**) The same as **A1-A4**, but when $CV_{SpikeNum} = 0.71$. (**C1-C4**) The same as **A1-A4**, but when $CV_{SpikeNum} = 2$. In **A1-C4**, the size of the converging motif, the parameters for STDP as well as the simulation time and trials are the same as in **Figure 4** in the main text.

$$t_i - \tau_{delay} < t_j, \text{ for all } t_j \in \mathcal{S}_+$$

If $\tau_{cross} \ll \tau_{STDP}$, then the synaptic updating caused by pairing t_i with non-central spikes in $\mathcal{S}_+$ or $\mathcal{S}_-$ will not have much difference between when $\tau_{cross} \leq \tau_{delay}$ and when $\tau_{cross} > \tau_{delay}$, since in both cases the synaptic updatings can be approximated by

$$\Delta w_a(t_i, t_{j\in\mathcal{S}_-}) \approx A_p \exp(-\frac{(t_i - \tau_{delay}) - t_{\mathcal{S}_-}}{\tau_{STDP}}),$$

and

$$\Delta w_a(t_i, t_{j\in\mathcal{S}_+}) \approx -A_d \exp(-\frac{t_{\mathcal{S}_+} - (t_i - \tau_{delay})}{\tau_{STDP}}),$$

with $t_{\mathcal{S}_-}$ and $t_{\mathcal{S}_+}$ respectively being the middle time of $\mathcal{S}_-$ and $\mathcal{S}_+$. However, the interactions of t_i with non-central spikes in $\mathcal{S}_0$ is quite different when $\tau_{cross} \leq \tau_{delay}$ with those when $\tau_{cross} > \tau_{delay}$. When $\tau_{cross} \leq \tau_{delay}$, all the non-central spikes in $\mathcal{S}_0$ depress the synapses after pairing with t_i (i.e. $\Delta w_a(t_i, t_{j\in\mathcal{S}_0}) < 0$), but when $\tau_{cross} > \tau_{delay}$, some non-central spikes may potentiate the synapses (i.e. $\Delta w_a(t_i, t_{j\in\mathcal{S}_0}) > 0$). From **Supplementary Figure 2 A4, B4 and C4**, c_{II} decreases with $CV_{SpikeNum}$ when $\tau_{cross} > \tau_{delay}$, but does not significantly change when $\tau_{cross} \leq \tau_{delay}$, which suggests that the possibility that $\Delta w(t_i, t_{j\in\mathcal{S}_0}) > 0$ under $\tau_{cross} > \tau_{delay}$ is a key point to understand the decrease of ρ_{PD} with $CV_{SpikeNum}$.

Intuitively, during a synchronous event $(0, \tau_{cross})$ (with $\tau_{cross} > \tau_{delay}$), the non-central spikes that are emitted during $(0, t_i - \tau_{delay})$ will potentiate the synapses, and the non-central spikes that are emitted during $(t_i - \tau_{delay}, \tau_{cross})$ will depress the synapses. If $CV_{SpikeNum}$ is very small, then the ath neuron can fire almost exact p spikes during a synchronous event. In this case, if these p spikes are within $(0, t_i - \tau_{delay})$, then all of them will potentiate the ath synapses, so that $\Delta w_{a,p}$ is large and $\Delta w_{a,d} = 0$; but if they are all within $(t_i - \tau_{delay}, \tau_{cross})$, then $\Delta w_{a,p} = 0$ and $\Delta w_{a,d}$ will be very negative. This seems to be a possible mechanism that positively correlate $\Delta w_{a,p}$ and $\Delta w_{a,d}$ when $CV_{SpikeNum}$ is small and $\tau_{cross} > \tau_{delay}$ (**Supplementary Figure 2 A4**). Mathematically, it is complicated to analytically calculate ρ_{PD}, but we can implement this idea by using the following simple model, thereby understand the increase of ρ_{PD} with the decrease of $CV_{SpikeNum}$ under $\tau_{cross} > \tau_{delay}$.

Let's suppose $\mathcal{S}_0$ happens during $(0, \tau_{cross})$ with $\tau_{cross} > \tau_{delay}$, and the spike of the central neuron is at $t_i - \tau_{delay} = x\tau_{cross}$ with $0 < x < 1$, so that x portion of the spikes of the non-central neuron potentiate the synapses, while $1 - x$ portion depress the synapses. If $\tau_{cross} \ll \tau_{STDP}$, then we can suppose that all the spikes within $(0, x\tau_{cross})$ will potentiate the synapses by $y_p \approx A_p \exp(-\frac{x\tau_{cross}}{2\tau_{STDP}})$, and all the spikes within $(x\tau_{cross}, \tau_{cross})$ will depress the synapses by $-y_d \approx -A_d \exp(-\frac{\tau_{cross}(1-x)}{2\tau_{STDP}})$. The correlation between the potentiation and depression values in this model will be calculated in **Section 4.1** below (**eq.22**), the result is

$$\rho_{PD} = \frac{\int \mathrm{d}N_a q(N_a)\mathrm{Var}(M_a|N_a, x) - x(1-x)\mathrm{Var}_a(N_a)}{\sqrt{[\int \mathrm{d}N_a q(N_a)\mathrm{Var}(M_a|N_a, x) + x^2\mathrm{Var}_a(N_a)] \cdot [\int \mathrm{d}N_a q(N_a)\mathrm{Var}(M_a|N_a, x) + (1-x)^2\mathrm{Var}_a(N_a)]}},$$

with $q(N_a)$ being the probability distribution of the spike number of the ath neuron in a synchronous event. We will explain the general meaning of $\mathrm{Var}(M_a|N_a, x)$ in **Section 4.1** below (**eq.21**); but here (i.e. in **Supplementary Figure 2**), we use Model Sync (see **Materials and Methods** in the main text) to generate our spike trains, in which case $\mathrm{Var}(M_a|N_a, x)$ can be understood as the variance of the number of chosen spikes if each of the N_a spikes are chosen independently with probability x. In this case, M_a follows binomial distribution, whose mean is $N_a x$

and variance is $N_a x(1-x)$. Therefore (see also **eq.17**),

$$\rho_{PD} = \frac{\int \mathrm{d}N_a q(N_a) N_a x(1-x) - x(1-x)\mathrm{Var}_a(N_a)}{\sqrt{[\int \mathrm{d}N_a q(N_a) N_a x(1-x) + x^2 \mathrm{Var}_a(N_a)] \cdot [\int \mathrm{d}N_a q(N_a) N_a x(1-x) + (1-x)^2 \mathrm{Var}_a(N_a)]}}$$

$$= \frac{x(1-x) - x(1-x)CV_{SpikeNum}^2}{\sqrt{[x(1-x) + x^2 CV_{SpikeNum}^2] \cdot [x(1-x) + (1-x)^2 CV_{SpikeNum}^2]}}$$

From this equation, we can see that $\rho_{PD} > 0$ if $CV_{SpikeNum} < 1$, and $\rho_{PD} < 0$ if $CV_{SpikeNum} > 1$. What's more, if we let $a = CV_{SpikeNum}^2$, then it is easy to show that

$$\frac{\partial \rho_{PD}}{\partial a} = -\frac{(1+a)(1-x)^2 x^2}{2[(1-x)x((1-x)x + a^2(1-x)x + a(1-2x+2x^2))]^{\frac{3}{2}}}$$

As $a > 0$, $0 < x < 1$, it is easy to show that $\frac{\partial \rho_{PD}}{\partial a} < 0$, which means that ρ_{PD} increases with the decreasing of $CV_{SpikeNum}$. This explains the decrease of ρ_{PD} with $CV_{SpikeNum}$ and the positive ρ_{PD} when $CV_{SpikeNum}$ is small under $\tau_{cross} > \tau_{delay}$.

4 The Interaction of Synchronous Firing and Auto-correlation Structure

As we mentioned in the main text, auto-correlation structure comes into spike patterns with synchronous firing in at least three ways:

1) The broadness of the distribution of spike number per neuron per synchronous event ($\mathrm{AT_{SpikeNum}}$).

2) The burstiness/regularity of the pieces of spike train within synchronous events ($\mathrm{AT_{WithinEvent}}$).

3) The burstiness/regularity of the occurrence of synchronous events ($\mathrm{AT_{events}}$).

We have already discussed the influence of $\mathrm{AT_{SpikeNum}}$ onto DiffV in the main text, in the following part of this section, we will consider $\mathrm{AT_{WithinEvent}}$ and $\mathrm{AT_{events}}$. Overall, we find that broader distribution of spike number per neuron per synchronous event (for $\mathrm{AT_{SpikeNum}}$), burstier spike trains within synchronous events (for $\mathrm{AT_{WithinEvent}}$), and burstier occurrence of synchronous events tend to increase DiffV (for $\mathrm{AT_{events}}$). These results can be concluded into a rule of thumb: the burstiness of spike trains tends to increase DiffV, while the regularity tends to decrease DiffV.

4.1 The Burstiness of the Piece of Spike Train within a Synchronous Event

We use the following model (Model Sync-Auto 1) to generate spike trains in which both $\mathrm{AT_{SpikeNum}}$ and $\mathrm{AT_{WithinEvent}}$ can be explicitly controlled.

Model Sync-Auto 1:

In this model, the occurrence of synchronous events and the spike number that a neuron is to fire during a synchronous event are determined in the same way as Model Sync in the **Materials and Methods** of the main text ($CV_{SpikeNum} = 1$ by default). If a neuron is to fire M ($M > 0$) spikes during a synchronous event of duration τ_{cross}, then the piece of spike train during this interval will be generated as follows:

We first define a Gamma process with rate p/τ_{cross} (here p has the same meaning as it in Model Sync in the main text, which is the mean spike number of a neuron during a synchronous event) and coefficient of variance $CV_{WithinEvent}$, and then generate $M+2$ spikes using this Gamma process. Suppose that the time of the ith spike of the Gamma process is t_i ($0 \leq i \leq M+1$ and $t_0 = 0$), and the synchronous event is within the interval $[t_{event}, t_{event} + \tau_{cross}]$. Then the jth (with $1 \leq j \leq M$) spike of the neuron in the synchronous event will be at time

$$t_{event} + \mathrm{Mod}((t_j - \frac{t_1}{2})\frac{\tau_{cross}}{(\frac{t_M + t_{M+1}}{2} - \frac{t_1}{2})} + x, \tau_{cross})$$

with x being a random number uniformly chosen from the interval $[0, \tau_{cross}]$, and is fixed for all these M spikes.

The idea of this operation is that given a spike train of $M+2$ spikes, we first cut the spike train at the middle time between the 1st and 2nd spikes, and also cut at the middle time between the last 1st and last 2nd spikes. Then we rescale the left spike train to length τ_{cross}, and translationally shift the spike train by a random interval, implementing periodic condition to deal with the spikes being moved out of the time boundary of $[0, \tau_{cross}]$ during the shift. By doing this, the probability density that a spike appears at any time during the interval $[0, \tau_{cross}]$ is the same for different trials.

In this model, the burstiness of the spike train within a synchronous event is controlled by $CV_{WithinEvent}$. If $CV_{WithinEvent}$ is small, then the neurons will fire regularly in a synchronous event; if $CV_{WithinEvent}$ is large, then the neurons will fire burstly in a synchronous event.

Our simulations suggest that the burstiness of the piece of spike train within a synchronous event does not significantly influence DiffV when $\tau_{cross} \leq \tau_{delay}$, but it increases DiffV when $\tau_{cross} > \tau_{delay}$ (**Supplementary Figure 3A**). We also find that d and c_{II} are the main reasons for the increase of DiffV with $CV_{WithinEvent}$, c_I does not has significant effect (**Supplementary Figure 3B-D**).

4.1.1 The influence of $CV_{WithinEvent}$ to d

To understand the change of d with $CV_{WithinEvent}$, note that under the assumption $\tau_{cross} \ll \tau_{STDP}$, **eq.13** still applies to the case $\tau_{cross} \leq \tau_{delay}$, and **eqs.14-16** still apply to the case $\tau_{cross} > \tau_{delay}$. When $\tau_{cross} \leq \tau_{delay}$, the efficacy variance mainly comes from the difference of spike numbers of different non-central neurons in a synchronous event, therefore if the distribution of spike numbers per neuron per synchronous event is kept unchanged, then the burstiness of the piece of spike train within a synchronous event can hardly contribute to the efficacy variance: this is the reason why $CV_{WithinEvent}$ hardly influences d when $\tau_{cross} \leq \tau_{delay}$ (**Supplementary Figure 3B**). When $\tau_{cross} > \tau_{delay}$, we can see from **eqs.14** and **15** that the burstiness of the piece of spike train within $\mathcal{S}_0$ (i.e. the synchronous event that the central spike t_i lies in) may influence the efficacy variance through changing $\mathrm{Var}_a(N_a, x)$. As mentioned before, this factor means that if M_a of the N_a spikes of the ath non-central neuron lie in an interval of duration $\Delta\tau$ (with $\Delta\tau/\tau_{cross} = x$) in a synchronous event, then $\mathrm{Var}_a(N_a, x) = \mathrm{Var}_a(M_a)$. Therefore,

$$\mathrm{Var}_a(N_a, x) = \frac{M_1^2 + M_2^2 + \cdots + M_N^2}{N} - (\frac{M_1 + M_2 + \cdots + M_N}{N})^2$$

$$= \int \mathrm{d}N_a q(N_a)[\mathrm{Var}(M_a|N_a, x) + N_a^2 x^2] - (\int \mathrm{d}N_a q(N_a) N_a)^2 \cdot x^2$$

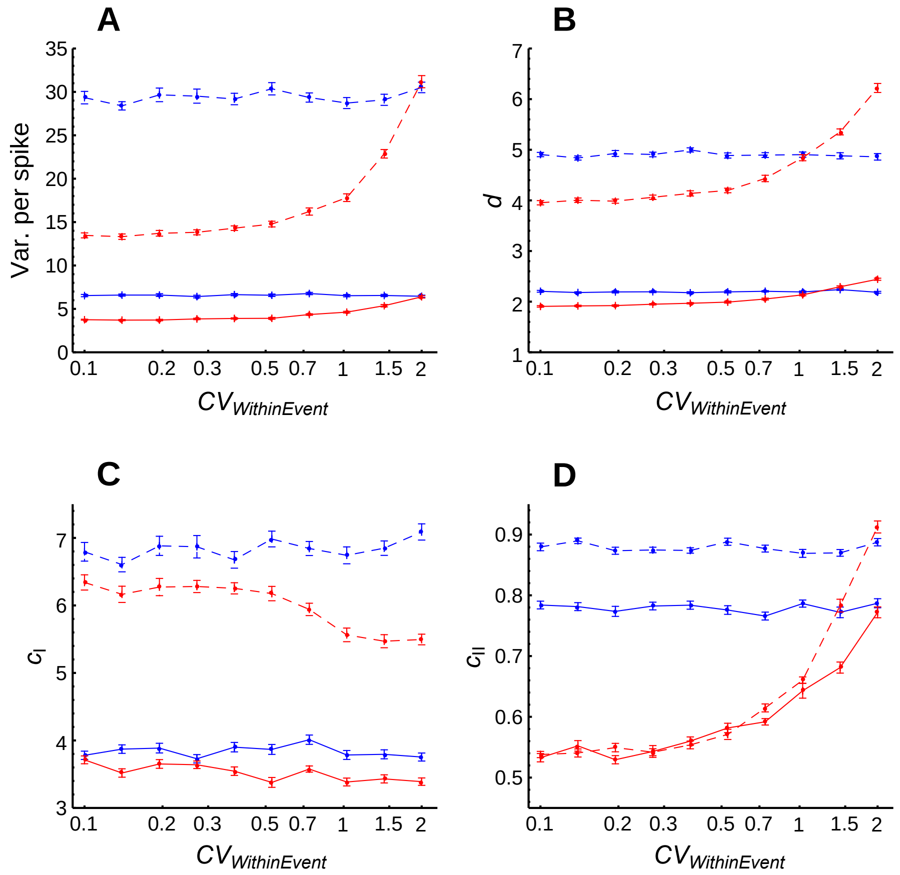

Figure 3: **How the burstiness of the piece of spike train within a synchronous event influences DiffV.** (**A**) Variance per spike as a function of $CV_{WithinEvent}$, when $\tau_{cross} = 0.5$ms (blue) or 2.5ms (red) and $p = 2$ (solid line) or 5 (dashed line). $\tau_{delay} = 1$ms. Spike trains are generated according to Model Sync-Auto 1 in **Section 4.1**. (**B-D**) The same as **A**, but for d, c_I and c_{II}. In **A-D**, the size of the converging motif, the parameters for STDP as well as the simulation time and trials are the same as in **Figure 4** in the main text. Error bars represent s.e.m.

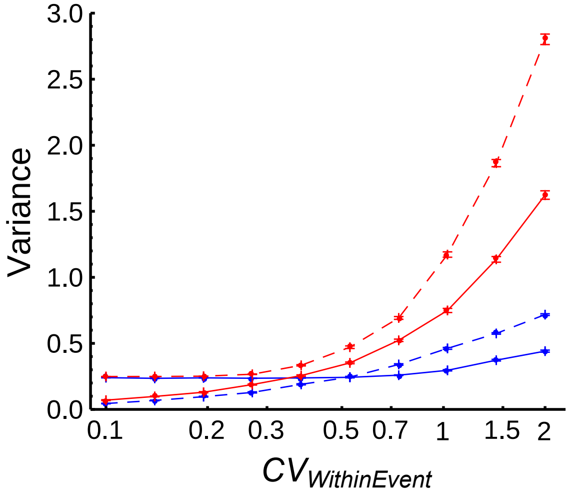

Figure 4: **Understanding the influence of $CV_{WithinEvent}$ onto d.** $\mathrm{Var}(M_a|N_a,x)$ (see **eq.21**) as a function of $CV_{WithinEvent}$ when $x=0.2$ (solid line) or 0.5 (dashed line) and $p=2$ (blue) or 5 (red).

$$= \int \mathrm{d}N_a q(N_a)\mathrm{Var}(M_a|N_a,x) + x^2\mathrm{Var}_a(N_a), \tag{21}$$

with $q(N_a)$ being the probability distribution of N_a, $\mathrm{Var}(M_a|N_a,x)$ being the variance of M_a if the spike number of the ath non-central neuron is N_a and $\mathrm{E}_T(M_a|N_a) = N_a x$. As the burstiness of the piece of spike train within a synchronous event (which is, here, quantified by $CV_{WithinEvent}$) increases $\mathrm{Var}(M_a|N_a,x)$ (**Supplementary Figure** 4), it also increases $\mathrm{Var}_a(N_a,x)$ through the equation above, and thereby increases $\mathrm{E}_T(\mathrm{Var}_a(\sum_{j\in\mathcal{S}_0}\Delta w_{a,d}(t_i,t_j)))$ and $\mathrm{E}_T(\mathrm{Var}_a(\sum_{j\in\mathcal{S}_0}\Delta w_{a,p}(t_i,t_j)))$ through **eqs.14** and **15**. This explains the increase of d with $CV_{WithinEvent}$ under $\tau_{cross} > \tau_{delay}$.

4.1.2 The influence of $CV_{WithinEvent}$ to c_{II}

From **eqs.15** and **16** in the main text,

$$c_{II} = 1 + \rho_{PD} f_{PD}$$

with

$$f_{PD} = \frac{2\sqrt{\mathrm{Var}_a(\sum_i\sum_j \Delta w_{a,p}(t_i,t_j)) \cdot \mathrm{Var}_a(\sum_i\sum_j \Delta w_{a,d}(t_i,t_j))}}{\sum_k \mathrm{Var}_a(\sum_i\sum_j \Delta w_{a,k}(t_i,t_j))},$$

with ρ_{PD} being the correlation coefficient between the total potentiation and depression values imposed on the same synapse, and f_{PD} is the coupling factor. To understand the change of c_{II} with $CV_{WithinEvent}$ under $\tau_{cross} > \tau_{delay}$, we plot ρ_{PD} and f_{PD} with $CV_{WithinEvent}$, and find that ρ_{PD} is the main reason for the increase of c_{II} with $CV_{WithinEvent}$, the contribution of the coupling factor is not significant (**Supplementary Figure** 5**AB**).

ρ_{PD} tends to be negative, which is because of the heterogeneity of spike numbers of the non-central neurons. If the ath non-central neuron fires more spikes, then both the potentiation and depression processes on the ath synapse will get stronger. This is the reason why $\mathrm{Corr}_a(\Delta w_{a,p}, N_a) > 0$ (with N_a being the spike number of the ath non-central neuron) and $\mathrm{Corr}_a(\Delta w_{a,d}, N_a) < 0$ (**Supplementary Figure 5C**), which makes $\rho_{PD} = \mathrm{Corr}_a(\Delta w_{a,p}, \Delta w_{a,d}) < 0$.

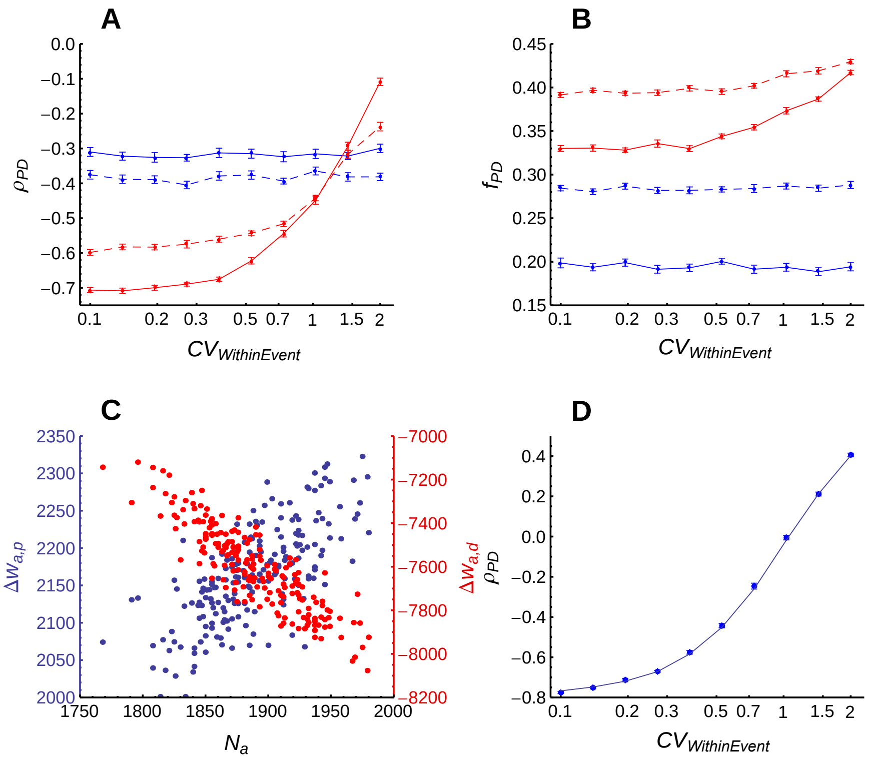

Figure 5: **Understanding the influence $CV_{WithinEvent}$ onto c_{II}.** (**A**) ρ_{PD} as a function of $CV_{WithinEvent}$, when $\tau_{cross} = 0.5$ms (blue) or 2.5ms (red) and $p = 2$ (solid line) or 5 (dashed line). $\tau_{delay} = 1$ms. Spike trains are generated according to Model Sync-Auto 1 in **Section 4.1**. (**B**) The same as **A**, but for f_{PD}. Note that the percentage of the change of f_{PD} is significantly smaller than that of ρ_{PD}. (**C**) The spike number of a non-central neuron (horizontal coordinate) is positively correlated with the total potentiation (blue) value and negatively correlated with the total depression (red) value. (**D**) The potentiation and depression correlation coefficient of the simple model introduced in **Section 4.1.2**. The dots with error bars are numeric results, while the solid line is calculated from **eq.22**. In **A-D**, the size of the converging motif, the parameters for STDP as well as the simulation time and trials are the same as in **Figure 4** in the main text. Error bars represent s.e.m.

We find that $CV_{WithinEvents}$ hardly influences ρ_{PD} when $\tau_{cross} \leq \tau_{delay}$, but increases ρ_{PD} when $\tau_{cross} > \tau_{delay}$ (**Supplementary Figure 5A**). Now we try to understand this phenomenon in the following discussion.

Suppose an central spike at time t_i and the synchronous event $\mathcal{S}_0$ that the spike belongs to, let us consider a synchronous event $\mathcal{S}_-$ that occurs before $\mathcal{S}_0$, and a synchronous event $\mathcal{S}_+$ that occurs after $\mathcal{S}_0$. Then under the case that the inter-event interval p/r_0 is large enough (which is realized when p is large) and the occurrence of synchronous events is not too bursty, it is likely that

$$t_i - \tau_{delay} > t_j, \text{ for all } t_j \in \mathcal{S}_-,$$

$$t_i - \tau_{delay} < t_j, \text{ for all } t_j \in \mathcal{S}_+.$$

If $\tau_{cross} \ll \tau_{STDP}$, then the synaptic updating caused by pairing t_i and non-central spikes in $\mathcal{S}_-$ and $\mathcal{S}_+$ can be

approximated by

$$\Delta w_a(t_i, t_{j\in\mathcal{S}_-}) \approx A_p \exp(-\frac{(t_i - \tau_{delay}) - t_{\mathcal{S}_-}}{\tau_{STDP}}),$$

and

$$\Delta w_a(t_i, t_{j\in\mathcal{S}_+}) \approx -A_d \exp(-\frac{t_{\mathcal{S}_+} - (t_i - \tau_{delay})}{\tau_{STDP}}),$$

with $t_{\mathcal{S}_-}$ and $t_{\mathcal{S}_+}$ respectively being the middle time of $\mathcal{S}_-$ and $\mathcal{S}_+$. In the equations above, we have used similar approximations as in **eq.8**. We can see that the spike patterns within $\mathcal{S}_-$ and $\mathcal{S}_+$ hardly have influence onto the synaptic weights.

Similar arguments also apply to $\mathcal{S}_0$ when $\tau_{cross} \leq \tau_{delay}$, because in this case, all the spikes of the non-central neurons within $\mathcal{S}_0$ depress the synapses with their interactions with t_i, and

$$\Delta w_a(t_i, t_{j\in\mathcal{S}_0}) \approx -A_d \exp(-\frac{\tau_{delay}}{\tau_{STDP}}),$$

so that the spike pattern within $\mathcal{S}_0$ also hardly influences synaptic weights. This explains why $CV_{WithinEvent}$ has little influence onto ρ_{PD} when $\tau_{cross} \leq \tau_{delay}$ (**Supplementary Figure 5A**).

However, the situation is different when $\tau_{cross} > \tau_{delay}$, because the equation above is no longer valid in this case, and $\Delta w_a(t_i, t_{j\in\mathcal{S}_0})$ is larger than zero if $t_j + \tau_{delay} < t_i$. From **Supplementary Figure 5A**, ρ_{PD} increases with $CV_{WithinEvent}$ when $\tau_{cross} > \tau_{delay}$, but not when $\tau_{cross} \leq \tau_{delay}$, which suggests that the possibility that $\Delta w_a(t_i, t_{j\in\mathcal{S}_0}) > 0$ under $\tau_{cross} > \tau_{delay}$ is a key point to understand the increase of ρ_{PD} with $CV_{WithinEvent}$.

Intuitively, during a synchronous event $(0, \tau_{cross})$ (with $\tau_{cross} > \tau_{delay}$), the non-central spikes that are emitted during $(0, t_i - \tau_{delay})$ will potentiate the synapses, and the non-central spikes that are emitted during $(t_i - \tau_{delay}, \tau_{cross})$ will depress the synapses. If $CV_{WithinEvent}$ is large, then the piece of spike train within a synchronous event will be bursty, so that the following situation is likely to happen: if the ath non-central neuron fires all its spikes during $(0, t_i - \tau_{delay})$, then all its spikes potentiate the ath synapse, so that $\Delta w_{a,p} > 0$ while $\Delta w_{a,d} = 0$; however, if the ath non-central neuron fires all its spikes during $(t_i - \tau_{delay}, \tau_{cross})$, then $\Delta w_{a,p} = 0$ and $\Delta w_{a,d}$ will be very negative. This seems to be a possible mechanism that positively correlate $\Delta w_{a,p}$ and $\Delta w_{a,d}$ when $CV_{WithinEvent}$ is large (**Supplementary Figure 5A**). Mathematically, it is complicated to analytically calculate ρ_{PD}, but we can implement this idea using the following simple model, which focuses on a single synchronous event, thereby helps to understand the increase of ρ_{PD} with $CV_{WithinEvent}$ under $\tau_{cross} > \tau_{delay}$.

Let's suppose $\mathcal{S}_0$ happens during $(0, \tau_{cross})$ with $\tau_{cross} > \tau_{delay}$, and the spike of the central neuron is at $t_i - \tau_{delay} = x\tau_{cross}$ with $0 < x < 1$, so that x portion of the spikes of the non-central neuron potentiate the synapses, while $1 - x$ portion depress the synapses. If $\tau_{cross} \ll \tau_{STDP}$, then we can suppose that all the spikes within $(0, x\tau_{cross})$ will potentiate the synapses by $y_p \approx A_p \exp(-\frac{x\tau_{cross}}{2\tau_{STDP}})$, and all the spikes within $(x\tau_{cross}, \tau_{cross})$ will depress the synapses by $-y_d \approx -A_d \exp(-\frac{\tau_{cross}(1-x)}{2\tau_{STDP}})$. With the same as **eq.21**, we denote N_a as the spike number of the ath non-central neuron during $\mathcal{S}_0$, and denote M_a as the spike number of the ath non-central neuron within $(0, x\tau_{cross})$. Then, the variance of the potentiation value caused by the spikes within $(0, x\tau_{cross})$ is

$$VarP = \mathrm{Var}_a(M_a x_p) = x_p^2 \mathrm{Var}_a(M_a)$$

$$= x_p^2[\int \mathrm{d}N_a q(N_a)\mathrm{Var}(M_a|N_a, x) + x^2\mathrm{Var}_a(N_a)],$$

where we use **eq.21** in the last step. Similarly, the variance of the depression value caused by the spikes within

$(x\tau_{cross}, \tau_{cross})$ is

$$VarD = \text{Var}_a((N_a - M_a)x_d) = x_d^2 \text{Var}_a(N_a - M_a)$$
$$= x_d^2 [\int \mathrm{d}N_a q(N_a) \text{Var}(M_a|N_a, x) + (1-x)^2 \text{Var}_a(N_a)].$$

And the variance of the total STDP updating value caused by the spikes within $\mathcal{S}_0$ is

$$VarTot = \text{Var}_a(M_a x_p - (N_a - M_a)x_d)$$
$$= x_d^2 \text{Var}_a(N_a - \frac{x_p + x_d}{x_d} M_a)$$
$$= x_d^2 [\int \mathrm{d}N_a q(N_a) \text{Var}(\frac{x_p + x_d}{x_d} M_a | N_a, x) + (1 - \frac{x_p + x_d}{x_d} x)^2 \text{Var}_a(N_a)]$$
$$= (x_p + x_d)^2 \int \mathrm{d}N_a q(N_a) \text{Var}(M_a|N_a) + (x_d(1-x) - x_p x)^2 \text{Var}_a(N_a)$$

Therefore,

$$\rho_{PD} = \frac{VarTot - VarP - VarD}{2\sqrt{VarP \cdot VarD}}$$
$$= \frac{\int \mathrm{d}N_a q(N_a) \text{Var}(M_a|N_a, x) - x(1-x)\text{Var}_a(N_a)}{\sqrt{[\int \mathrm{d}N_a q(N_a) \text{Var}(M_a|N_a, x) + x^2 \text{Var}_a(N_a)] \cdot [\int \mathrm{d}N_a q(N_a) \text{Var}(M_a|N_a, x) + (1-x)^2 \text{Var}_a(N_a)]}}. \quad (22)$$

If we let

$$a = \int \mathrm{d}N_a q(N_a) \text{Var}(M_a|N_a, x)$$
$$b = \text{Var}_a(N_a),$$

then it is easy to show that

$$\frac{\partial \rho_{PD}}{\partial a} = \frac{b[a + b(1-x)x]}{2[(a + b(1-x)^2)(a + bx^2)]^{\frac{3}{2}}}.$$

As $a > 0$, $b > 0$, $0 < x < 1$, $\frac{\partial \rho_{PD}}{\partial a} > 0$. Thus, ρ_{PD} is an increasing function of $\text{Var}(M_a|N_a, x)$. Because $\text{Var}(M_a|N_a, x)$ increases with $CV_{WithinEvent}$ (**Supplementary Figure 4**), ρ_{PD} increases with $CV_{WithinEvent}$. We compare numeric results with the results calculated from **eq.22** in **Supplementary Figure 5D**.

4.1.3 The influence of $CV_{WithinEvent}$ to c_I

From **Supplementary Figure 3C**, we know that c_I does not significantly influence DiffV when $CV_{WithinEvent}$ changes, which means that it is not an important factor to understand the change of DiffV with $CV_{WithinEvent}$. Here, we discuss the change of c_I with $CV_{WithinEvent}$ only for completeness. Readers may skip this subsection when reading for the first time.

Suppose a spike of the central neuron $t_i \in \mathcal{S}_1$ (with $\mathcal{S}_1$ being a synchronous event), and a spike of the ath non-central neuron $t_{a,j} \in \mathcal{S}_2$ (with $\mathcal{S}_2$ also being a synchronous event). If the inter-event interval p/r_0 is large and the occurrence of synchronous events is not too bursty, then under $\tau_{cross} \leq \tau_{delay}$, it is likely that:

1) If $\mathcal{S}_2$ occurs before $\mathcal{S}_1$, then

$$t_i - \tau_{delay} > t_{a,j}, \text{ for all } t_{a,j} \in \mathcal{S}_2;$$

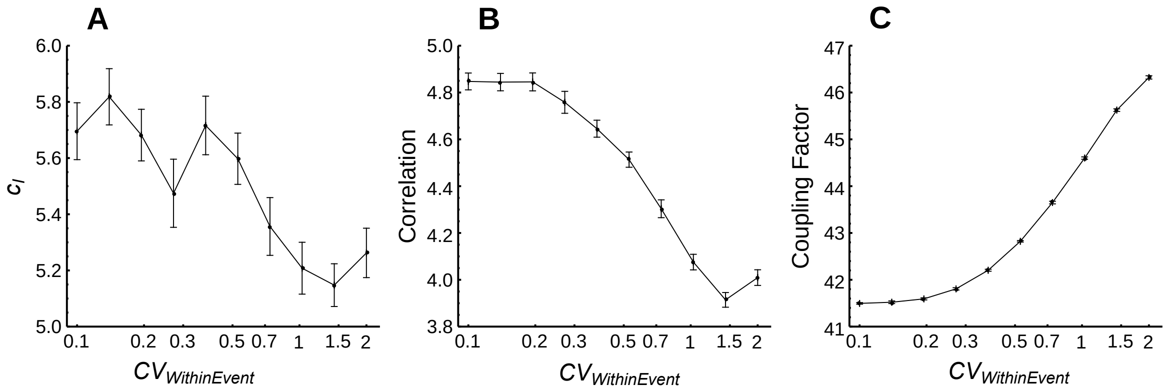

Figure 6: **Understanding the influence of $CV_{WithinEvent}$ onto c_I.** (**A**) c_I as a function of $CV_{WithinEvent}$ when $\tau_{cross} = 2.5$ms, $p = 5$. Here, we set $\tau_{delay} = 0$ms to understand the effect of $\tau_{cross} > \tau_{delay}$. Spike trains are generated according to Model Sync-Auto 1 in **Section 4.1**. (**B**) The same as **A**, but for $\sum_{l,k} \rho_{l,k}$ (see **eq.30** in the main text). We found that $\rho_{l,k}$ safely decays to zero when $l \geq 50$ in our parameter range, therefore we cut off l at $l = 50$ when calculating the summation. (**C**) The same as **B**, but for $\sum_{l,k} f_{l,k}$ (see **eq.31** in the main text). In **A-C**, the size of the converging motif, the parameters for STDP, as well as the simulation time and trials are the same as in **Figure 4** in the main text. Error bars represent s.e.m.

2) If $\mathcal{S}_2$ occurs after $\mathcal{S}_1$ or $\mathcal{S}_2 = \mathcal{S}_1$, then

$$t_i - \tau_{delay} < t_{a,j}, \text{ for all } t_{a,j} \in \mathcal{S}_2.$$

Under $\tau_{cross} \leq \tau_{delay}$, the first condition above implies that the synaptic updating caused by pairing t_i and $t_{a,j}$ can be approximated by

$$\Delta w_a(t_i, t_{a,j}) \approx A_p \exp(-\frac{t_{\mathcal{S}_1} - (t_{\mathcal{S}_2} + \tau_{delay})}{\tau_{STDP}}),$$

(with $t_{\mathcal{S}_1}$ and $t_{\mathcal{S}_2}$ being the middle time of $\mathcal{S}_1$ and $\mathcal{S}_2$); and the second condition above implies that

$$\Delta w_a(t_i, t_{a,j}) \approx -A_d \exp(-\frac{(t_{\mathcal{S}_2} + \tau_{delay}) - t_{\mathcal{S}_1}}{\tau_{STDP}}).$$

These two equations suggest that when $\tau_{cross} \leq \tau_{delay}$, the spike pattern within the piece of spike train within a synchronous event does not have significant effect on the synaptic changes $\Delta w_a(t_i, t_{a,j})$, which is the reason why c_I hardly changes with $CV_{WithinEvent}$ when $\tau_{cross} \leq \tau_{delay}$ (**Supplementary Figure 3C**).

When $\tau_{cross} > \tau_{delay}$, some non-central spikes in $\mathcal{S}_1$ may potentiate the synapses after pairing with t_i, and some others may depress the synapses. In this case, it is difficult to understand the change of c_I with $CV_{WithinEvent}$, and we resorted to simulations. Our simulations suggest that c_I may slightly decrease with $CV_{WithinEvent}$ in this case, and the decrease of correlations instead of coupling factors is the reason for this phenomenon (**Supplementary Figure** 6). But overall, the influence of $CV_{WithinEvent}$ onto c_I is not strong.

4.2 The Burstiness of the Occurrence of Synchronous Events

To investigate the influence of the burstiness of the occurrence of synchronous events onto DiffV, we use the following model to generate spike trains:

Model Sync-Auto 2:

In this model, the spike train of a neuron within a synchronous event is determined in the same way as Model Sync in the main text (we set $CV_{SpikeNum} = 1$ by default). The occurrence of synchronous event is a Gamma process with rate p/τ_{cross} and coefficient of variance CV_{events}. If CV_{events} is small, then synchronous events will occur regularly; if CV_{events} is large, then synchronous events will occur burstly.

Our simulations suggest that the burstiness of the occurrence of synchronous events tends to increase DiffV, especially when $CV_{events} > 1$ (**Supplementary Figure 7A**). It does this through d and c_I (**Supplementary Figure 7BC**), while c_{II} contributes negatively (**Supplementary Figure 7D**).

4.2.1 The influence of CV_{events} to d

CV_{events} influences d mainly through the synchronous events other than $\mathcal{S}_0$, i.e. $\mathrm{E}_T(\mathrm{Var}_a(\sum_{j\notin\mathcal{S}_0}\Delta w_{a,d}(t_i,t_j)))$ and $\mathrm{E}_T(\mathrm{Var}_a(\sum_{j\notin\mathcal{S}_0}\Delta w_{a,p}(t_i,t_j)))$ (see **eqs.10** and **11**). To understand the underlying mechanism of this influence, we let $\tau_{delay} = 0$. In this case, **eq.16** becomes

$$\sum_{k=p,d}\mathrm{E}_T(\mathrm{Var}_a(\sum_{j\notin\mathcal{S}_0}\Delta w_{a,k}(t_i,t_j))) \approx \mathrm{Var}_a(N_a)(A_p^2 + A_d^2)\cdot\mathrm{E}_T[\sum_{l=1}^{\infty}\exp(-\frac{2\tau_l}{\tau_{STDP}})] \tag{23}$$

with $\{\tau_l\}$ $(l = 0, 1, \cdots)$ being a Gamma process with rate r_0/p and coefficient of variance CV_{events}, starting from $\tau_0 = 0$. From **Supplementary Figure 8**, we can see that $\mathrm{E}_T[\sum_{l=1}^{\infty}\exp(-\frac{2\tau_l}{\tau_{STDP}})]$ monotonically increases with CV_{events}, thereby increasing d if $\mathrm{Var}_a(N_a) \neq 0$. Intuitively, the increasing of $\mathrm{E}_T[\sum_{m=1}^{\infty}\exp(-\frac{2\tau_l}{\tau_{STDP}})]$ means that that more synchronous events can be gathered closer to $\mathcal{S}_0$ with the increasing of CV_{events}.

4.2.2 The influence of CV_{events} to c_I

Following similar procedure as in **Figure 7** in the main text, we find that the increase of c_I with CV_{events} is mainly due to the increase of correlation coefficients $\rho_{m,n;k}$ (see **eq.17** in the main text) (**Supplementary Figure 9A**), while the coupling factors $f_{m,n;k}$ (see **eq.17** in the main text) don't have significant contributions (**Supplementary Figure 9B**). To understand the increase of $\sum_{m<n}\rho_{m,n;k}$ with CV_{events}, let's for simplicity only consider the case $\tau_{cross} < \tau_{delay} \ll \frac{p}{r_0}$ (with p/r_0 being the inter-event interval). This calculation will help us gain insight on the mechanisms how CV_{events} increases $\rho_{m,n;k}$.

In this case, the depression of the ath synapse caused by the mth spike (which belongs to the synchronous event $\mathcal{S}_0$) of the central neuron can be approximated by

$$\Delta w_{a,d}(t_m \in \mathcal{S}_0) \approx -A_d\exp(-\frac{\tau_{delay}}{\tau_{STDP}})[N_{a,0} + \sum_{l=1}^{\infty}N_{a,l}\exp(-\frac{\tau_l}{\tau_{STDP}})] \tag{24}$$

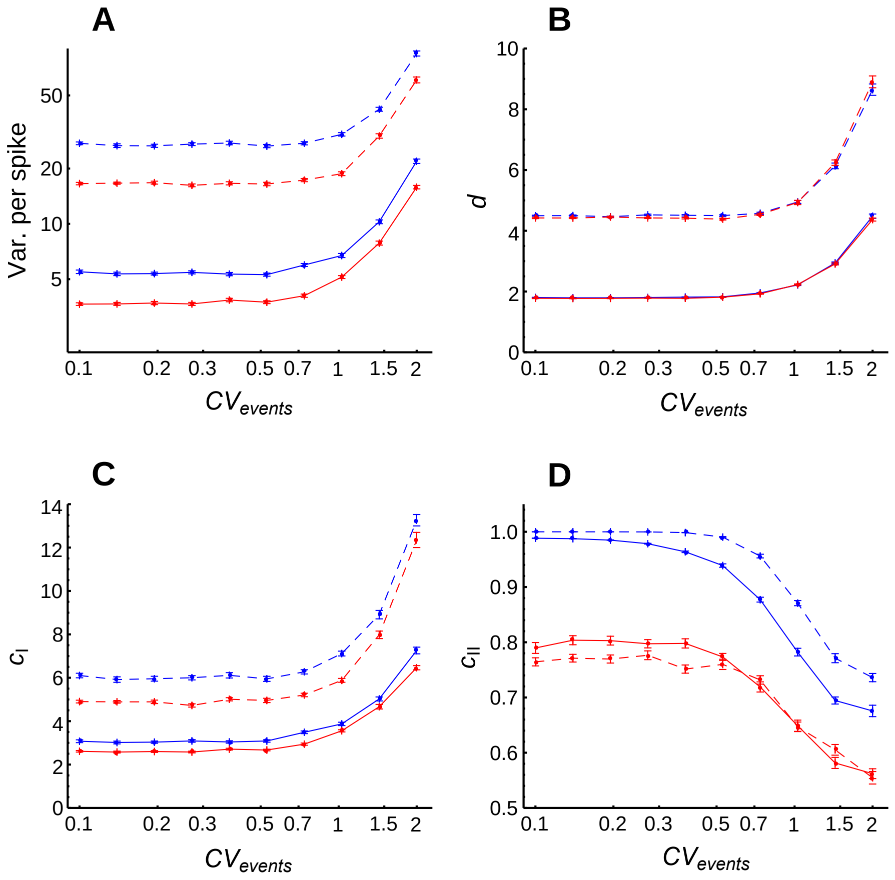

Figure 7: **How the burstiness of the occurrence of synchronous events influences DiffV.** (**A**) Variance per spike as a function of CV_{events}, when $\tau_{cross} = 0.5$ms (blue) or 2.5ms (red) and $p = 2$ (solid line) or 5 (dashed line). $\tau_{delay} = 1$ms. Spike trains are generated according to Model Sync-Auto 2 in **Section 4.2**. (**B-D**) The same as **A**, but for d, c_I and c_{II}. In **A-D**, the size of the converging motif, the parameters for STDP, as well as the simulation time and trials are the same as in **Figure 4** in the main text. Error bars represent s.e.m.

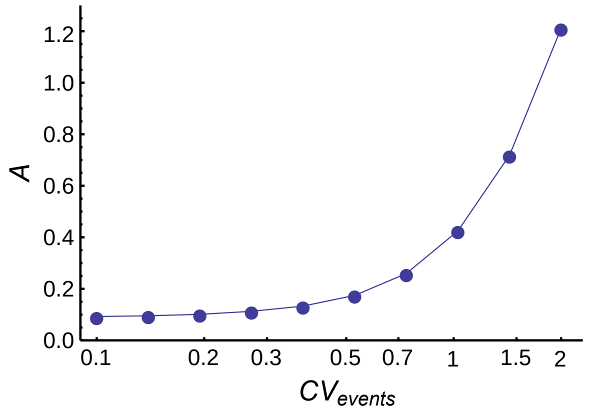

Figure 8: **Understanding the influence of CV_{events} onto d.** The change of $\mathcal{A} = \mathrm{E}_T[\sum_{l=1}^{\infty} \exp(-\frac{2\tau_l}{\tau_{STDP}})]$ with CV_{events} (see **eq.23**). The results were from averaging over 10000 trials of Gamma processes with rate r_0/p (here we set $r_0 = 20$Hz, $p = 5$).

with $\{\tau_l\}$ ($l = 0, 1, \cdots$) being a Gamma process with rate r_0/p and coefficient of variance CV_{events}, starting from $\tau_0 = 0$, and $N_{a,l}$ being the spike number of the ath non-central neuron in the lth synchronous event after $\mathcal{S}_0$. In the equation above, we omit the contribution to $\Delta w_{a,d}(t_m)$ by the synchronous events that happens during the interval $[t_m - \tau_{delay}, t_m]$. This approximation is acceptable if τ_{delay} is far smaller than the inter-event interval p/r_0 (which is particularly correct when p is large) and when the occurrence of synchronous events is not too bursty. From **eq.24**, we know that if t_m and t_n are two spikes of the central neuron that belong to two immediately adjacent synchronous events $\mathcal{S}_0$ and $\mathcal{S}_1$, then

$$\Delta w_{a,d}(t_m \in \mathcal{S}_0) \approx -A_d \exp(-\frac{\tau_{delay}}{\tau_{STDP}}) N_{a,0} + \exp(-\frac{\tau_1}{\tau_{STDP}}) \Delta w_{a,d}(t_n \in \mathcal{S}_1) \tag{25}$$

Then

$$\begin{aligned} \rho_{m,n;d} &= \mathrm{Corr}_a(\Delta w_{a,d}(t_m), \Delta w_{a,d}(t_n)) \\ &= \mathrm{Corr}_a(X_a + bY_a, Y_a) \end{aligned} \tag{26}$$

with

$$X_a = -A_d \exp(-\frac{\tau_{delay}}{\tau_{STDP}}) N_{a,0} \tag{27}$$

,

$$Y_a = \Delta w_{a,d}(t_n) \tag{28}$$

and

$$b = \exp(-\frac{\tau_1}{\tau_{STDP}}) \tag{29}$$

From **eq.26**, we can see that $\Delta w_{a,d}(t_m)$ and $\Delta w_{a,d}(t_n)$ are correlated together by sharing the term Y_a, and their correlation can be increased if the variance of the correlated term bY_a is increased, which can be realized by increasing either $|b|$ or $\mathrm{Var}_a(Y_a)$. We will see how CV_{events} increases their correlation through b^2 and $\mathrm{Var}_a(Y_a)$ in the following discussion.

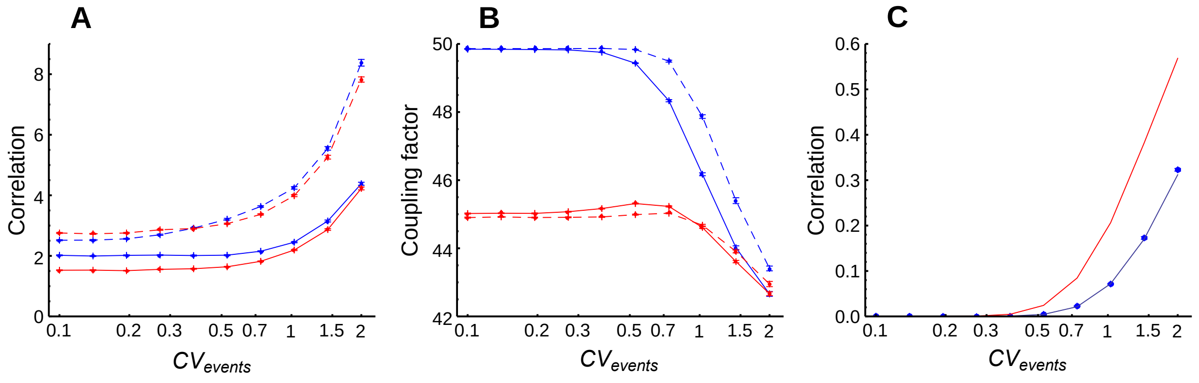

Figure 9: **Understanding the influence of CV_{events} onto c_I.** (**A**) The change of $\sum_{l,k}\rho_{l,k}$ (see **eq.30** in the main text) with CV_{events} when $\tau_{cross}=0.5$ms (blue) or 2ms (red) and $p=2$ (solid line) or 5 (dashed line). Spike trains are generated according to Model Sync-Auto 2 in **Section 4.2**. We found that $\rho_{l,k}$ safely decays to zero when $l\geq 50$ in our parameter range, therefore we cut off l at $l=50$ when calculating the summation. (**B**) The same as **A**, but for $\sum_{l,k}f_{l,k}$ (see **eq.31** in the main text). Note that the percentage of the change of $\sum_{l,k}f_{l,k}$ is significantly smaller than that of $\sum_{l,k}\rho_{l,k}$. (**C**) $\mathrm{E}_T(\rho_{m,n;d})$ as a function of CV_{events}, with m and n represent two central spikes in immediately adjacent synchronous events. The dots with error bars give simulation results; the blue line represents results calculated from **eq.30**, using the distributions of b^2 and $\mathrm{Var}_a(Y_a)$; the red line represents results calculated by replacing b^2 and $\mathrm{Var}_a(Y_a)$ in **eq.30** with $\mathrm{E}_T(b^2)$ and $\mathrm{E}_T(\mathrm{Var}_a(Y_a))$. In **A-B**, the size of the converging motif, the parameters for STDP, as well as the simulation time and trials are the same as in **Figure 4** in the main text. In **C**, the statistics of b^2 was from 10000 trials of the Gamma process, and the statistics of $\mathrm{Var}_a(Y_a)$ was from simulations of the same conditions as in **A** and **B**. Error bars represent s.e.m.

As $N_{a,0}$ is indepedent of $N_{a,l}$ in **eq.24**, $\mathrm{Corr}_a(X_a,Y_a)=0$. Therefore **eq.26** becomes

$$\rho_{m,n;d}=\mathrm{Corr}_a(X_a+bY_a,Y_a)=\sqrt{\frac{b^2\mathrm{Var}_a(Y_a)}{\mathrm{Var}_a(X_a)+b^2\mathrm{Var}_a(Y_a)}}. \tag{30}$$

We have to use the distribution of b^2 and $\mathrm{Var}_a(Y_a)$ to calculate $\mathrm{E}_T(\rho_{m,n;d})$. Even if it is possible to do so, such calculation has little help for us to intuitively understand the physical mechanisms why $\mathrm{E}_T(\rho_{m,n;d})$ changes with CV_{events}. For a good understanding of this mechanism, we will estimate how $\mathrm{E}_T(b^2)$ and $\mathrm{E}_T(\mathrm{Var}_a(Y_a))$ change with CV_{events} (note that $\mathrm{E}_T(\mathrm{Var}_a(X_a))$ does not change with CV_{events}), and then compare $\mathrm{E}_T(\rho_{m,n;d})$ with $\sqrt{\frac{\mathrm{E}_T(b^2)\mathrm{E}_T(\mathrm{Var}_a(Y_a))}{\mathrm{Var}_a(X_a)+\mathrm{E}_T(b^2)\mathrm{E}_T(\mathrm{Var}_a(Y_a))}}$ in the following discussion.

When the occurrence of synchronous events is a Gamma process of rate r_0/p and coefficient of variance CV_{events}, the distribution of τ_1 is

$$q(\tau_1)=\frac{\beta^\alpha}{\Gamma(\alpha)}\tau_1^{\alpha-1}\exp(-\beta\tau_1)$$

with $\alpha=\frac{1}{CV_{events}^2}$ and $\beta=\frac{r_0}{CV_{events}^2 p}$. From **eq.29**,

$$\mathrm{E}_T(b^2)\approx\int_0^\infty\exp(-\frac{2\tau_1}{\tau_{STDP}})q(\tau_1)\mathrm{d}\tau_1=(\frac{\tau_{STDP}r_0}{\tau_{STDP}r_0+2pCV_{events}^2})^{\frac{1}{CV_{events}^2}}$$

To estimate how $\mathrm{E}_T(b^2)$ changes with CV_{events}, note that

$$\log[\frac{1}{\mathrm{E}_T(b^2)}] = \frac{1}{CV_{events}^2}\log[1+\frac{2pCV_{events}^2}{\tau_{STDP}r_0}],$$

and it is easy to show that $\log(1+ax)/x$ is a decreasing function of x if $a > 0$. Therefore, $\mathrm{E}_T(b^2)$ is an increasing function of CV_{events}^2.

What's more,

$$\mathrm{E}_T(\mathrm{Var}_a(Y_a)) = \mathrm{Var}_a(N_a)A_d^2\exp(-\frac{2\tau_{delay}}{\tau_{STDP}})\{1+\mathrm{E}_T[\sum_{l=1}^{\infty}\exp(-\frac{2\tau_l}{\tau_{STDP}})]\}$$

Therefore, $\mathrm{E}_T(\mathrm{Var}_a(Y_a))$ will increase with CV_{events} because of the increase of $\mathrm{E}_T[\sum_{l=1}^{\infty}\exp(-\frac{2\tau_l}{\tau_{STDP}})]$ (**Supplementary Figure 8**).

The discussions above shows that both $\mathrm{E}_T(b^2)$ and $\mathrm{E}_T(\mathrm{Var}_a(Y_a))$ increase with CV_{events}. As $\rho_{m,n;k}$ is an increasing function of both b^2 and $\mathrm{Var}_a(Y_a)$ (**eq.30**), our calculations above give an understanding on why $\mathrm{E}_T(\rho_{m,n;d})$ increases with CV_{events}. From **eq.26**, we can see that $\Delta w_{a,d}(t_m)$ and $\Delta w_{a,d}(t_n)$ are correlated together by sharing the term Y_a, and their correlation can be increased if the variance of the correlated term bY_a is increased, which can be realized by increasing either $|b|$ or $\mathrm{Var}_a(Y_a)$. From the discussions above, we see that CV_{events} increases the correlation coefficient through both of the two mechanisms.

In **Supplementary Figure 6C**, we compare $\mathrm{E}_T(\rho_{m,n;d})$ with $\sqrt{\frac{\mathrm{E}_T(b^2)\mathrm{E}_T(\mathrm{Var}_a(Y_a))}{\mathrm{Var}_a(X_a)+\mathrm{E}_T(b^2)\mathrm{E}_T(\mathrm{Var}_a(Y_a))}}$, we see that although they do not coincide, they have the same tendency to increase with CV_{events}. What's more, $\sqrt{\frac{\mathrm{E}_T(b^2)\mathrm{E}_T(\mathrm{Var}_a(Y_a))}{\mathrm{Var}_a(X_a)+\mathrm{E}_T(b^2)\mathrm{E}_T(\mathrm{Var}_a(Y_a))}} >$ $\mathrm{E}_T(\rho_{m,n;d})$, which suggests that the broad distribution of b^2 and $\mathrm{Var}_a(Y_a)$ tends to decrease $\mathrm{E}_T(\rho_{m,n;d})$.

4.2.3 The influence of CV_{events} to c_{II}

From **Supplementary Figure 7D**, we know that c_{II} negatively contributes to the increase of DiffV with CV_{events} especially when $CV_{events} > 0.7$, which means that c_{II} is not an important factor to understand the change of DiffV with CV_{events}. Here, we discuss the influence of CV_{events} onto c_{II} only for completeness, readers may skip this subsection when reading for the first time. When $CV_{events} > 0.7$, ρ_{PD} is negative and decreasing, while the coupling factor f_{PD} is positive and increasing (**Supplementary Figure 10AB**). This means that both ρ_{PD} and f_{PD} contribute to the decreasing of c_{II} with CV_{events}. We will try to understand the mechanisms underlying their contributions in this subsection.

The potentiation and depression on the ath synapse can be written as

$$\Delta w_{a,p} = \Delta w_{a,p}(\text{same}) + \Delta w_{a,p}(\text{diff}) \tag{31}$$

$$\Delta w_{a,d} = \Delta w_{a,d}(\text{same}) + \Delta w_{a,d}(\text{diff}) \tag{32}$$

with $\Delta w_{a,p}(\text{same})$ ($\Delta w_{a,d}(\text{same})$) being the potentiation (depression) caused by pairing central and non-central spikes that belong to the same synchronous event, and $\Delta w_{a,p}(\text{diff})$ ($\Delta w_{a,d}(\text{diff})$) being the potentiation (depression) caused by pairing central and non-central spikes that belong to different synchronous events. $\Delta w_{a,p}(\text{same})$ and $\Delta w_{a,d}(\text{same})$ are determined by the statistical features of a single synchronous event, while CV_{events} mainly changes

$\Delta w_{a,p}$(diff) and $\Delta w_{a,d}$(diff). To understand the underlying mechanism why ρ_{PD} decreases with CV_{events} when CV_{events} is large, here we consider the correlation between $\Delta w_{a,p}$(diff) and $\Delta w_{a,d}$(diff)

$$\rho_{PD}(\text{diff}) = \text{Corr}_a(\Delta w_{a,p}(\text{diff}), \Delta w_{a,d}(\text{diff}))$$

and set $\tau_{delay} = 0$ and $\tau_{cross} \ll \tau_{STDP}$ for simplicity.

By definition,

$$\rho_{PD}(\text{diff}) = \text{Corr}_a(\sum_s N_{a,s} z_{s,p}, \sum_s N_{a,s} z_{s,d}).$$

In this equation, s is the index for synchronous events. $N_{a,s}$ is the spike number of the ath non-central neuron in the sth synchronous event, and $z_{s,p}$ ($z_{s,d}$) is the potentiation (depression) per spike caused by pairing these $N_{a,s}$ non-central spikes with the central spikes that *not* in the sth synchronous event.

Using the approximation similar to **eq.8**, it is easy to show that (note that $\tau_{delay} = 0$ in our calculation)

$$z_{s,p} = A_p \sum_{l=1}^{\infty} N_{0,s+l} \exp(-\frac{\tau_l}{\tau_{STDP}})$$

$$z_{s,d} = -A_d \sum_{l=1}^{\infty} N_{0,s+l} \exp(-\frac{\tau_l}{\tau_{STDP}})$$

with $N_{0,s}$ being the spike number of the central neuron in the sth synchronous event, $\{\tau_l\}$ ($l = 0, 1, \cdots$) being a Gamma process with rate r_0/p and coefficient of variance CV_{events} starting from $\tau_0 = 0$.

Therefore,

$$\rho_{PD}(\text{diff}) = \text{Corr}_a(\sum_s N_{a,s} z_{s,p}, \sum_i N_{a,s} z_{s,d}) = \frac{\mathcal{X} - \mathcal{Y} - \mathcal{Z}}{2\sqrt{\mathcal{X} \cdot \mathcal{Y}}}$$

with

$$\mathcal{X} = \text{Var}_a(\sum_s N_{a,s}(z_{s,p} + z_{s,d})),$$

$$\mathcal{Y} = \text{Var}_a(\sum_s N_{a,s} z_{s,p}),$$

$$\mathcal{Z} = \text{Var}_a(\sum_i N_{a,s} z_{s,d}).$$

In our model (Model Sync-Auto 2 in **Section 4.2**), $N_{a,s}$ are independent with each other, and $\text{Var}_a(N_{a,s}) = p$. Therefore, if we let $A_p = A_d = A$, we will have

$$\mathcal{X} = \text{Var}_a(\sum_s N_{a,s}(z_{s,p} + z_{s,d}))$$

$$\propto A^2 \text{Var}_a(N_{a,s}) \cdot \{p^2 \text{E}_T[(\sum_{l=1}^{\infty} \exp(-\frac{\tau_l}{\tau_{STDP}}) - \sum_{l=1}^{\infty} \exp(-\frac{\tau_l}{\tau_{STDP}}))^2] + p\text{E}_T[2\sum_{l=1}^{\infty} \exp(-\frac{2\tau_l}{\tau_{STDP}})]\}$$

$$\propto p^2 \text{E}_T[(\sum_{l=1}^{\infty} \exp(-\frac{\tau_l}{\tau_{STDP}}) - \sum_{l=1}^{\infty} \exp(-\frac{\tau_l}{\tau_{STDP}}))^2] + p\text{E}_T[2\sum_{l=1}^{\infty} \exp(-\frac{2\tau_l}{\tau_{STDP}})]$$

$$\mathcal{Y} = \mathrm{Var}_a(\sum_s N_{a,s} z_{s,p})$$

$$\propto A^2 \frac{1}{2}\mathrm{Var}_a(N_{a,s}) \cdot \{p^2 \mathrm{E}_T[(\sum_{l=1}^{\infty} \exp(-\frac{\tau_l}{\tau_{STDP}}))^2] + p\mathrm{E}_T[\sum_{l=1}^{\infty} \exp(-\frac{2\tau_l}{\tau_{STDP}})]$$

$$+p^2 \mathrm{E}_T[(\sum_{l=1}^{\infty} \exp(-\frac{\tau_l}{\tau_{STDP}}))^2] + p\mathrm{E}_T[\sum_{l=1}^{\infty} \exp(-\frac{2\tau_l}{\tau_{STDP}})]\}$$

$$\propto \frac{1}{2}p^2 \mathrm{E}_T[(\sum_{l=1}^{\infty} \exp(-\frac{\tau_l}{\tau_{STDP}}))^2 + (\sum_{l=1}^{\infty} \exp(-\frac{\tau_l}{\tau_{STDP}}))^2] + \frac{1}{2}p\mathrm{E}_T[2\sum_{l=1}^{\infty} \exp(-\frac{2\tau_l}{\tau_{STDP}})]$$

and

$$\mathcal{Z} = \mathrm{Var}_a(\sum_i N_{a,s} z_{s,d})$$

$$\propto \frac{1}{2}p^2 \mathrm{E}_T[(\sum_{l=1}^{\infty} \exp(-\frac{\tau_l}{\tau_{STDP}}))^2 + (\sum_{l=1}^{\infty} \exp(-\frac{\tau_l}{\tau_{STDP}}))^2] + \frac{1}{2}p\mathrm{E}_T[2\sum_{l=1}^{\infty} \exp(-\frac{2\tau_l}{\tau_{STDP}})]$$

Armed with these results, it is easy to show that

$$\rho_{PD}(\mathrm{diff}) = -\frac{p\mathcal{A}^2}{p\mathcal{B} + \mathcal{C}} \quad (33)$$

with

$$\mathcal{A} = \mathrm{E}_T[\sum_{l=1}^{\infty} \exp(-\frac{\tau_l}{\tau_{STDP}})] \quad (34)$$

$$\mathcal{B} = \mathrm{E}_T[(\sum_{l=1}^{\infty} \exp(-\frac{\tau_l}{\tau_{STDP}}))^2] \quad (35)$$

$$\mathcal{C} = \mathrm{E}_T[\sum_{l=1}^{\infty} \exp(-\frac{2\tau_l}{\tau_{STDP}})] \quad (36)$$

Our analytic calculation well predict the numeric results (**Supplementary Figure 10C**).

Now let's understand the change of $\rho_{PD}(\mathrm{diff})$ with CV_{events}. By definition, $\mathcal{B} > \mathcal{C}$, so we can write the denominator of **eq.33** as $(p+a)\mathcal{B}$ with $0 < a < 1$. From **Supplementary Figure 10D left**, we can see that $a \approx 1$ when CV_{events} is small, and gradually decreases when CV_{events} gets large. Therefore, if p is relatively large, then from **eq.33**

$$\rho_{PD}(\mathrm{diff}) \tilde{\propto} -\frac{\mathcal{A}^2}{\mathcal{B}}$$

with $\tilde{\propto}$ representing "approximately propotional to". What's more,

$$-\frac{\mathcal{A}^2}{\mathcal{B}} = -\frac{1}{\frac{\mathcal{B}-\mathcal{A}^2+\mathcal{A}^2}{\mathcal{A}^2}} = -\frac{1}{\frac{\mathrm{Var}_T[\sum_{l=1}^{\infty} \exp(-\frac{\tau_l}{\tau_{STDP}})]}{\mathrm{E}_T[\sum_{l=1}^{\infty} \exp(-\frac{\tau_l}{\tau_{STDP}})]^2} + 1}$$

Therefore, the change of $\rho_{PD}(\mathrm{diff})$ basically reflects the change of $\frac{\mathrm{s.d.}}{\mathrm{mean}}$ ratio of $\sum_{l=1}^{\infty} \exp(-\frac{\tau_l}{\tau_{STDP}})$ (compare **Supplementary Figure 10C** with **10D right**).

When CV_{events} is small, $\mathcal{A}$, $\mathcal{B}$ and $\mathcal{C}$ are all small, which makes $\mathrm{Var}_a(\Delta w_{a,p}(\mathrm{diff}))$ and $\mathrm{Var}_a(\Delta w_{a,d}(\mathrm{diff}))$ are

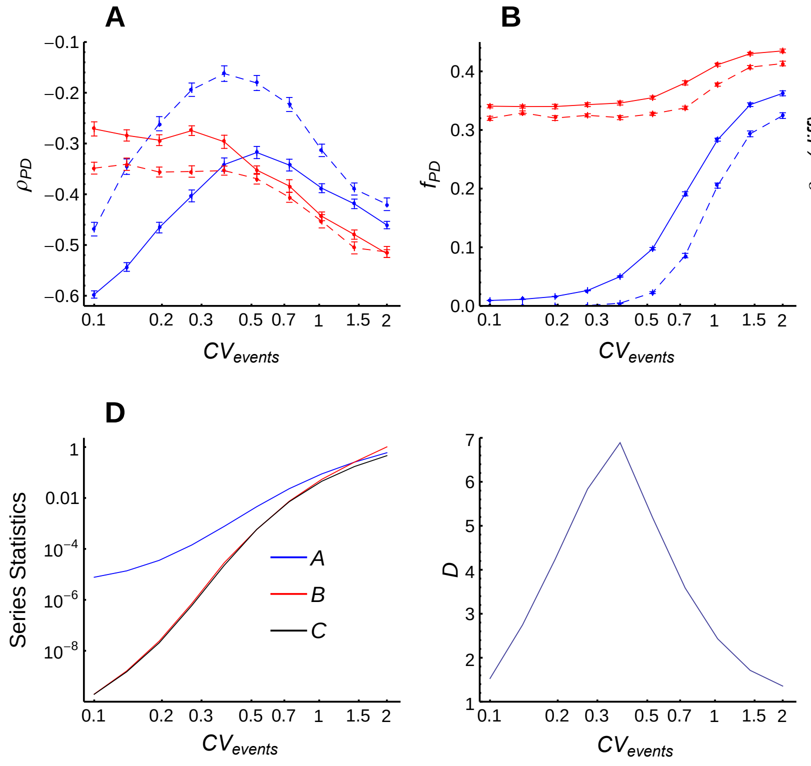

Figure 10: **Understanding the influence of CV_{events} to c_{II}.** (**A**) ρ_{PD} as a function of CV_{events} when τ_{cross} = 0.5ms (blue) or 2.5ms (red) and $p = 2$ (solid line) or 5 (dashed line). (**B**) The same as **A**, but for f_{PD}. In **A-B**, spike patterns are generated according to Model Sync-Auto 2 in **Section 4.2**, and $\tau_{delay} = 1$ms. (**C**) ρ_{PD}(diff) as a function of CV_{events}. The dots with error bars are simulation results, while the line is calculated from **eq.33.** Spike patterns are also generated according to Model Sync-Auto 2, but $\tau_{delay} = 0$**ms.** (**D**) Statistics of the series $\{\exp(-\frac{\tau_l}{\tau_{STDP}})\}$ $(l = 1, 2, 3, \cdots)$ as a function of CV_{events}, with $\{\tau_l\}$ $(l = 0, 1, \cdots)$ being a Gamma process with rate r_0/p (here we set $r_0 = 20$Hz, $p = 5$) and coefficient of variance CV_{events} starting from $\tau_0 = 0$. $\mathcal{A}$, $\mathcal{B}$ and $\mathcal{C}$ in the left panel are defined in **eqs.34-36**, and $\mathcal{D}$ in the right panel is the $\frac{\text{s.d.}}{\text{mean}}$ ratio of $\sum_{l=1}^{\infty} \exp(-\frac{\tau_l}{\tau_{STDP}})$. In **A-C**, the parameters for STDP, the size of converging motifs as well as simulation time and trials are the same as in **Figure 4** in the main text, error bars represent s.e.m. In **D**, the results were from averaging over 100000 trials of Gamma processes.

small; so $\Delta w_{a,p}(\text{diff})$ and $\Delta w_{a,d}(\text{diff})$ plays a small role in the value of $\rho_{PD} = \text{Corr}_a(\Delta w_{a,p}, \Delta w_{a,d})$ (see **eqs.31-32** again for the meaning of $\Delta w_{a,p}(\text{diff})$ and $\Delta w_{a,d}(\text{diff})$ as well as $\Delta w_{a,p}$ and $\Delta w_{a,d}$). In this case, the interaction of central and non-central spikes that belong to the *same* synchronous event dominates. However, when CV_{events} gets large, $\Delta w_{a,p}(\text{diff})$ and $\Delta w_{a,d}(\text{diff})$ get strong, and our calculation on $\rho_{PD}(\text{diff})$ helps to gain insight on ρ_{PD} in this case: the decrease of $\frac{\text{s.d.}}{\text{mean}}$ ratio of $\sum_{l=1}^{\infty} \exp(-\frac{\tau_l}{\tau_{STDP}})$ seems to be the key reason for the uniform decrease of ρ_{PD} when $CV_{events} > 0.7$ (**Supplementary Figure 10A**).

The coupling factor f_{PD} significantly increases with CV_{events}, especially when $\tau_{cross} \leq \tau_{delay}$ (**Supplementary Figure 10B**). As $c_{II} = 1 + 2\rho_{PD} f_{PD}$, and $\rho_{PD} < 0$, the increase of f_{PD} also contributes to the decrease of c_{II}. By definition,

$$f_{PD} = \frac{\sqrt{\text{Var}_a(\Delta w_{a,p}) \cdot \text{Var}_a(\Delta w_{a,d})}}{\text{Var}_a(\Delta w_{a,p}) + \text{Var}_a(\Delta w_{a,d})}$$
$$= \frac{1}{2}\sqrt{1 - [\frac{\text{Var}_a(\Delta w_{a,p}) - \text{Var}_a(\Delta w_{a,d})}{\text{Var}_a(\Delta w_{a,p}) + \text{Var}_a(\Delta w_{a,d})}]^2},$$

therefore the increase of f_{PD} reflects the decrease of the difference of $\text{Var}_a(\Delta w_{a,p})$ and $\text{Var}_a(\Delta w_{a,d})$ relative to their total value. From the equation above, we know that the large difference between $\text{Var}_a(w_{a,p})$ and $\text{Var}_a(w_{a,d})$ is the reason why f_{PD} is small when CV_{events} is small (**Supplementary Figure 10B**). From our discussions above, we know that when CV_{events} is small, both $\text{Var}_a(w_{a,p}(\text{diff}))$ and $\text{Var}_a(w_{a,d}(\text{diff}))$ are small, so $\text{Var}_a(\Delta w_{a,p})$ and $\text{Var}_a(\Delta w_{a,d})$ are largely determined by $\text{Var}_a(w_{a,p}(\text{same}))$ and $\text{Var}_a(w_{a,d}(\text{same}))$ in this case, which is the variance caused by the interaction of central and non-central spikes that belong to the same synchronous event. In this case, the large difference between $\text{Var}_a(w_{a,p})$ and $\text{Var}_a(w_{a,d})$ reflects the large difference between $\text{Var}_a(w_{a,p}(\text{same}))$ and $\text{Var}_a(w_{a,d}(\text{same}))$. This difference always exists as long as $\tau_{delay} \neq 0$, but is particularly strong when $\tau_{cross} \leq \tau_{delay}$, because in this case $\text{Var}_a(w_{a,p}(\text{same}))/N_s \approx 0$ (with N_s being the number of synchronous events in the spike pattern), and it is easy to show that $\text{Var}_a(w_{a,d}(\text{same}))/N_s \approx p^2(p+1)\exp(-\frac{2\tau_{delay}}{\tau_{cross}})$. When CV_{events} increases, both $\text{Var}_a(w_{a,p}(\text{diff}))$ and $\text{Var}_a(w_{a,d}(\text{diff}))$ increase, and as τ_{delay} is small comparing to inter-event interval, $\text{Var}_a(w_{a,p}(\text{diff})) \approx \text{Var}_a(w_{a,d}(\text{diff}))$. This reduces the difference of $\text{Var}_a(\Delta w_{a,p})$ and $\text{Var}_a(\Delta w_{a,d})$ relative to their total value, which is the reason for the increase of f_{PD} with CV_{events}.

5 Classifying Auto-correlation Structure under Synchronous Firing Using Rescaled Time Transform

As mentioned in **Section 3.4** of the main text, auto-correlation structure may come into spike patterns with synchronous firing in three ways:

1) The broadness of the distribution of spike number per neuron per synchronous event ($\text{AT}_{\text{SpikeNum}}$).
2) The burstiness/regularity of the pieces of spike trains within synchronous events ($\text{AT}_{\text{WithinEvent}}$).
3) The burstiness/regularity of the occurrence of synchronous events ($\text{AT}_{\text{events}}$).

As we mentioned in the main text, using rescaled time transform (**Figure 10** of the main text), Auto-correlation structure under synchronous firing can be classified into two classes: the factors that contributes to $CV_{rescale}$ that does not influence P-D imbalance, which thereby do not contribute to DriftV under heterogeneity of rates, and the factors that contributes to CV_{events} that influences P-D imbalance, which thereby contribute to DriftV under

heterogeneity of rates.

It is easy to think that

1) CV_{events} is influenced by $\mathrm{AT}_{\mathrm{events}}$.

2) $CV_{rescale}$ is influenced by $\mathrm{AT}_{\mathrm{SpikeNum}}$ and $\mathrm{AT}_{\mathrm{WithinEvent}}$.

The first point above is apparent. In this section, we will give numeric evidences on the second point above, and on the influence of $CV_{rescale}$ and CV_{events} onto P-D imbalance.

5.1 The factors that change $CV_{rescale}$

Given a spike pattern, we calculate $CV_{rescale}$ like this: we first order all the spikes in the pattern, then the $CV_{rescale}$ of a neuron is defined as the CV value of the indexes of the spikes of the neuron, then the $CV_{rescale}$ of the neuronal population is defined as the averaged $CV_{rescale}$ over all the neurons which fired more than 3 spikes during the simulation time.

We generated spike trains using Model Sync-Auto 1 (see **Section 4.1**), so that we could explicitly control both $\mathrm{AT}_{\mathrm{SpikeNum}}$ and $\mathrm{AT}_{\mathrm{WithinEvent}}$. We can see that both of these two factors tend to increase $CV_{rescale}$, but they hardly change P-D imbalance (**Supplementary Figure 11AB**). On the contrary, CV_{events} can hardly influence $CV_{rescale}$, but may strongly change P-D imbalance especially when $CV_{events} > 1$ (**Supplementary Figure 11CD**).

5.2 The influence of CV_{events} on P-D imbalance

To understand how CV_{events} influences P-D imbalance, we generated spike trains using Model Sync-Auto 2 (see **Section 7**), and study how $\mathrm{E}_{a,T}(\Delta w_a)$ changes with CV_{events}. We found that this influence may be a little complicated (**Supplementary Figure 12**):

1) Suppose during a synchronous event $\mathcal{S}_0$, the central neuron fires at time t_0. Because of the axonal delay τ_{delay}, there is usually a time interval between t_0 and when the spikes from non-central neurons arrive at the axonal terminal, and the typical length of this interval is τ_{delay}. If synchronous events are *not* close to each other, so that no non-central spikes from synchronous events *other than* $\mathcal{S}_0$ arrive at the central neuron during this interval (i.e. different synchronous events do not *overlap* with each other), then $\mathrm{E}_{a,T}(\Delta w_a)$ will increases (or decreases) with CV_{events} if $A_p \exp(\tau_{delay}/\tau_{STDP}) > A_d \exp(-\tau_{delay}/\tau_{STDP})$ (or $A_p \exp(\tau_{delay}/\tau_{STDP}) < A_d \exp(-\tau_{delay}/\tau_{STDP})$). In this paper, typically $\tau_{delay} < \tau_{STDP}$, so these conditions become $A_p > A_d$ or $A_p < A_d$.

2) If synchronous events are allowed to overlap with each other, then CV_{events} will increase the chance of this overlapping when it is too large (typically when $CV_{events} > 1$). In this case, $\mathrm{E}_{a,T}(\Delta w_a)$ will decrease (or increase) with CV_{events} if $\tau_{delay} > 0$ (or $\tau_{delay} < 0$).

5.2.1 The case when synchronous events do not overlap with each other

Consider a central spike at time t_0 that belongs to a synchronous event $\mathcal{S}_0$. CV_{events} controlls the burstiness of the occurrence of synchronous events, which influences the efficacy variability as well as P-D imbalance by changing the STDP interaction of t_0 with the non-central spikes in the synchronous events *other than* $\mathcal{S}_0$. Suppose a synchronous event $\mathcal{S}_-$ happens before $\mathcal{S}_0$, then if $\mathcal{S}_-$ and $\mathcal{S}_0$ do not overlap with each other, then because of $\tau_{cross} \ll \tau_{STDP}$, the interaction between t_0 and a non-central spike in $\mathcal{S}_-$ will potentiate the synapse approximately by $A_p \exp(-\frac{t_0-(\bar{t}_-+\tau_{delay})}{\tau_{STDP}})$, with $\bar{t}_-$ being the mean spike time of $\mathcal{S}_-$. Similarly, if a synchronous event $\mathcal{S}_+$ happens after $\mathcal{S}_0$, then the interaction between t_0 and a non-central spike in $\mathcal{S}_+$ will depress the synapse approximately by

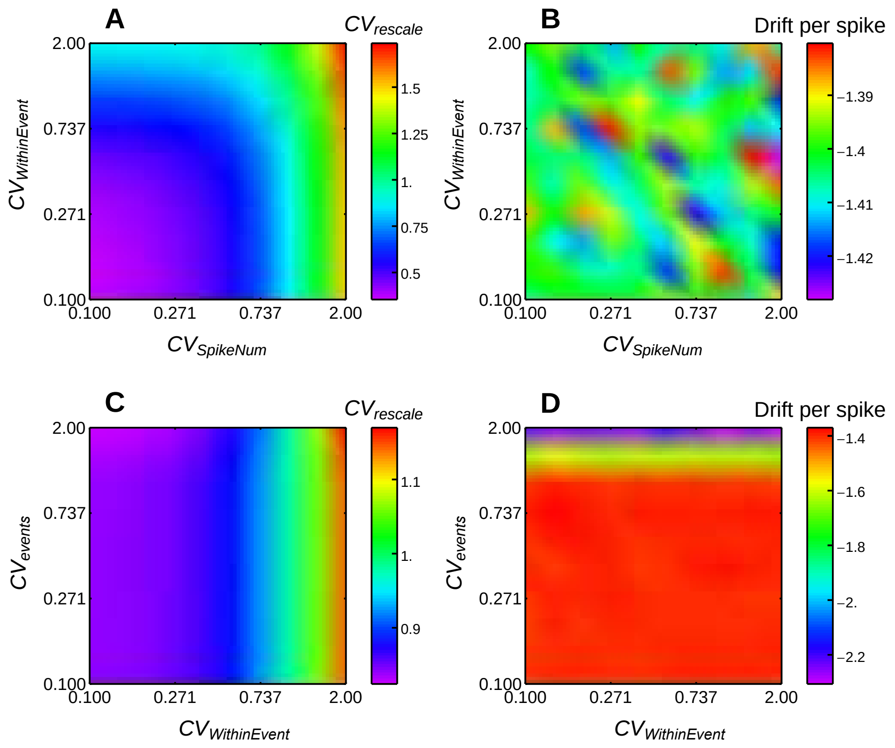

Figure 11: **How $\mathbf{AT_{SpikeNum}}$ and $\mathbf{AT_{WithinEvent}}$ change** $CV_{rescale}$ **and P-D imbalance.** (**A**) $CV_{rescale}$ as a function of $CV_{SpikeNum}$ and $CV_{WithinEvent}$ when $p = 2$ and $\tau_{cross} = 2$ms. Spike trains are generated using Model Sync-Auto 1 (**Section 4.1**). Note that both $CV_{SpikeNum}$ and $CV_{WithinEvent}$ are drawn in log scale. (**B**) The same as **A**, but for drift per spike $(\mathrm{E}_{a,T}(\Delta w_a)/\bar{N}_0$, with $\bar{N}_0$ being the trial-averaged spike number of the central neuron), which quantifies P-D imbalance. Note that drift per spike hardly changes with $CV_{SpikeNum}$ and $CV_{WithinEvent}$. (**C**) $CV_{rescale}$ as a function of $CV_{SpikeNum}$ and CV_{event} when $p = 2$ and $\tau_{cross} = 2$ms. Spike trains are generated using Model Sync-Auto 2 (**Section 4.2**). (**D**) The same as **C**, but for drift per spike. Note that drift per spike hardly changes with $CV_{pattern}$, but changes significantly with CV_{event} especially when $CV_{event} > 1$. In **A-D**, the size of the converging motif, the parameters for STDP, the parameters for synaptic homeostasis and the simulation time and trials are the same as in **Figure 4** in the main text. Error bars represent s.e.m.

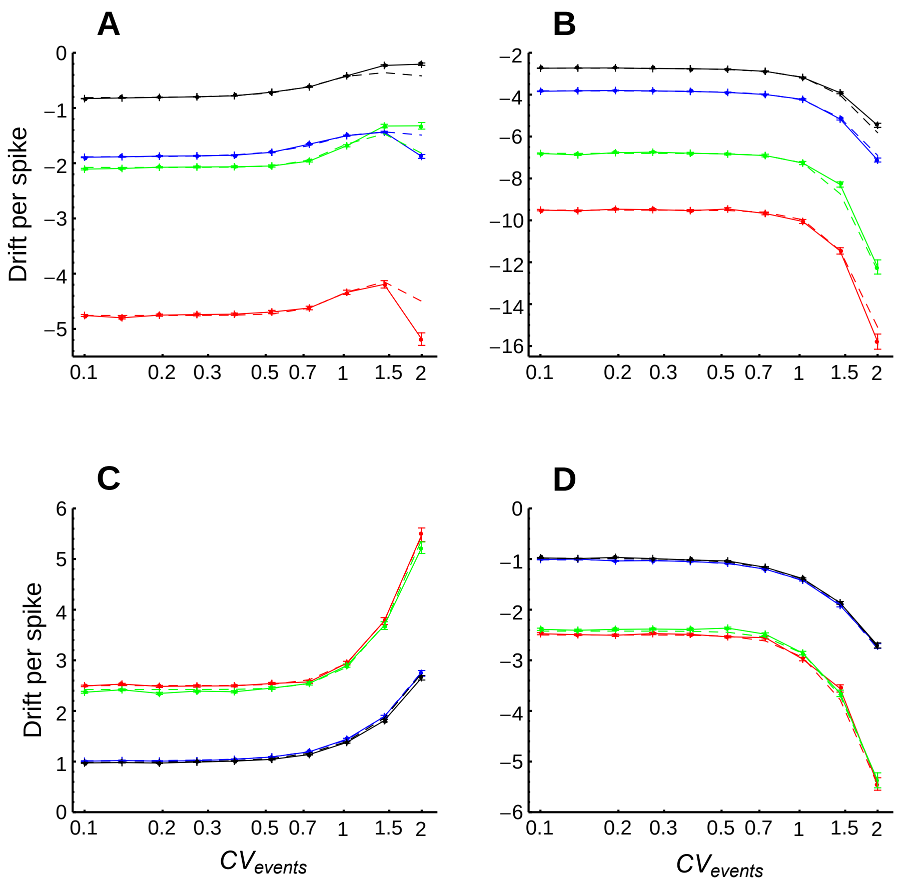

Figure 12: **How CV_{events} influences P-D imbalance.** (**A**) Drift per spike ($\mathrm{E}_{a,T}(\Delta w_a)/\bar{N}_0$, with $\bar{N}_0$ being the trial-averaged spike number of the central neuron) as a function of CV_{events} when $\tau_{cross} = 0.5$ms and $p = 2$ (blue), or $\tau_{cross} = 0.5$ms and $p = 5$ (red), or $\tau_{cross} = 2.5$ms and $p = 2$ (black), or $\tau_{cross} = 2.5$ms and $p = 5$ (green), under $A_p = 2$, $A_d = 1$, $\tau_{delay} = 1$ms. The spike trains are generated using Model Sync-Auto 2 (**Section 4.2**). The dots with error bars represent simulation results, the solid lines connecting the dots are to guide eyes, the dashed lines are the results calculated from **eq.37-eq.41**. (**B**) The same as **A**, but under $A_p = 1$, $A_d = 2$, $\tau_{delay} = 1$ms. (**C**) The same as **A**, but under $A_p = 2$, $A_d = 1$, $\tau_{delay} = 0$ms. (**D**) The same as **A**, but under $A_p = 1$, $A_d = 2$, $\tau_{delay} = 0$ms. In **A-D**, the size of the converging motif, as well as the simulation time and trials are the same as in **Figure 4** in the main text. Error bars represent s.e.m.

$A_d \exp(-\frac{(\bar{t}_+ + \tau_{delay}) - t_0}{\tau_{STDP}})$. After considering all the non-central spikes in all the synchronous events other than $\mathcal{S}_0$, we have that the trial-averaged change of the ath synapse caused by the interaction of t_0 with the spikes of the ath non-central neuron in all the synchronous events other than $\mathcal{S}_0$ is

$$\sum_{k=p,d} \mathrm{E}_T[\sum_{j \notin \mathcal{S}_0} \Delta w_{a,k}(t_0, t_j)] \approx p[A_p \exp(\frac{\tau_{delay}}{\tau_{STDP}}) - A_d \exp(-\frac{\tau_{delay}}{\tau_{STDP}})]\mathrm{E}_T[\sum_{l=1}^{\infty} \exp(-\frac{\tau_l}{\tau_{STDP}})],$$

with p being the mean spike number per neuron per synchronous event, and τ_l being the duration of the interval between $\mathcal{S}_0$ and the lth synchronous event before or after it. In Model Sync-Auto 2 (see **Section 7**), we use Gamma process of rate r_0/p (with r_0 being the firing rate of a neuron) and coefficient of variance CV_{events} to model the occurrence of synchronous events. Therefore, $\{\tau_l\}$ $(l = 0, 1, \cdots)$ is a Gamma process with rate r_0/p and coefficient of variance CV_{events}, starting from $\tau_0 = 0$. Although Gamma process does not avoid synchronous-events overlap, such overlap is rare when $CV_{events} < 1$. Therefore, we can still gain understanding on this no-overlap case by investigating the case when $CV_{events} < 1$. From **Supplementary Figure 10D**, we see that $\mathcal{A} = \mathrm{E}_T[\sum_{l=1}^{\infty} \exp(-\frac{\tau_l}{\tau_{STDP}})]$ continuously increases with CV_{events}. From the equation above, this suggests that $\sum_{k=p,d} \mathrm{E}_T[\sum_{j \notin \mathcal{S}_0} \Delta w_{a,k}(t_0, t_j)]$ increases with CV_{events} when $A_p \exp(\frac{\tau_{delay}}{\tau_{STDP}}) > A_d \exp(-\frac{\tau_{delay}}{\tau_{STDP}})$, but decreases with CV_{events} when $A_p \exp(\frac{\tau_{delay}}{\tau_{STDP}}) < A_d \exp(-\frac{\tau_{delay}}{\tau_{STDP}})$.

Because CV_{events} does not change the STDP interaction between t_0 and the non-central spikes in $\mathcal{S}_0$, the change of $\sum_{k=p,d} \mathrm{E}_T[\sum_{j \notin \mathcal{S}_0} \Delta w_{a,k}(t_0, t_j)]$ with CV_{events} also reflect the total change of the ath synapse.

5.2.2 The case when different synchronous events are allowed to overlap with each other

We will focus on the case when $\tau_{delay} > 0$ in the following discussions, the case when $\tau_{delay} < 0$ can be similarly understood.

Consider a central spike at time t_0, and suppose that this spike belongs to a synchronous event $\mathcal{S}_0$ whose mean spike time is at $\bar{t}_0$. Because of the axonal delay τ_{delay}, the non-central spikes in $\mathcal{S}_0$ typically arrives at the central neuron at around $\bar{t}_0 + \tau_{delay}$. If another synchronous events $\mathcal{S}_{-1}$ happens immediately before $\mathcal{S}_0$, then it is possible that during the time interval between t_0 and $\bar{t}_0 + \tau_{delay}$, the non-central spikes in $\mathcal{S}_{-1}$ also arrive at the central neuron. In this case, these non-central spikes in $\mathcal{S}_{-1}$ will depress the synapses through their interactions with t_0.

In Model Sync-Auto 2 (see **Section 7**), we use Gamma process of coefficient of variance CV_{events} to model the occurrence of synchronous events. In this model, such synchronous-events overlap often occurs when CV_{events} is large (typically $CV_{events} > 1$), thereby depressing the synapses. In the following discussions, we will perform analytic calculations on $\mathrm{E}_T(\Delta w_a)$ when the neurons in converging motifs fire according to the spike patterns generated by this model, compare the results with simulations, thereby understanding the effect of the synchronous-events overlap.

It is easy to show that the contribution to synaptic changes by the interaction between t_0 and $\mathcal{S}_0$ is:

1) when $\tau_{cross} \leq \tau_{delay}$,

$$\mathrm{E}_T[\sum_{j \in \mathcal{S}_0} \Delta w_{a,p}(t_0, t_j)] = 0,$$

$$\mathrm{E}_T[\sum_{j \in \mathcal{S}_0} \Delta w_{a,d}(t_0, t_j)] \approx -A_d p \exp(-\frac{\tau_{delay}}{\tau_{STDP}});$$

2) when $\tau_{delay} > \tau_{cross}$,

$$\mathrm{E}_T[\sum_{j\in\mathcal{S}_0}\Delta w_{a,p}(t_0,t_j)]$$

$$\approx \frac{pA_p}{\tau_{cross}^2}\exp(-\frac{\tau_{cross}-\tau_{delay}}{4\tau_{STDP}})\frac{1}{2}(\tau_{cross}-\tau_{delay})^2, \quad (37)$$

$$\mathrm{E}_T[\sum_{j\in\mathcal{S}_0}\Delta w_{a,d}(t_0,t_j)]$$

$$\approx -\frac{pA_d}{\tau_{cross}}\exp(-\frac{\tau_{cross}+\tau_{delay}}{2\tau_{STDP}})\tau_{delay} - \frac{pA_d}{\tau_{cross}^2}\exp(-\frac{\tau_{cross}+\tau_{delay}}{4\tau_{STDP}})\cdot\frac{1}{2}(\tau_{cross}^2-\tau_{delay}^2). \quad (38)$$

Now we consider the synchronous events other than $\mathcal{S}_0$. Suppose that the synchronous event immediately before $\mathcal{S}_0$ is $\mathcal{S}_{-1}$, and the synchronous event immediately after $\mathcal{S}_0$ is $\mathcal{S}_1$, then under the approximation that $\tau_{cross}\to 0$,

$$\mathrm{E}_T[\sum_{j\in\mathcal{S}_{-1}\cup\mathcal{S}_1}\Delta w_{a,d}(t_0,t_j)] \approx -\int_0^{\tau_{delay}} q(x)\cdot pA_d\exp(-\frac{\tau_{delay}-x}{\tau_{STDP}})\mathrm{d}x - \int_0^{\infty} q(x)\cdot pA_d\exp(-\frac{x+\tau_{delay}}{\tau_{STDP}})\mathrm{d}x, \quad (39)$$

$$\mathrm{E}_T[\sum_{j\in\mathcal{S}_{-1}\cup\mathcal{S}_1}\Delta w_{a,p}(t_0,t_j)] \approx \int_{\tau_{delay}}^{\infty} q(x)\cdot pA_p\exp(-\frac{x-\tau_{delay}}{\tau_{STDP}})\mathrm{d}x, \quad (40)$$

with $q(x)=\frac{\beta^\alpha}{\Gamma(\alpha)}x^{\alpha-1}\exp(-\beta x)$ being the inter-event interval distribution with $\alpha = 1/CV_{events}^2$ and $\beta = r_0/(CV_{events}^2 p)$. If we suppose that the synchronous events other than $\mathcal{S}_{-1}$, $\mathcal{S}_0$ and $\mathcal{S}_1$ are far away from $\mathcal{S}_0$, so that the interval between them and t_0 is far larger than τ_{cross} and τ_{delay}, then their interaction with t_0 can be approximated by

$$\sum_{k=p,d}\mathrm{E}_T[\sum_{j\notin\mathcal{S}_{-1}\cup\mathcal{S}_0\cup\mathcal{S}_1}\Delta w_{a,k}(t_i,t_j)] \approx p(A_p-A_d)\mathrm{E}_T[\sum_{l=2}^{\infty}\exp(-\frac{\tau_l}{\tau_{STDP}})] \quad (41)$$

with $\{\tau_l\}$ $(l=0,1,\cdots)$ being a Gamma process with rate r_0/p and coefficient of variance CV_{events}, starting from $\tau_0 = 0$. Note that the summation over l in the equation above starts from $l=2$, which represents $\mathcal{S}_2$ for depression $(k=d)$ and $\mathcal{S}_{-2}$ for potentiation $(k=p)$.

Eq.37-eq.41 together give the approximation of $\sum_{k=p,d}\mathrm{E}_T[\sum_j\Delta w_{a,k}(t_0,t_j)]$, which can be solved numerically.

We compare the results of the calculations above with simulation results. We can see that our analytic calculation is able to qualitatively capture the change of $\mathrm{E}_T(\Delta w_a)$ with CV_{events} (**Supplementary Figure** 12). From **Supplementary Figure** 12, we can see that

1) Under the case $\tau_{delay} = 1$ms, if $A_p > A_d$ (or $A_p < A_d$),$\mathrm{E}_T(\Delta w_a)$ may increase (or decrease) with CV_{events} when CV_{events} is not large. But when CV_{events} is large (typically $CV_{events} > 1$), $\mathrm{E}_T(\Delta w_a)$ always decreases with CV_{events}, reflecting the synaptic depression caused by synchronous-events overlaps.

2) Under the case $\tau_{delay} = 0$ms, such synchronous-events overlap cannot happen. In this case, $\mathrm{E}_T(\Delta w_a)$ monotonically increases (or decrease) with CV_{events} if $A_p > A_d$ (or $A_p < A_d$).

These results suggest that if $\tau_{delay} > 0$, the synapses can be depressed by synchronous-events overlaps when CV_{events} is large.

When $\tau_{delay} < 0$, the dendritic delay for the post-synaptic spikes to arrive at the dendritic end is longer than the delay for the pre-synaptic spikes to arrive at the axonal terminal. In this case, the time when the central spike t_0 arrives at the dendritic end is typically later than the time when the non-central spikes in $\mathcal{S}_0$ arrives at the axonal

terminal by $|\tau_{delay}|$. If the synchronous events $\mathcal{S}_0$ and $\mathcal{S}_{-1}$ are close to each other, so that a non-central spike t_j in $\mathcal{S}_{-1}$ arrives at the axonal terminal during this interval, then the synapse will be potentiated by the interaction between t_0 and t_j. The analysis for $\tau_{delay} < 0$ is similar to the analysis above for $\tau_{delay} > 0$.

6 The Heterogeneity of Diffusion Strengths Caused by Heterogeneity of Rates and Heterogeneity of Cross-correlations

The main effect of heterogeneity of rates and heterogeneity of cross-correlations is to induce DriftV. They may also influence the diffusion of synapses, making different synapses have different diffusion strengths. As DriftV $\propto t^2$ and DiffV $\propto t$, DriftV will dominate in a long run as along as DriftV $\neq 0$. For completeness, we briefly discuss the heterogeneity of diffusion strengths under heterogeneity of rates and heterogeneity of cross-correlation, which may play an important role when DriftV ≈ 0.

Till now, our study is based on **eq.1** in the main text:

$$\text{TotalV} = \text{DiffV} + \text{DriftV},$$

with DiffV representing the average diffusion strength of all the synapses in the network. But the topic of this section goes into more details than DiffV, which investigates the heterogeneity of diffusion strengths caused by the heterogeneity of spike train statistics of different neurons.

We have already encountered a similar problem in **Section 2**, where we studied how the heterogeneity of rates of the non-central neuron influences DiffV. We did this by supposing that the firing rates of all the non-central neurons are uniformly r_s and the firing rate of the central neuron is kept at r_0, and then investigating how the diffusion strength of a converging motif changes with r_s (**Supplementary Figure 1B**, also see **eqs.6-7**). This strategy is general for studying the problem of heterogeneity of diffusion strengths. If the spike trains of the non-central neurons or the cross-correlations between the non-central neurons and the central neuron can be quantified by a set of parameters $\{p_1, p_2, \cdots, p_n\}$, then we can first suppose that the statistics of the spike patterns of all the non-central neurons are uniform and quantified by a dot $\mathcal{P}_s$ in the n-dimensional parameter space, and study how the diffusion strength changes with $\mathcal{P}_s$, while keeping the statistics of the central neuron unchanged.

We emphasize again that the heterogeneity of diffusion strengths is important for the learning process only when DrV ≈ 0.

6.1 The Heterogeneity of Diffusion Strengths Caused by Heterogeneity of Rates

As we discussed in **Section 3.5** of the main text, heterogeneity of rates induces DriftV by making use of P-D imbalance. Therefore, its contribution to DriftV is approximately zero when potentiation and depression almost balance with each other. In this case, its influence onto heterogeneity of diffusion strengths may become important. A similar situation has already been discussed in **Section 2**, in which spike trains are stationary processes with heterogeneity of rates, and by observing **Supplementary Figure 1B** we come to the conclusion that:

1) The diffusion strength tends to increase with the firing rate of the non-central neurons;

2) When $CV \ll 1$ (i.e. the spike trains are regular), the diffusion strength tends to sharply peak when the firing rate of the non-central neurons r_s and the firing rate of the central neuron r_0 are equal, and may also peak when $r_s = 2r_0$, $\frac{1}{2}r_0$ etc.

We already explain these phenomena in **Section 2**. The first factor is because that more non-central spikes induce more freedom to increase the synaptic variability during STDP. The second factor is due to transient cross-correlation. When synchronous firing is added into the spike pattern, transient cross-correlation may be fragile: as two pieces of spike trains in different synchronous events are hardly correlated with each other, the synaptic changes caused by two central spikes t_m and t_n (i.e. $\sum_j \Delta w_a(t_m, t_j)$ and $\sum_j \Delta w_a(t_n, t_j)$) are hard to be correlated if t_m and t_n belong to different synchronous events, even if m and n are nearby by index. The first factor, however, seems to be a general principle: the diffusion strength should be positively correlated with the firing rate of the non-central neurons.

6.2 The Heterogeneity of Diffusion Strengths Caused by Heterogeneity of Cross-correlations

From **eq.38** in the main text, we know that the DriftV caused by heterogeneity of cross-correlation is proportional to $r_0^2 \mathrm{Var}_a[r_a \int_{-\infty}^{\infty} \mathrm{d}\tau H(\tau) C_a(-\tau_{delay} - \tau)]$, with r_0 and r_a respectively being the firing rate of the central and ath non-central neuron, and $C_a(\tau)$ being the unit cross-correlation between them (with $\lim_{\tau\to\infty} C_a(\tau) = 1$). Therefore, DriftV $= 0$ if $\int_{-\infty}^{\infty} \mathrm{d}\tau H(\tau) C_a(-\tau_{delay} - \tau) = 0$ for every a, which may happen when $C_a(-\tau_{delay} - \tau)$ is strictly asymmetric around the STDP time window $H(\tau)$ (**Supplementary Figure 13**). In this case, $C_a(\tau)$ effectively induces synchronous firing between the central neuron and the ath non-central neuron, thus how diffusion strength change with $C_a(\tau)$ can be understood with the help of our results on synchronous firing after setting $\tau_{delay} = 0$.

Two important concepts of $C_a(\tau)$ are its strength (i.e. $\int_{-\infty}^{\infty} [C_a(\tau) - 1]\mathrm{d}\tau$) and its time scale of width:

1) The strength of $C_a(\tau)$ increases with the mean spike number p per neuron per synchronous event (it is easy to prove that $\int_{-\infty}^{\infty} [C_a(\tau) - 1]\mathrm{d}\tau = (p - r_0\tau_{cross})/r_0$ when the occurrence of synchronous events is Poisson). As DiffV increases with p (see **Section 3.3** in the main text and **Supplementary Figure 2A1, B1, C1**), diffusion strength increases with the strength of cross-correlation.

2) As we focused on the case that $\tau_{cross} \ll \tau_{STDP}$ when discussing synchronous firing, we did not explore the influence of τ_{cross} onto DiffV in details in this paper. However, it is not hard to understand the influence of the time scale of $C_a(\tau)$ onto diffusion strength when $\tau_{delay} = 0$. Suppose $C_1(\tau)$ and $C_2(\tau)$ have the same strength, but the time window of $C_1(\tau)$ is narrower than that of $C_2(\tau)$ (**Supplementary Figure 13**), then the single-step change of the 1st synapse will be larger than that of the 2nd synapse during STDP, therefore the 1st synapse tends to diffuse farther away from its initial value than the 2nd synapse.

Mathematically, $r_a C_a(\tau)$ means the probability density to find a spike of the ath non-central neuron at τ, given an central spike at time 0. If we suppose that the spike train of the ath non-central neuron is Poisson, then d (see **eq.13** in the main text) can be written as

$$d = \sum_{k=p,d} \mathrm{Var}_a(\sum_j \Delta w_{a,k}(t_i, t_j)) = (A_p^2 + A_d^2) \int_0^{\infty} \exp(-\frac{2t}{\tau_{STDP}}) r_a C_a(t) \mathrm{d}t$$

If we suppose that

$$C_a(t) = \frac{A_c}{\tau_c} \exp(-\frac{|t|}{\tau_c}) + 1$$

then

$$d = (A_p^2 + A_d^2) A_c r_a \cdot \frac{\tau_{STDP}}{2\tau_c + \tau_{STDP}} + (A_p^2 + A_d^2) r_a \frac{\tau_{STDP}}{2}$$

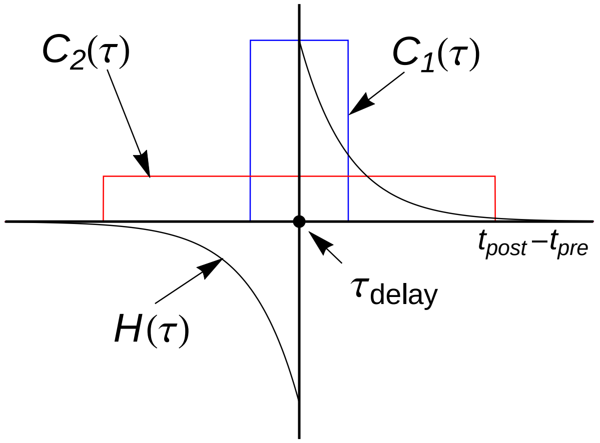

Figure 13: **Schematic on how heterogeneity of cross-correlations causes heterogeneity of diffusion strengths.** The STDP window is represented by the black curve. Two cross-correlations $C_1(\tau)$ and $C_2(\tau)$, indicated by the blue and red curve respectively, are symmetric around $H(\tau)$, but have different widths. Both of them cause zero drift velocity of synaptic efficacies, but the diffusion strength of the 1st synapse is stronger than that of the 2nd one.

we see that d increases with A_c but deceases with τ_c.

References

1. Cox, D. R. (1962). Renewal theory (London: Chapman and Hall)

2. Nawrot, M. P., Boucsein, C., Molina, V. R., Riehle, A., Aertsen, A., and Rotter, S. (2008). Measurement of variability dynamics in cortical spike trains. J. Neurosci. Methods 169, 374–390

3. Tuckwell, H. C. (1988). Introduction to theoretical neurobiology, vol. 2 (Cambridge: Cambridge University Press).
